# Interpretable Multimodal Fusion Model Enhances Postoperative Recurrence Prediction in Gastric Cancer

**DOI:** 10.1002/advs.202508190

**Published:** 2025-09-13

**Authors:** Ping'an Ding, Jiaxuan Yang, Sheng Chen, Honghai Guo, Jiaxiang Wu, Haotian Wu, Li Yang, Wenqian Ma, Yuan Tian, Renjun Gu, Lilong Zhang, Ning Meng, Xiaolong Li, Zhenjiang Guo, Yueping Liu, Lingjiao Meng, Qun Zhao

**Affiliations:** ^1^ The Third Department of Surgery the Fourth Hospital of Hebei Medical University Shijiazhuang Hebei 050011 China; ^2^ Hebei Key Laboratory of Precision Diagnosis and Comprehensive Treatment of Gastric Cancer Shijiazhuang Hebei 050011 China; ^3^ Big data analysis and mining application for precise diagnosis and treatment of gastric cancer Hebei Provincial Engineering Research Center Shijiazhuang Hebei 050011 China; ^4^ Clinical College of Hebei University Baoding Hebei 071000 China; ^5^ The Department of CT/MRI The Fourth Hospital of Hebei Medical University Shijiazhuang Hebei 050011 China; ^6^ Department of Endoscopy The Fourth Hospital of Hebei Medical University Shijiazhuang 050011 China; ^7^ School of Chinese Medicine & School of Integrated Chinese and Western Medicine Nanjing University of Chinese Medicine Nanjing Jiangsu 210023 China; ^8^ Department of Gastroenterology and Hepatology Jinling Hospital Medical School of Nanjing University Nanjing Jiangsu 210002 China; ^9^ Department of General Surgery Renmin Hospital of Wuhan University Wuhan Hubei 430065 China; ^10^ Department of General Surgery Shijiazhuang People's Hospital Shijiazhuang Hebei 050050 China; ^11^ Department of General Surgery Baoding Central Hospital Baoding Hebei 071030 China; ^12^ Department of General Surgery Hengshui People's Hospital Hengshui Hebei 053099 China; ^13^ Department of Pathology The Fourth Hospital of Hebei Medical University Shijiazhuang Hebei 050011 China; ^14^ Research Center and Tumor Research Institute of the Fourth Hospital of Hebei Medical University Shijiazhuang Hebei 050011 China

**Keywords:** deep learning, gastric cancer, pathomics, radiomics, recurrence prediction

## Abstract

Accurate prediction of early postoperative recurrence in locally advanced gastric cancer (LAGC) remains challenging due to tumor heterogeneity and limitations of traditional clinicopathological factors. This study aims to develop and validate an interpretable multimodal model for precise recurrence prediction. 1580 LAGC patients are enrolled from six Chinese medical centers and a multimodal fusion Risk Stratification Assessment (RSA) model integrating clinical, radiomic, and pathomic data is developed. Model performance is evaluated using internal, external, prospective, and public dataset validations. Transcriptome sequencing is conducted to elucidate biological mechanisms underlying recurrence. The RSA model significantly outperforms clinical‐only, radiomic‐only, and pathomic‐only models in predicting early recurrence, achieving area under the curve (AUC) values of 0.903 in the training cohort, 0.902 in internal validation, and ranging from 0.884 to 0.889 in external validations. Stratification by the RSA model consistently identifies high‐risk patients with significantly poorer five‐year survival across all cohorts (all P<0.001). Transcriptomic analysis reveals that high‐risk patients exhibit significant immune cell infiltration, increased expression of immune checkpoint molecules, and activation of immune‐related pathways, including interferon signaling and the IL‐6/JAK/STAT3 pathway. The integrated multimodal RSA model effectively predicts recurrence risk and prognosis in LAGC, enabling precise patient stratification and individualized postoperative management.

## Graphical abstract

An interpretable multimodal fusion model (RSA) integrating clinical, radiomic, and pathomic features is developed and validated to predict early postoperative recurrence in gastric cancer. The RSA model demonstrates superior predictive performance and holds potential for guiding adjuvant chemotherapy decisions and improving individualized patient management.

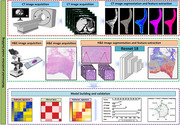

## 1 Introduction

Gastric cancer (GC) remains one of the leading causes of cancer‐related morbidity and mortality worldwide, with locally advanced gastric cancer (LAGC) being particularly challenging due to its high recurrence rate and poor postoperative prognosis.^[^ ^1^, ^2^ ^]^ Despite advances in surgical techniques, adjuvant therapies, and early detection, postoperative recurrence remains a major clinical issue, significantly impacting long‐term survival.^[^ ^3^, ^4^, ^5^ ^]^ LAGC patients are at high risk of both local and distant recurrences, which often occur within the first two years after surgery, leading to treatment failure and poor outcomes.^[^ ^6^, ^7^ ^]^ Accurate prediction of recurrence and prognosis is essential for optimizing individualized treatment strategies and follow‐up care. However, current predictive models are often limited by their inability to fully capture the tumor's molecular complexity and heterogeneity, leaving a significant gap in effective prognostication.^[^ ^8^, ^9^ ^]^

Traditional prognostic approaches in gastric cancer rely on clinicopathological factors such as tumor size, depth of invasion, histological grade, and lymph node involvement.^[^ ^10^, ^11^, ^12^, ^13^ ^]^ While these provide useful information, they often fail to accurately predict recurrence, as they do not capture the molecular and biological complexity of the disease. Tumor features like microvascular invasion, perineural invasion, and micrometastases, which significantly influence recurrence risk, are also difficult to assess using conventional methods.^[^ ^14^, ^15^, ^16^, ^17^ ^]^ Imaging technologies, including CT, MRI, and PET, are essential for detecting progression but are limited by low sensitivity to early or micrometastatic recurrences and an inability to provide molecular insights into the tumor's biology.^[^ ^18^, ^19^, ^20^, ^21^ ^]^ These challenges highlight the need for more comprehensive models that integrate multiple data types. Although machine learning and artificial intelligence hold promise in improving prediction, many models still struggle with data integration and lack interpretability, hindering their clinical applicability.^[^ ^22^, ^23^, ^24^, ^25^ ^]^

To address these limitations, we propose an interpretable multimodal fusion model that integrates clinical, pathological, and radiomic data to predict postoperative recurrence and prognosis in LAGC. By leveraging these complementary data sources, our model aims to enhance prediction accuracy and provide a more personalized approach to recurrence risk. Additionally, we conducted transcriptomic sequencing to deeply investigate the biological mechanisms underlying LAGC recurrence, offering valuable molecular insights that complement our multimodal approach. This fusion of clinical, imaging, and biological data enables a more precise, actionable model that not only predicts recurrence with greater accuracy but also provides deeper understanding of the biological drivers behind disease progression, ultimately guiding personalized treatment and improving patient outcomes.

## 2 Experimental Section

### 2.1 Study Cohort

**Figure 1:**
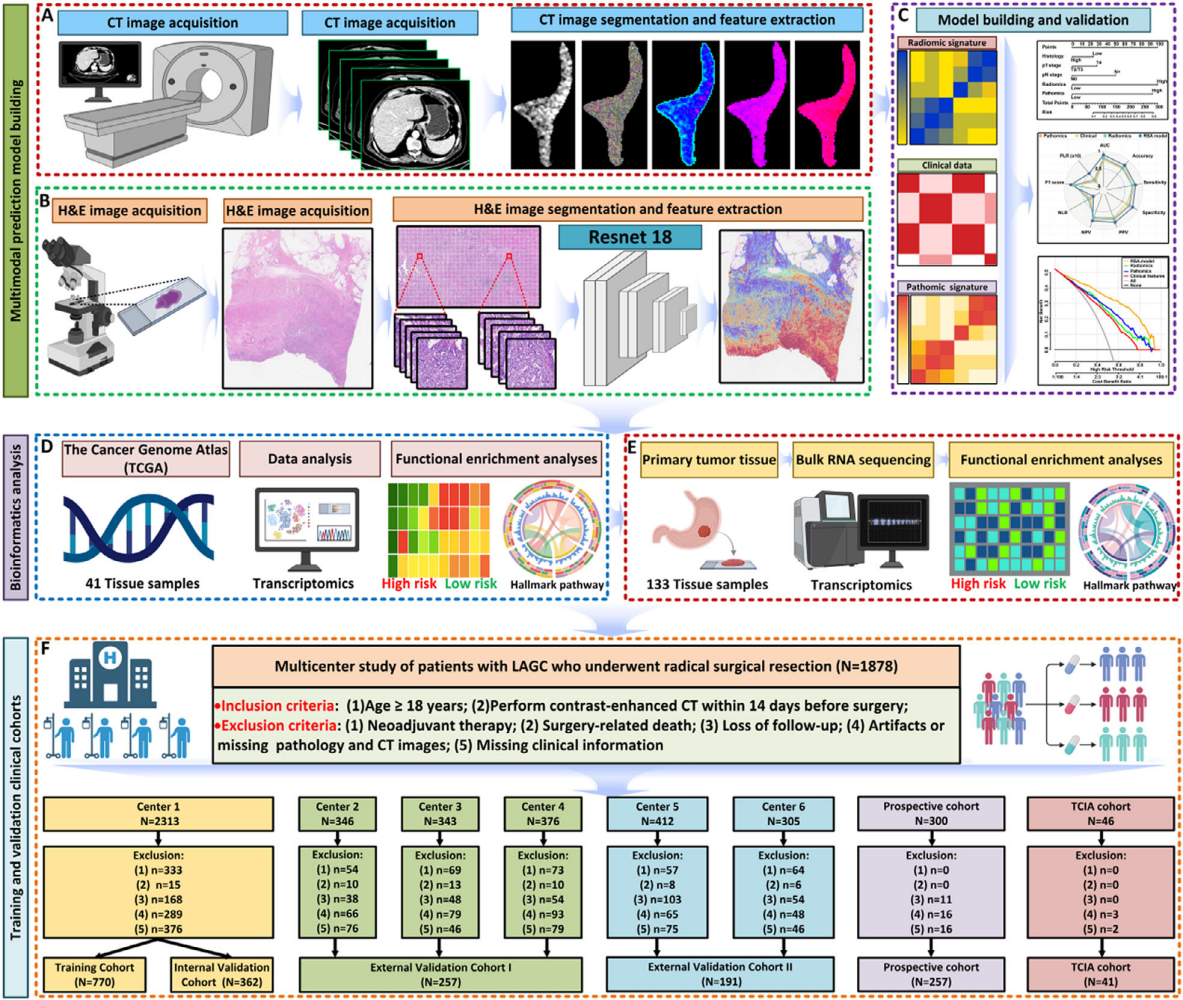
Study design flow chart. A) Radiomics Workflow: Manual segmentation following CT image acquisition, feature extraction, and construction of radiomic signatures. B) Pathomics Workflow: Selection of representative H&E‐stained image tiles, with ten randomly selected regions of interest per case, each measuring 1000 × 1000 pixels and containing the highest density of tumor cells. One pixel corresponds to 0.504 µm. C) Development and Validation of the RSA Model: A multimodal fusion model for predicting early recurrence in LAGC patients, along with performance evaluation. D) Bioinformatics Workflow for Public Dataset: Transcriptomic analysis of 41 patients from the TCIA cohort was conducted to explore the biological characteristics and immune infiltration associated with identified features, reflecting tumor heterogeneity in LAGC. E) Bioinformatics Workflow for Prospective Cohort: RNA sequencing was performed on 133 prospectively collected tissue samples, followed by bioinformatics analyses to characterize feature‐associated biology and immune profiles in LAGC tumors. F) Patient Cohort: A total of 1878 eligible patients with locally advanced gastric cancer were enrolled from six medical centers in China and a public dataset. All patients had preoperative abdominal CT scans and postoperative H&E‐stained pathological images. Abbreviations: Center 1 = The Fourth Hospital of Hebei Medical University; Center 2 = Shijiazhuang People's Hospital; Center 3 = Baoding Central Hospital; Center 4 = Hengshui People's Hospital; Center 5 = Jinling Hospital, Nanjing; Center 6 = Renmin Hospital of Wuhan University.

Patients with LAGC diagnosed between January 2012 and December 2019 were retrospectively enrolled from six participating medical centers. Among them, four were located in northern China: The Fourth Hospital of Hebei Medical University (FHHMU), Shijiazhuang People's Hospital (SJZPH), Baoding Central Hospital (BDCH), and Hengshui People's Hospital (HSPH). The remaining two centers were based in southern China, specifically Renmin Hospital of Wuhan University (WHRH) and Jinling Hospital in Nanjing (NJJLH) (**Figure** **1**F).

After applying predefined inclusion and exclusion criteria, as described in the Supplementary Methods, a total of 1580 eligible LAGC patients were identified and categorized into three analytical cohorts. The training cohort consisted of 770 patients treated at FHHMU, referred to as Center 1, during the period from 2014 to 2017. The internal validation cohort included 362 patients, who were further divided into two temporal subgroups: internal validation set I involved 194 patients treated between 2012 and 2013, while internal validation set II comprised 168 patients treated from 2018 to 2019.

The remaining cases were assigned to external validation cohorts based on geographic origin. External validation set I included 257 patients from northern institutions (SJZPH, BDCH, and HSPH), and external validation set II included 191 patients from the southern centers (WHRH and NJJLH). To further validate the clinical applicability of the proposed model, a retrospective analysis was conducted using data from a multicenter, prospective clinical trial (NCT 02555358), involving 257 LAGC patients rigorously selected based on defined eligibility criteria, including 93 patients who underwent primary surgery and 164 who received neoadjuvant chemotherapy. For surviving patients, the median follow‐up duration was 70.8 months (interquartile range: 59.4–81.6 months). The early postoperative recurrence rates were 53.7% in the surgery‐only group, 44.0% in the XELOX group, and 37.0% in the DOX group. Further details regarding the study design, inclusion criteria, and treatment allocation can be found in the published protocol (http://links.lww.com/JS9/A933).

In addition, tumor samples from 133 LAGC patients were prospectively collected for RNA sequencing to explore transcriptomic signatures associated with recurrence. Enrichment analyses were subsequently conducted to uncover biological pathways linked to the predictive features. Detailed protocols for specimen handling and transcriptomic analysis are provided in the Supplementary Methods (Supporting Information).

All research procedures adhered to the principles of the Declaration of Helsinki and received ethical approval from the institutional review boards of all participating centers. While informed consent was waived for the retrospective components, written consent was obtained from patients who contributed tumor specimens for transcriptomic analysis.

### 2.2 Clinical Outcomes

The primary outcome of this study was early postoperative recurrence, defined as any recurrence occurring within 24 months after curative surgery and confirmed by clinical symptoms, imaging, or surgical findings.^[^ ^4^, ^5^ ^]^ Overall survival (OS), measured from the date of surgery to death from any cause, was the secondary outcome. By the end of follow‐up in June 2024, the median observation period was 69 months (range 58–165 months). Additional details on follow‐up protocols are provided in the Supplementary Methods (Supporting Information).

### 2.3 Image Acquisition and Segmentation

All patients underwent contrast‐enhanced abdominal CT within two weeks before surgery. Portal venous phase images were retrieved from the institutional picture archiving and communication system (PACS). Tumor segmentation was performed in 3D Slicer, selecting the axial slice with the largest tumor cross‐section as the region of interest (ROI). Two radiation oncologists independently delineated the ROI, and consistency was assessed by a senior expert. Detailed protocols for CT acquisition and segmentation are described in the Supplementary Methods (Supporting Information). Hematoxylin‐eosin staining (H&E‐stained) whole‐slide images were also acquired from formalin‐fixed paraffin‐embedded tumor sections (Figure 1A).

### 2.4 Radiomics Feature Extraction and Model Construction

Tumor segmentation and radiomics feature extraction were performed using 3D Slicer (version 4.10.2), yielding a total of 1,130 standardized features per ROI. After filtering by intra‐ and inter‐observer intraclass correlation coefficient (ICC) (>0.75), univariate analysis and LASSO regression was applied to select 6 predictive features (Figure S1A,B, Supporting Information). Ten classifiers were evaluated on a training cohort (n = 770) and an internal validation cohort (n = 362). Logistic regression with bidirectional stepwise selection achieved the best performance (Training AUC = 0.822, 95% CI: 0.793–0.852; Validation AUC = 0.791, 95% CI: 0.727–0.855), and was selected for model construction (Figure S2A,B and Table S1, Supporting Information). Details of selected features are provided in Table S2 (Supporting Information).

### 2.5 Pathomic Feature Extraction and Model Construction

H&E‐stained whole‐slide images (WSIs) were digitized and tiled into 224×224‐pixel patches. A ResNet18 classifier was trained to recognize eight tumor microenvironment (TME) components and achieved the highest external test accuracy (97.79%) on the HMU‐GC‐HE‐30K dataset (Figure 1B; Table S3, Supporting Information). Patch‐level features were extracted and aggregated to form a 512‐dimensional feature vector per WSI. After ICC filtering and univariate analysis, 182 features remained. LASSO regression further selected 21 features (Figure S1C,D, Supporting Information). Among ten classifiers evaluated, logistic regression again showed superior performance (Training AUC = 0.820, 95% CI: 0.790–0.849; Validation AUC = 0.792, 95% CI: 0.728–0.856) (Figure S2C,D and Table S4, Supporting Information).

### 2.6 Model Development and Evaluation

To predict early postoperative recurrence in patients with LAGC, a multistep modeling framework integrating clinical, radiomic, and pathomic data was developed. Univariate logistic regression was first conducted to assess the association between clinical variables and early recurrence in the training cohort. Significant variables were subsequently included in a multivariate logistic regression model to establish the clinical prediction model. Radiomic and pathomic features were independently selected and modeled using bidirectional stepwise multivariate logistic regression, generating modality‐specific signature scores (Rad‐score and Path‐score). For each modality, the optimal cutoff value was determined using the Youden index, and patients were assigned binary values (0 for low risk, 1 for high risk).

The final Recurrence Stratification Assessment (RSA) model was constructed using multivariate logistic regression by integrating three independent sources of information: 1) clinical predictors, 2) the dichotomized Rad‐score, and 3) the dichotomized Path‐score. This score‐level fusion approach enabled effective integration of multimodal features while maintaining model interpretability. The RSA‐derived recurrence risk scores were further stratified into high‐ and low‐risk groups based on the optimal threshold identified in the training cohort using the Youden index.

Model performance was evaluated using AUC, accuracy, sensitivity, specificity, positive predictive value (PPV), negative predictive value (NPV), positive likelihood ratio (PLR), negative likelihood ratio (NLR), and F1‐score. The added predictive value of the RSA model was quantified using net reclassification improvement and integrated discrimination improvement. Delong's test was applied for AUC comparison across models. Additionally, prediction error curves and the integrated Brier score were used to evaluate overall model performance.

### 2.7 Assessment of Prognosis Prediction and Postoperative Adjuvant Chemotherapy Benefit

To evaluate the predictive utility of the RSA model in guiding adjuvant chemotherapy (AC) decisions, 1:1 propensity score matching was conducted in stage II–III patients based on baseline characteristics, thereby minimizing treatment‐selection bias and confounding due to non‐randomized interventions. Using maximally selected rank statistics within this balanced cohort, a statistically optimal recurrence risk threshold was identified that stratified patients into distinct low‐ and high‐risk groups. Kaplan–Meier analysis demonstrated significant survival differences between these groups, assessed by the log‐rank test. To identify factors associated with overall survival (OS) in LAGC patients, Cox proportional hazards regression was performed across independent validation datasets. To explore the relationship between the RSA model‐predicted recurrence probability and OS in patients who did or did not receive AC, restricted cubic spline (RCS) modeling was performed using the rms package in R. As no distinct inflection point was identified in the RCS curves, the optimal cutoff value was determined using the maximally selected rank statistics method (via the maxstat package). The threshold was consistently identified as 0.19 across the training, internal validation, and external validation cohorts, representing the recurrence risk score above which patients were more likely to benefit from AC (Figure S3, Supporting Information). Additional subgroup analyses were also conducted to validate the robustness of this threshold.

### 2.8 Biological Characteristics and Immune Infiltration

A total of 133 tissue samples were prospectively collected and subjected to RNA sequencing for exploratory analyses (Figure 1E). Functional enrichment analysis was performed to investigate the biological roles of the identified features. Immune infiltration was assessed using CIBERSORTx to estimate the relative abundance of immune cell subsets based on RNA transcript data. In addition, the EcoTyper framework was employed to infer cell types, cell states, and multicellular ecosystems. Detailed procedures for RNA processing, sequencing, and data analysis are provided in the Supplementary Methods (Supporting Information).

### 2.9 Statistical Analysis

All statistical analyses were performed using SPSS version 28.0 (IBM Corp.) and R version 4.3.3 (http://www.r‐project.org). Continuous variables were analyzed using unpaired two‐tailed t‐tests or Mann–Whitney U tests, as appropriate. Categorical variables were compared using the chi‐square (*X* ^2^) test or Fisher's exact test. Univariate and multivariate Cox proportional hazards regression analyses were conducted to evaluate the prognostic significance of variables for survival outcomes. Detailed descriptions of the statistical algorithms are provided in the Supplementary Methods (Supporting Information). A two‐sided P‐value < 0.05 was considered statistically significant.

### 2.10 Ethics Approval and Consent to Participate

The study protocol was approved by the Ethics Committee of the Fourth Hospital of Hebei Medical University (approval number: 2024KY199), and informed consent was obtained from all the study participants. All authors followed applicable ethical standards to maintain research integrity without duplication, fraud, or plagiarism.

### 2.11 Ethical Statement

All authors certify that they comply with the ethical guidelines for authorship.

### 2.12 Data and Code Availability

The core implementation pipeline for the RSA model, including data preprocessing, feature selection, model training, evaluation, and visualization scripts, has been made publicly available at https://github.com/qsyys/GC‐Recurrence.git.

## 3 Results

### 3.1 Patient Characteristics

A total of 1580 patients with LAGC from six medical centers were included and stratified into training (n = 770), internal validation (n = 362), and external validation (n = 448) cohorts. Baseline characteristics were comparable among the three cohorts, with no significant differences in sex, age distribution, ECOG performance status, tumor stage, histology, or AC exposure (all P > 0.05; Table S5, Supporting Information). The population was predominantly male (65.6%) and had a median age of 59 years. Most tumors originated in the lower third of the stomach (52.5%) and were classified as pT4 (75. 4%) and pN+ (72.0%). The majority of patients (82.5%) received 5‐fluorouracil–based AC.

Recurrence occurred in 843 patients (53.4%) overall, with similar recurrence rates across the three cohorts. Compared with non‐recurrent cases, recurrent patients more frequently exhibited advanced pT and pN stages, and poorly differentiated histology. These differences were consistent in the training, internal validation, and external validation sets (all *P* < 0.001; Table S6, Supporting Information).

### 3.2 Development and Validation of the RSA Model

**Figure 2:**
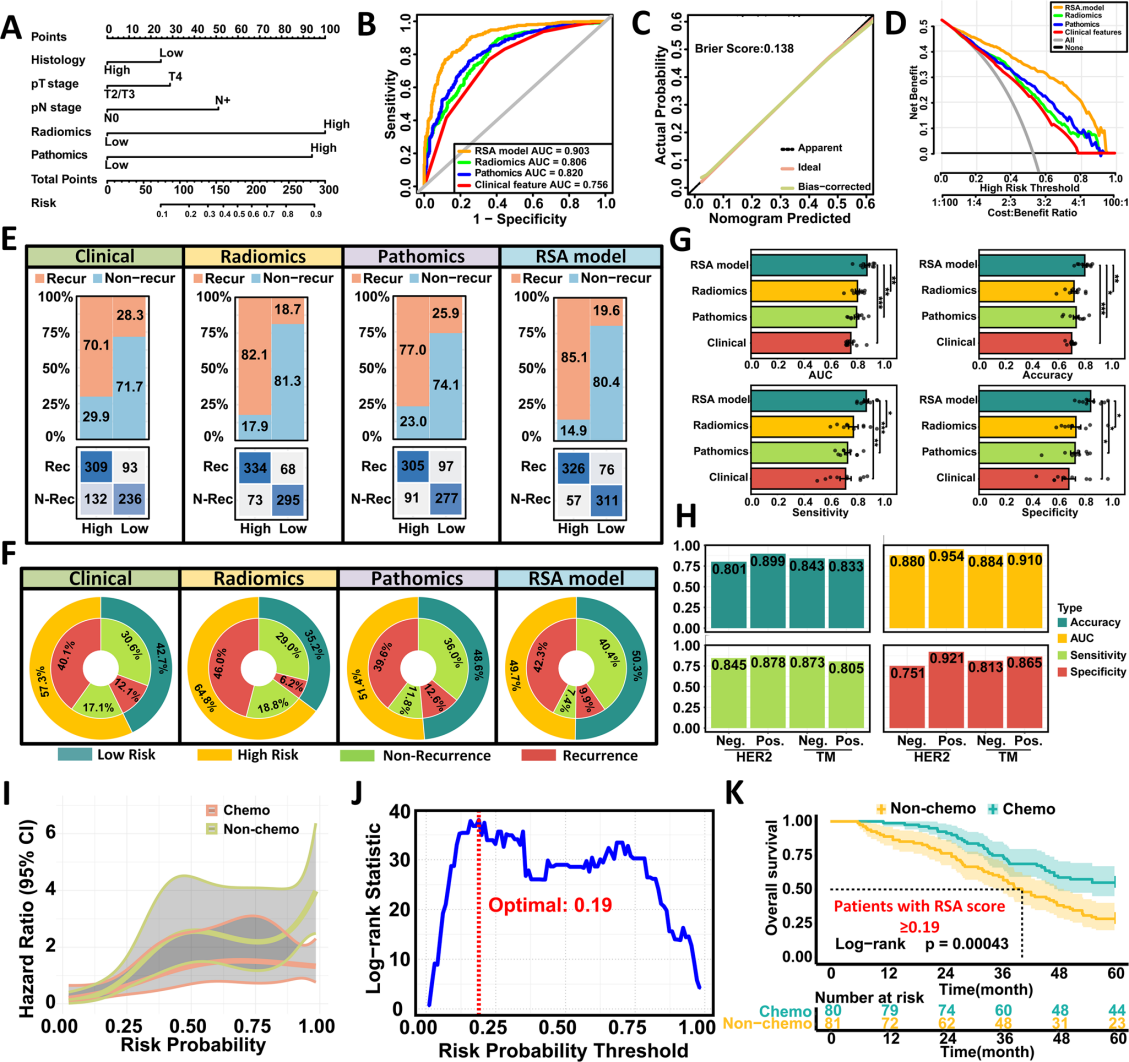
Development and evaluation of the RSA model for predicting early recurrence in LAGC patients. A) Nomogram integrating histological subtype, pT stage, pN stage, radiomic, and pathomic scores to build the final RSA prediction model. B) ROC curves demonstrating that the RSA model achieved superior discrimination (AUC = 0.903) compared to radiomics‐only (AUC = 0.806), pathomics‐only (AUC = 0.820), and clinical‐only (AUC = 0.756) models in the training cohort. C) Calibration curve showing good agreement between predicted and observed recurrence probabilities for the RSA model (Brier score = 0.138). D) Decision curve analysis illustrating that the RSA model offers greater net clinical benefit across a range of threshold probabilities. E) Confusion matrices comparing the classification results of recurrence and non‐recurrence across high‐ and low‐risk groups for each model. The RSA model correctly classified 326 recurrence cases as high‐risk and 311 non‐recurrence cases as low‐risk, demonstrating superior overall classification accuracy. F) Double‐layer concentric circle plots displaying classification performance of each model. The RSA model demonstrated more balanced and accurate identification of recurrence and non‐recurrence cases. G) Tenfold cross‐validation showing that the RSA model consistently achieved higher AUC, accuracy, sensitivity, and specificity compared to other models. H) Model performance stratified by peripheral blood tumor markers (CEA, CA72‐4, CA19‐9) and HER2 expression. The RSA model maintained robust predictive ability across biomarker‐defined subgroups. I) RCS analysis showing the relationship between RSA‐predicted recurrence probability and hazard ratio for overall survival, stratified by receipt of AC. J) Optimal risk threshold (0.19) for AC benefit determined using maximally selected log‐rank statistics. K) Kaplan–Meier curves demonstrating that patients with RSA scores ≥ 0.19 derived significant survival benefit from AC (log‐rank *P* = 0.00043), whereas those without AC had worse 5‐year OS.

Multivariable logistic regression identified pathological subtype, depth of invasion (pT stage), lymph node metastasis (pN stage), radiomic features, and pathomic features as independent predictors of early postoperative recurrence in patients with LAGC (Table S7, Supporting Information). These variables were incorporated into an integrated nomogram referred to as the RSA model (**Figure** **2**A; Figure S4, Supporting Information), which achieved excellent predictive performance in the training cohort with an AUC of 0.903 (95% CI: 0.882 to 0.924) (Figure 2B,E; Figure S5A,B, Supporting Information). Comparative performance across models is detailed in Table S8 (Supporting Information).

Relative to single‐modality models, the RSA model demonstrated significant improvements in both net reclassification improvement (NRI = 0.492, 95% CI: 0.365 to 0.621, *P* < 0.001) and integrated discrimination improvement (IDI = 0.294, 95% CI: 0.261 to 0.390, *P* < 0.001). Similar gains were observed over radiomic‐only and pathomic‐only models (Table S9, Supporting Information). DeLong's test confirmed that the RSA model significantly outperformed the clinical model (AUC 0.903 vs 0.756, *P* < 0.001). Calibration analysis showed good agreement between predicted and observed recurrence rates (Figure 2C).

Based on the Youden index, patients were stratified into low‐ and high‐risk groups. The RSA model classified a greater proportion of patients into the low‐risk category compared to the clinical model (50.3% vs 42.7%) (Figure 2F). Decision curve analysis supported the superior net benefit of the RSA model (Figure 2D). In survival analysis, the 5‐year OS was significantly lower in the high‐risk group than in the low‐risk group (41.5% vs 57.1%, *P* <0.001) (Figure S5C, Supporting Information). Adjusted Cox regression confirmed the RSA model as an independent prognostic factor for 5‐year OS (Table S10, Supporting Information).

Ten‐fold cross‐validation demonstrated better performance of the RSA model over clinical, radiomic, or pathomic models alone in terms of discrimination, sensitivity, and specificity (Figure 2G). Subgroup analyses stratified by peripheral blood markers (carcinoembryonic antigen [CEA], carbohydrate antigen 72‐4 [CA72‐4], and carbohydrate antigen 19‐9 [CA19‐9]) and molecular features such as HER2 and PD‐L1 expression showed that the RSA model consistently outperformed single‐modality models (Figure 2H; Figure S6 and Table S11, Supporting Information), and high‐risk patients had consistently worse 5‐year OS across subgroups (Figure S7, Supporting Information).

Finally, we explored the association between RSA‐predicted recurrence probability and AC benefit among patients with stage II and III disease. Restricted cubic spline modeling suggested greater benefit from AC with increasing predicted risk (Figure 2I). As no clear inflection point was observed, the optimal threshold for stratifying AC benefit was determined to be 0.19 using maximally selected rank statistics in a propensity score‐matched cohort (Figure 2J). In the matched cohort of 216 patients (Table S12, Supporting Information), AC conferred no significant survival benefit among those with RSA scores below 0.19 (67.9% vs 59.3%, *P* = 0.51, Figure S8, Supporting Information). In contrast, patients with scores of 0.19 or higher benefited significantly from AC (55.0% vs 28.4%, *P* < 0.001, Figure 2k), and multivariable Cox regression confirmed AC as an independent prognostic factor in this group (HR = 2.087, 95% CI: 1.375 to 3.166, *P* = 0.001) (Table S13, Supporting Information).

### 3.3 Internal Validation of the RSA Predictive Model

**Figure 3:**
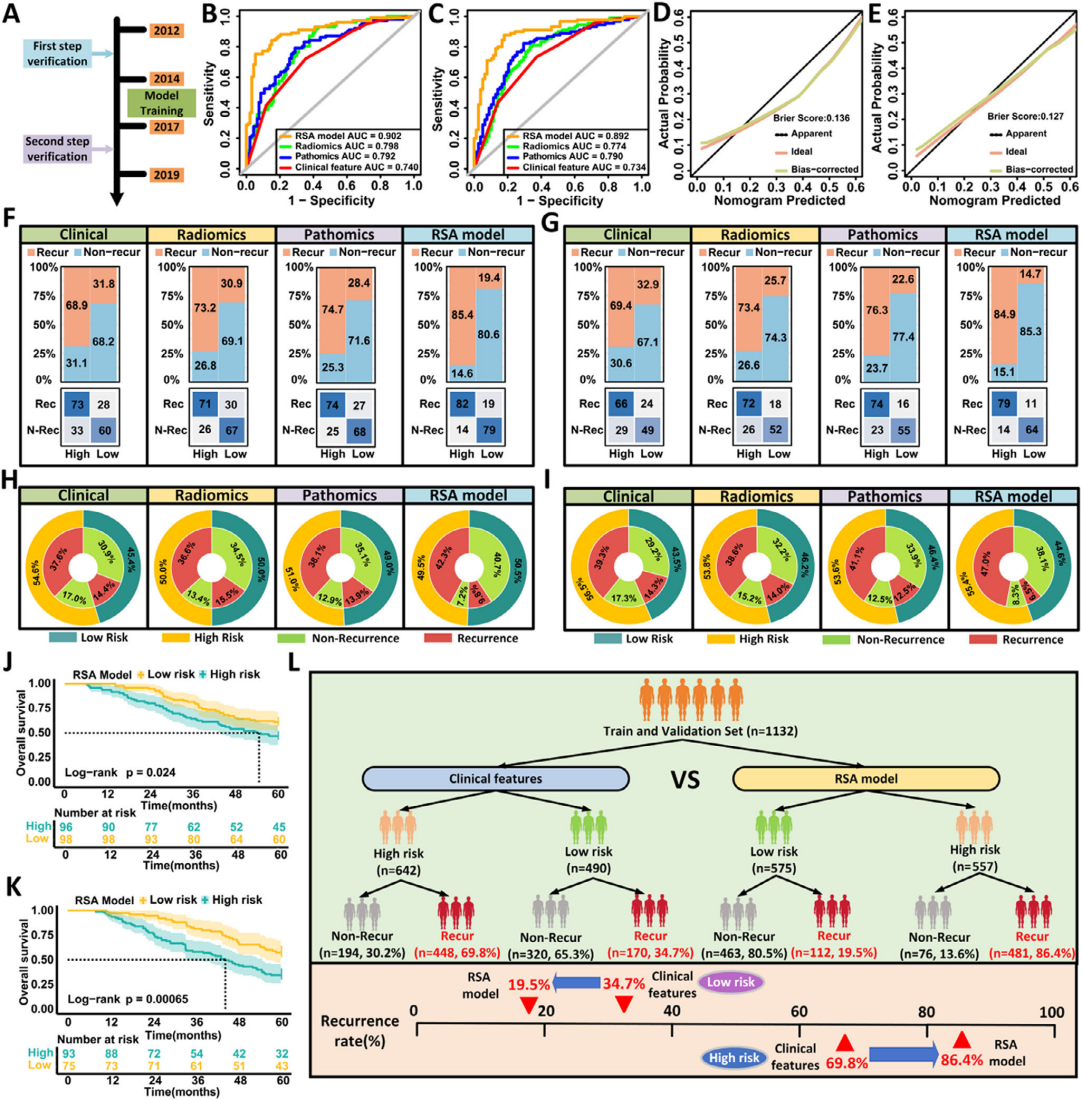
Internal validation of the RSA model for predicting early recurrence in patients with LAGC. A) Patient allocation schema showing internal validation cohorts from different time periods: Internal Validation Set I (2012–2014, n = 194) and Internal Validation Set II (2017–2019, n = 168). B,C) ROC curves comparing the predictive performance of clinical‐only, radiomics‐only, pathomics‐only, and RSA models in Internal Validation Set I (B) and Set II (C). The RSA model achieved the highest AUC in both subsets (0.902 and 0.892, respectively). D,E) Calibration curves for the RSA model in Set I (D, Brier score = 0.136) and Set II (E, Brier score = 0.127), demonstrating good agreement between predicted and observed recurrence probabilities. F,G) Confusion matrices showing classification results of high‐ and low‐risk groups across different models in Set I (F) and Set II (G). The RSA model achieved the highest correct classification rate for both recurrent and non‐recurrent patients. H,I) Double‐layer concentric circle plots illustrating the consistency between predicted risk and actual recurrence status in Set I (H) and Set II (I). The RSA model displayed improved sensitivity and negative predictive value compared to other models. J,K) Kaplan–Meier survival curves comparing 5‐year overall survival between RSA‐defined high‐ and low‐risk groups in Set I (J) and Set II (K). The RSA model effectively stratified patients with significantly different survival outcomes in both cohorts (log‐rank P = 0.024 and P = 0.00065, respectively). L) Comparative analysis of recurrence stratification based on clinical features versus RSA model in the combined training and internal validation cohorts (n = 1132). The RSA model reclassified more patients into appropriate risk categories, identifying 86.4% of recurrent cases in the high‐risk group compared to 69.8% by the clinical model, and reduced the recurrence rate among predicted low‐risk patients from 34.7% to 19.5%.

To validate the predictive performance of the RSA model, we retrospectively analyzed 362 patients with locally advanced gastric cancer from FHHMU, divided into two subsets based on enrollment period: internal validation set I (January 2012 to January 2014, n = 194) and internal validation set II (January 2017 to January 2019, n = 168) (**Figure** **3**A). Baseline characteristics were comparable between the two sets (Table S14, Supporting Information, all *P* > 0.05). In internal validation set I, the RSA model achieved an AUC of 0.902 (95% CI: 0.858 to 0.946), significantly outperforming models based on clinical, radiomic, or pathomic features alone (Figure 3B,F; Figure S9A,B and Table S15, Supporting Information). DeLong's test confirmed statistical superiority over the clinical‐only model (*P* < 0.001; Table S9, Supporting Information), with notable improvement in both reclassification (NRI = 0.662, 95% CI: 0.391 to 0.934; *P* < 0.001) and discrimination (IDI = 0.318, 95% CI: 0.255 to 0.378; *P* < 0.001). Calibration analysis supported its predictive accuracy (Brier score = 0.136; Figure 3D). Similar performance was observed in validation set II (Figure 3C,E,G; Figure S9D,E and Table S15, Supporting Information).

The RSA model stratified a greater proportion of early recurrence cases within the high‐risk group compared to the clinical model in both sets (42.3% vs 37.6% in set I, Figure 3H; 47.0% vs 39.3% in set II, Figure 3I). Decision curve analysis demonstrated improved clinical utility (Figure S9C,F, Supporting Information), and sensitivity analysis showed a higher identification rate of recurrence in high‐risk patients (86.4% vs 69.8%), reducing false‐negative classifications by 15.2% compared to the clinical model (Figure 3L). Kaplan–Meier analysis showed significantly better 5‐year overall survival in low‐risk patients compared to high‐risk counterparts (set I: 61.2% vs 46.9%, P = 0.024; set II: 57.3% vs 34.4%, P = 0.00065; Figure 3J,K). The RSA model remained an independent prognostic factor in multivariable Cox regression (Tables S16 and S17, Supporting Information).

We further explored the predictive value of RSA for adjuvant chemotherapy benefit in stage II and III patients. Among 92 patients matched by propensity scores (Table S18, Supporting Information), survival benefit was evident only in those with RSA‐predicted recurrence probabilities ≥0.19 (68.6% vs 33.3%, *P* = 0.0036;  ; Figure S10, Supporting Information). Cox regression confirmed chemotherapy as an independent protective factor in this subgroup (HR = 2.390, 95% CI: 1.271 to 4.492; *P* = 0.007; Table S19, Supporting Information).

### 3.4 Independent Multicenter Validation of the RSA Model

**Figure 4:**
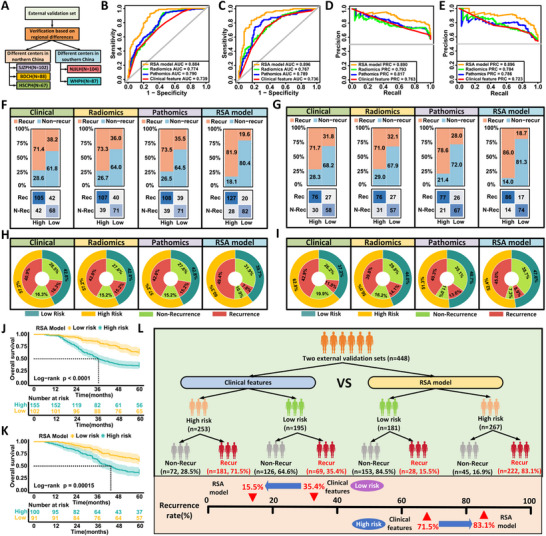
External validation of the RSA model in two independent multicenter cohorts. A) Patient stratification for external validation based on geographic regions: External Validation Set I (n = 257 from northern centers: SJZPH, BDCH, HSCPH) and Set II (n = 191 from southern centers: NJJLH, WHRH). B,C) ROC curves comparing clinical‐only, radiomics‐only, pathomics‐only, and RSA models in Set I (B) and Set II (C). The RSA model achieved the highest AUC in both cohorts (0.884 and 0.896, respectively). D,E) Precision–recall curves evaluating predictive performance across models in Set I (D) and Set II (E), further confirming the superior precision of the RSA model. F,G) Confusion matrices comparing classification outcomes of recurrence versus non‐recurrence under high‐ and low‐risk predictions by each model in Set I (F) and Set II (G). The RSA model showed the best sensitivity and negative predictive value. H,I) Double‐layer concentric circle plots illustrating the classification consistency of each model in Set I (H) and Set II (I). The RSA model provided a more balanced separation of high‐ and low‐risk patients. J,K) Kaplan–Meier survival curves comparing 5‐year overall survival between RSA‐defined high‐ and low‐risk groups. In both sets, the RSA model stratified patients into significantly distinct prognostic groups (Set I: *P* < 0.0001; Set II: *P* = 0.00015). L) Summary comparison of recurrence stratification by clinical model versus RSA model in the combined external validation cohort (n = 448). The RSA model reclassified more patients appropriately, identifying 83.1% of recurrent cases in the high‐risk group compared to 71.5% with the clinical model, and reduced misclassified recurrence in the low‐risk group from 35.4% to 15.5%.

To assess the generalizability of the RSA model, we performed external validation in two independent cohorts. External Validation Set I included 257 patients from three northern Chinese centers (SJZPH, BDCH, HSCPH), and Set II comprised 191 patients from two southern centers (WHRH, NJJLH) (**Figure** **4**A). Baseline characteristics are summarized in Table S20 (Supporting Information).

In External Validation Set I, the RSA model achieved an AUC of 0.884 (95% CI: 0.842–0.926), outperforming clinical‐, radiomic‐, and pathomic‐only models (Figure 4B,D,F; Table S21, Supporting Information). Compared with the clinical model, it showed significant improvements in NRI (0.622, 95% CI: 0.349–0.913; *P* < 0.001) and IDI (0.275, 95% CI: 0.215–0.336; *P* < 0.001; Table S9, Supporting Information), with good calibration (Brier score = 0.123; Figure S11A,B, Supporting Information). Similar performance was observed in External Validation Set II (Figure 4C,E,G; Figure S11D,E and Table S22, Supporting Information).

Decision curve analysis demonstrated greater net clinical benefit of the RSA model in both cohorts (Figure S11C,F, Supporting Information), and concentric circle plots confirmed improved high‐risk identification over the clinical model (Figure 4H,I). A combined sensitivity analysis showed the RSA model detected 11.6% more high‐risk patients while reducing low‐risk misclassification by 19.9% (Figure 4L).

Survival analysis revealed significantly lower 5‐year OS in high‐risk versus low‐risk patients (Set I: 36.1% vs 63.7%, P < 0.001; Set II: 37.0% vs 62.6%, *P* < 0.001; Figure 4J,K). Multivariate Cox regression confirmed the RSA model as an independent prognostic factor in both cohorts (Tables S22 and S23, Supporting Information). Furthermore, consistent with prior findings, only patients with RSA‐predicted risk ≥ 0.19 derived survival benefit from AC (66.0% vs 24.5%, P < 0.001; Figure S12, and Tables S24 and S25, Supporting Information).

### 3.5 Real‐World Validation of the RSA Model Using Prospective Trial Data

**Figure 5:**
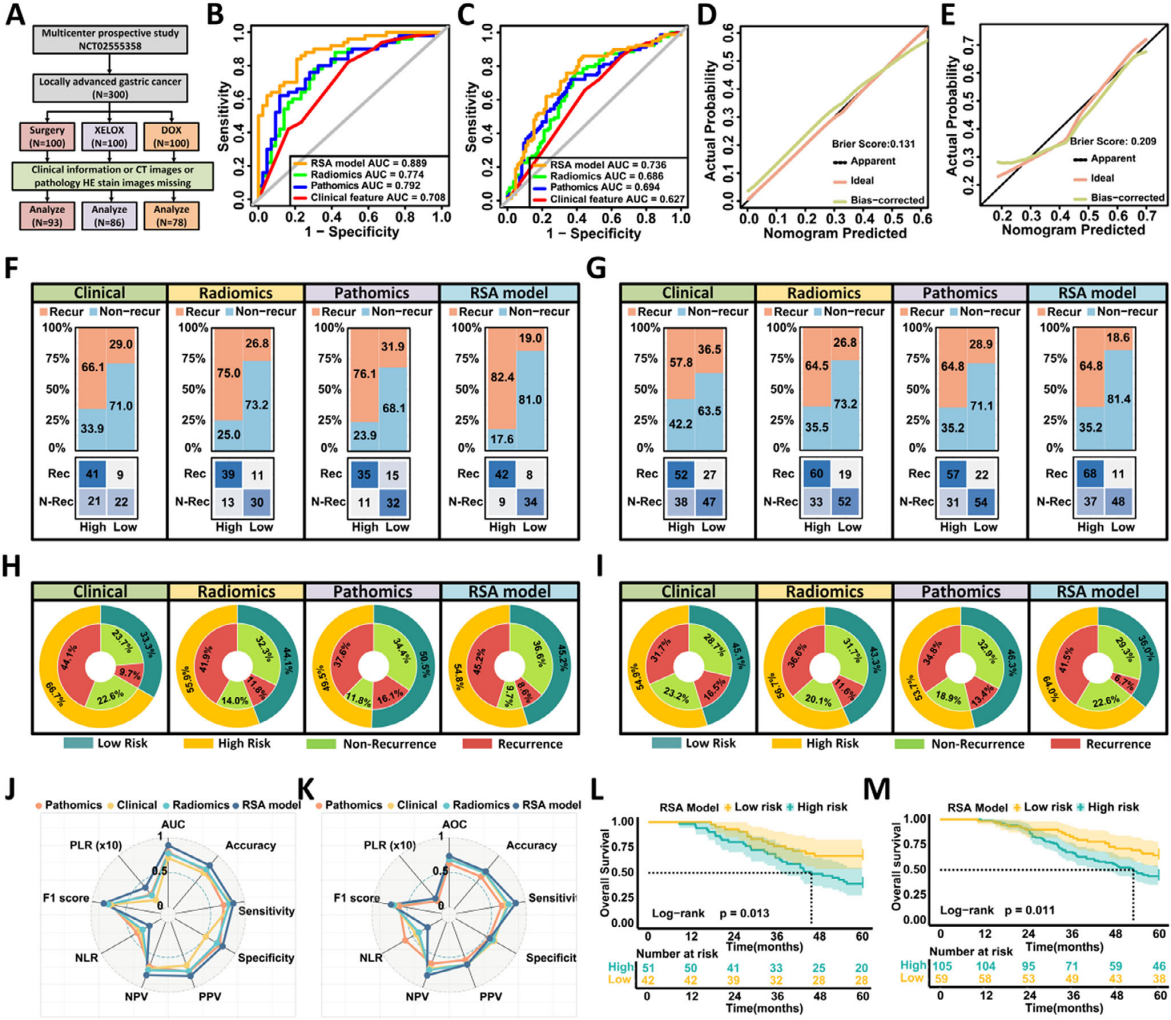
Retrospective analysis of the RSA model using data from a multicenter prospective trial cohort. A) Patient selection flowchart from the NCT02555358 prospective trial, including 93 patients undergoing surgery alone, 86 receiving neoadjuvant XELOX, and 78 receiving neoadjuvant DOX. B,C) ROC curves comparing clinical, radiomics, pathomics, and RSA models in the surgery‐only subgroup (B) and neoadjuvant chemotherapy subgroup (C). The RSA model achieved high predictive accuracy in surgery‐only patients (AUC = 0.889), but showed limited discrimination after neoadjuvant chemotherapy (AUC = 0.736). D,E) Calibration curves for the RSA model in the surgery‐only (D) and neoadjuvant chemotherapy (E) groups, demonstrating acceptable agreement between predicted and observed recurrence probabilities (Brier scores = 0.131 and 0.209, respectively). F,G) Confusion matrices showing model‐based classification of recurrence versus non‐recurrence among high‐ and low‐risk groups. The RSA model provided the most accurate classification in both patient subgroups. H,I) Double‐layer concentric circle plots comparing classification consistency across models in surgery‐only (H) and neoadjuvant chemotherapy (I) patients. J,K) Radar plots comparing comprehensive model performance metrics, including AUC, accuracy, sensitivity, specificity, PPV, NPV, PLR, NLR, and F1 score. RSA outperformed all single‐modality models, particularly in the surgery‐only subgroup. L,M) Kaplan–Meier survival curves showing that RSA‐defined high‐risk patients had significantly worse 5‐year overall survival in both subgroups (surgery‐only: *P* = 0.013; neoadjuvant chemotherapy: *P* = 0.011).

To validate the RSA model prospectively, we retrospectively analyzed 257 LAGC patients from a multicenter prospective trial (NCT02555358) after applying strict eligibility criteria. Among them, 93 patients (36.2%) underwent primary surgery, while 86 (33.5%) and 78 (30.3%) received neoadjuvant XELOX or DOX regimens, respectively (**Figure** **5**A). The liver was the most common site of recurrence across all subgroups (Figure S13A, Supporting Information). Baseline characteristics are provided in Table S26 (Supporting Information); detailed clinical outcomes were previously published.^[^ ^26^ ^]^

In the primary surgery subgroup, the RSA model outperformed single‐modality models, achieving an AUC of 0.889 (95% CI: 0.826–0.952; Figure 5B,D–F,J; Figure S13B and Table S27, Supporting Information). It significantly improved risk reclassification (NRI = 0.598; 95% CI: 0.211–0.973; P < 0.001) and discrimination (IDI = 0.302; 95% CI: 0.202–0.398; *P* < 0.001) over the clinical‐only model (Table S9, Supporting Information). Concentric circle plots and decision curve analysis further supported the model's predictive accuracy and clinical utility (Figure 5H; Figure S13C, Supporting Information). Additionally, patients identified as high‐risk by the RSA model had significantly worse five‐year overall survival than those in the low‐risk group (39.2% vs 66.7%, *P* = 0.013; Figure 5L).

In contrast, for patients receiving neoadjuvant chemotherapy, the RSA model showed relatively limited predictive power, with an AUC of 0.736 (95% CI: 0.653–0.811; Figure 5C,G,I; Figure S13D, Supporting Information). Nevertheless, calibration curves and radar plots still supported its superior performance over other models (Figure 5E,K; Figure S13E, and Tables S9 and S27, Supporting Information). A significant survival difference remained between the high‐ and low‐risk groups (43.8% vs 64.4%, *P* = 0.011; Figure 5M), although the RSA score did not clearly correlate with treatment response (Figure S13F,G, Supporting Information).

### 3.6 Refined Prognostic Stratification by the RSA Model Beyond Existing Staging Systems

**Figure 6:**
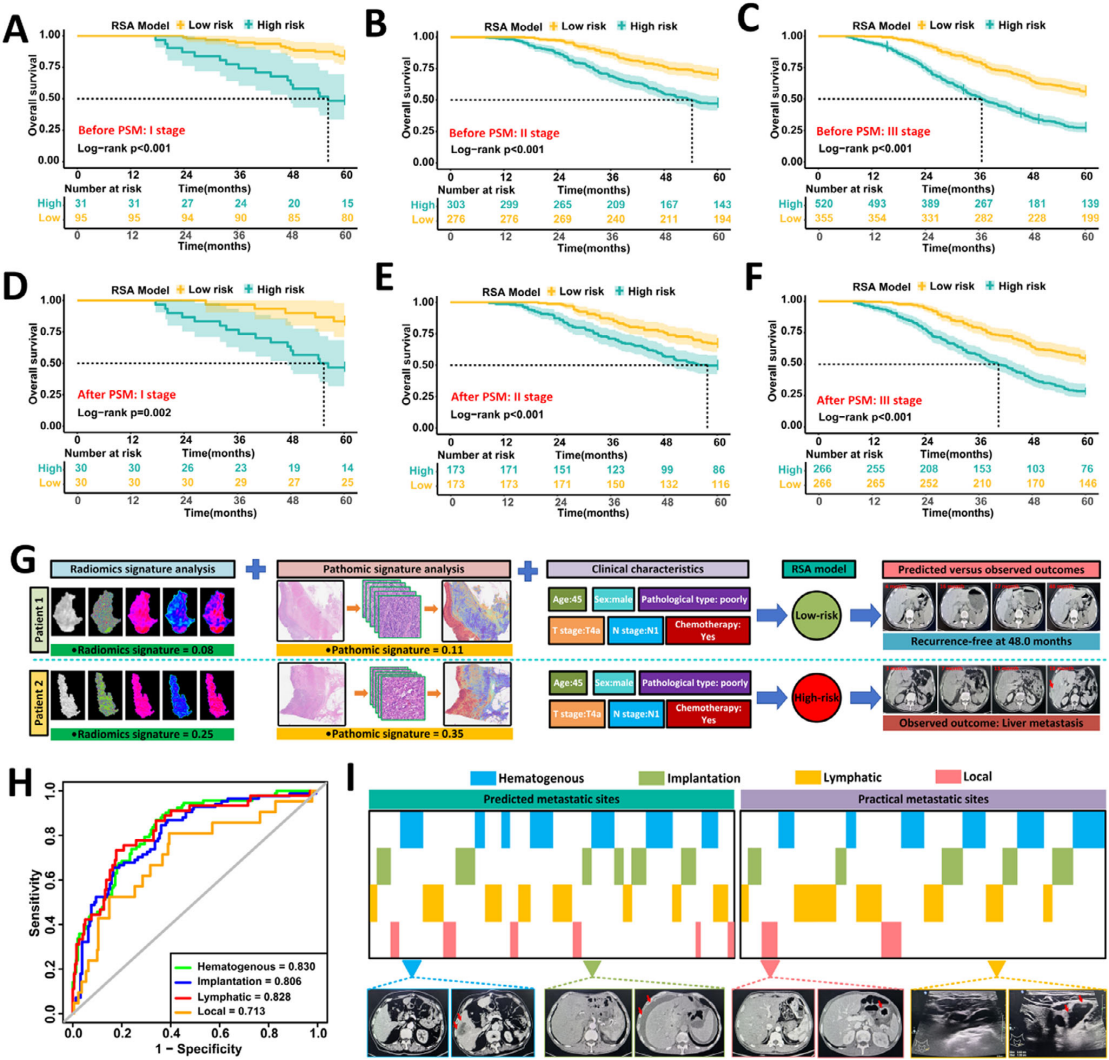
Added prognostic value of the RSA model beyond TNM staging and its potential to predict metastatic patterns. A–C) Kaplan–Meier survival curves comparing overall survival between RSA‐defined high‐ and low‐risk patients within TNM stage I (A), stage II (B), and stage III (C) before propensity score matching (PSM). In all stages, RSA stratification yielded significant prognostic separation (all log‐rank *P* < 0.001). D–F) Kaplan–Meier survival curves after PSM confirming that RSA‐defined risk groups remained significantly associated with prognosis within TNM stage I (D), stage II (E), and stage III (F). G) Case‐level illustration comparing two patients with identical TNM stage and treatment regimens but divergent RSA scores and clinical outcomes. Radiomic and pathomic feature heterogeneity contributed to differential RSA‐predicted risks, corresponding to actual recurrence and survival outcomes. H) ROC curves showing the predictive accuracy of RSA‐based models in identifying distinct postoperative metastatic patterns. The model achieved AUCs of 0.830 for hematogenous, 0.806 for implantation, 0.828 for lymphatic, and 0.713 for local recurrence. I) Comparison of RSA model–predicted versus actual metastasis types in representative patients, with color‐coded mapping of predicted versus observed metastatic sites across hematogenous, implantation, lymphatic, and local spread pathways. CT images highlight examples of site‐specific recurrence.

To assess whether the RSA model offers additional prognostic value beyond conventional TNM staging, we performed risk stratification within each TNM stage. Kaplan–Meier analyses demonstrated that the RSA model effectively separated patients into high‐ and low‐risk groups with significantly different overall survival across stages I to III (all log‐rank *P* < 0.05; Figure S14, Supporting Information). These findings were validated by 1:1 propensity score matching, which consistently showed worse prognosis in high‐risk groups (**Figure** **6**A–F; Table S28, Supporting Information). Multivariable Cox regression further confirmed the RSA model as an independent predictor of 5‐year OS across all TNM subgroups (Tables S29–S31, Supporting Information). To illustrate the RSA model's clinical utility, Figure 6G presents two representative patients with identical TNM stage and treatment regimens but divergent survival outcomes. Figure S15 (Supporting Information) reveals the underlying radiomic and pathomic differences that contributed to this prognostic discrepancy.

We next evaluated the RSA model's ability to enhance molecular subtype–based classification. Among 217 patients from the training cohort, immunohistochemical profiling of eight markers (E‐cadherin, Vimentin, N‐cadherin, MLH1, MSH2, MSH6, PMS2, and P53) allowed classification into four molecular subtypes according to the ACRG framework (Figure S16A, Supporting Information). Within each subtype, the RSA model further stratified patients into distinct prognostic groups with significantly different overall survival (MSI: *P* = 0.032; MSS with EMT: *P* = 0.043; MSS with TP53 loss: *P* = 0.005; MSS with TP53 intact: P = 0.043; Figure S16B–E, Supporting Information).

Finally, we assessed whether the RSA model could distinguish among major postoperative recurrence patterns, including hematogenous, lymphatic, peritoneal, and local invasion. Multivariate logistic regression identified distinct radiomic and pathomic signatures for each recurrence type (Table S32, Supporting Information). Corresponding predictive models achieved high discrimination with area under the curve values exceeding 0.7 (Figure 6H) and demonstrated concordance rates above 75% with observed recurrence patterns (Figure 6I).

### 3.7 External Validation and Tumor Immune Profiling of the RSA Model in the TCIA Cohort

To independently validate the RSA model, we analyzed 41 gastric cancer patients from The Cancer Imaging Archive (TCIA) after applying inclusion criteria (Figure S17A and Table S33, Supporting Information). Of these, 48.8% experienced recurrence. The RSA model outperformed clinical, radiomic, and pathomic single‐feature models in predicting early postoperative recurrence (AUC = 0.898; 95% CI: 0.804–0.992; Figure S17B–E and Table S34, Supporting Information), and achieved significant improvements in NRI (0.357; 95% CI: ‐0.191‐0.905; *P* < 0.001) and IDI (0.269; 95% CI: 0.134–0.405; *P* < 0.001; Table S35, Supporting Information). The calibration curve (Figure S17F, Supporting Information), concentric circle plot (Figure S17G, Supporting Information), decision curve analysis (Figure S17H, Supporting Information), and Kaplan–Meier analysis (Figure S17I, Supporting Information) consistently supported the model's predictive and clinical utility.

**Figure 7:**
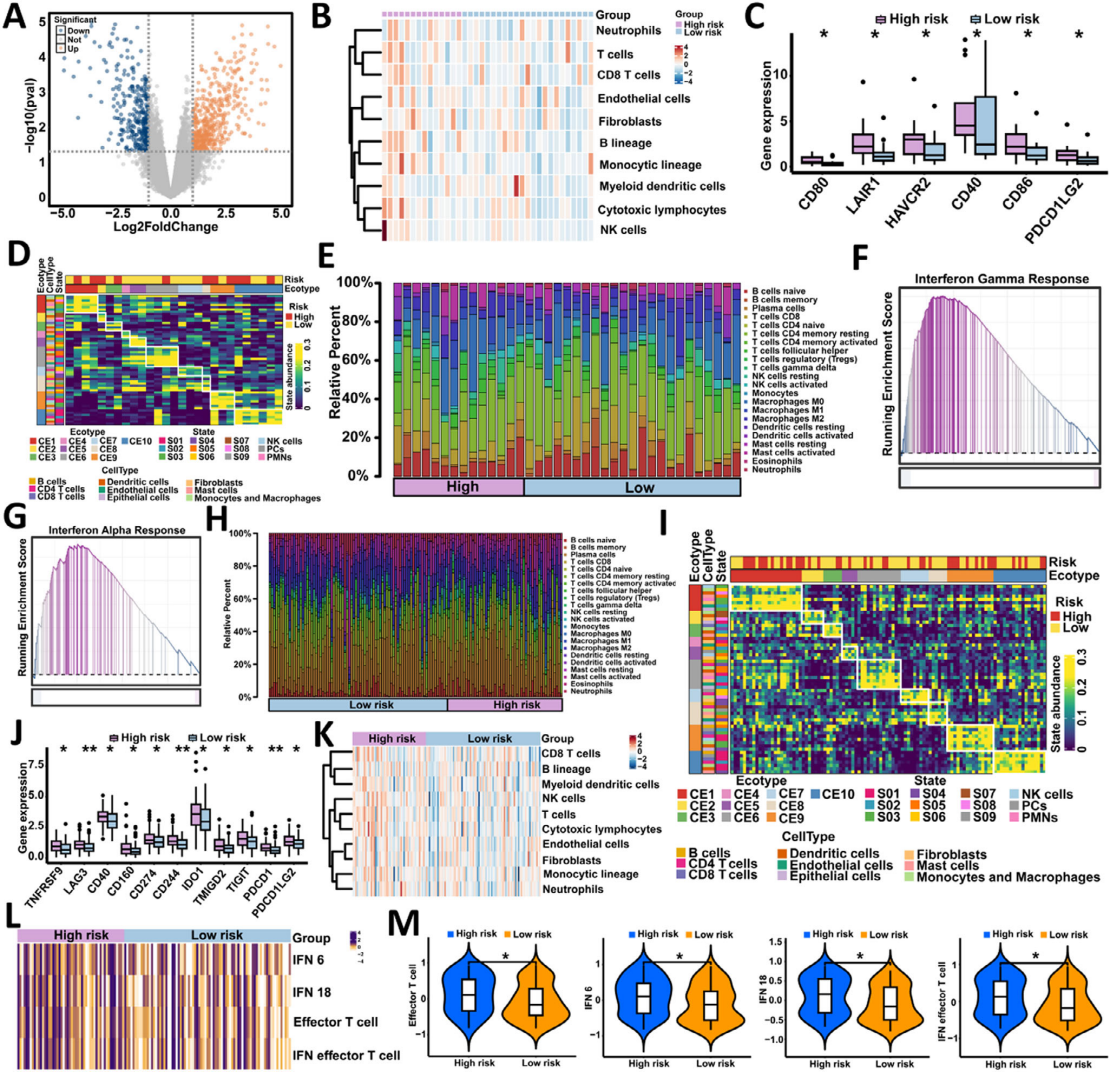
Immune landscape and transcriptomic characterization of RSA‐defined recurrence risk groups in gastric cancer. A) Volcano plot showing differentially expressed genes between RSA‐defined high‐risk and low‐risk groups in the TCIA gastric cancer cohort. B) MCPcounter‐based estimation of immune cell populations in TCIA samples, indicating increased infiltration of CD8+ T cells, NK cells, and myeloid cells in the high‐risk group. C) Boxplots showing significantly elevated expression of immune checkpoint genes (CD80, HAVCR2, CD40, CD86, and PDCD1LG2) in high‐risk patients from the TCIA cohort. D) Heatmap depicting the distribution of cell states and ecosystems based on EcoTyper analysis in the TCIA cohort, with enrichment of CE8 and CE9 ecosystems in high‐risk tumors. E) Bar plot of relative immune cell proportions estimated by CIBERSORT in the TCIA cohort, showing distinct immune compositions between risk groups. F) Gene set enrichment analysis (GSEA) showing activation of the interferon gamma response pathway in the high‐risk group. G) GSEA revealing upregulation of the interferon alpha response pathway in high‐risk tumors. H) CIBERSORT‐derived immune cell abundance heatmap in an independent RNA sequencing cohort, validating increased immune infiltration in high‐risk patients. I) EcoTyper‐based classification of cell states and ecosystems in the sequencing cohort, highlighting consistent enrichment of immune‐exhausted ecosystems in the high‐risk group. J) Boxplots showing increased expression of immune checkpoint molecules in high‐risk tumors from the RNA‐seq cohort, consistent with findings from the TCIA dataset. K) MCPcounter‐based immune cell composition in the sequencing cohort, confirming elevated levels of effector immune cells in the high‐risk group. L) Heatmap showing enhanced expression of gene signatures related to interferon signaling and effector T cell function in high‐risk tumors. M) Violin plots displaying significantly higher IFNγ scores, T cell effector activity, and cytotoxicity in high‐risk tumors, indicating elevated immune activation and potential response to immunotherapy. (ns = not significant; ^*^ *P* < 0.05; ^**^ *P* < 0.01).

To explore the biological relevance of RSA‐based risk stratification, we analyzed transcriptomic data from the same cohort. Differential expression analysis revealed distinct immune profiles between high‐ and low‐risk patients (**Figure** **7**A). High‐risk tumors exhibited increased infiltration of immune cells as estimated by CIBERSORT and MCPcounter (Figure 7B,E), elevated stromal and immune scores (Figure S18B, Supporting Information), and higher expression of T cell‐related immune checkpoints such as CD80, CD40, and CD86 (Figure 7C; Figure S19A, Supporting Information). Gene set enrichment analysis (GSEA) showed enrichment of interferon‐α and interferon‐γ signaling in the high‐risk group (Figure 7F,G). Multicellular community mapping further revealed significantly higher abundance of CE8 and CE9 in high‐risk patients (Figure 7D; Figure S19B, Supporting Information).

These findings were corroborated by an extended RNA‐seq analysis using GSVA and GSEA, which identified strong upregulation of immune and inflammatory pathways, including IL6/JAK/STAT3 signaling, interferon responses, and general inflammation, in the high‐risk group (Figure S18A,C, Supporting Information). Stromal, immune, and ESTIMATE scores were significantly elevated (Figure S18D, Supporting Information), while CIBERSORT and MCPcounter again indicated increased CD8+ T cell infiltration (Figure 7H,K). Immune checkpoint expression was markedly upregulated in high‐risk tumors (Figure 7J), and community analysis confirmed enrichment of CE1 and CE9 (Figure 7I; Figure S19C, Supporting Information). Scoring of immune checkpoint‐related gene signatures revealed significantly higher IFNγ‐6, IFNγ‐18, effector T cell, and composite scores in the high‐risk group (Figure 7L,M), suggesting greater potential responsiveness to immune checkpoint blockade therapies.

To explore the biological relevance of radiomic and pathomic features selected in the RSA model, we performed Spearman correlation analysis between imaging‐based feature scores and the expression levels of key immune‐related pathways identified from transcriptomic profiling. As shown in Figure S20 (Supporting Information), radiomic features showed significant positive correlations with immune activation signatures, including Effector T cell (R = 0.43, *P* <0.001) and IFNγ (R = 0.26, *P* = 0.003), while pathomic features were negatively correlated with the same pathways (for example, Effector T cell: R = −0.59, *P* < 0.001), suggesting a strong association between image‐derived scores and underlying immune phenotypes.

## 4 Discussion

This study established and validated a robust, interpretable multimodal fusion model integrating clinical, radiomic, and pathomic features (termed the RSA model) to predict early postoperative recurrence and prognosis in patients with LAGC. The RSA model demonstrated significantly superior predictive performance compared to conventional clinical models or single‐modality radiomic and pathomic approaches, with consistently high accuracy (AUC > 0.88) validated across internal, external, prospective, and public datasets. Moreover, patients stratified as high‐risk by the RSA model exhibited markedly poorer five‐year overall survival, underscoring its strong prognostic relevance. Importantly, subgroup analyses suggested that the RSA model could effectively guide individualized treatment decisions by identifying patients who could benefit most from postoperative adjuvant chemotherapy.

Previous studies have primarily utilized clinicopathological parameters, including TNM staging, histological subtype, and lymph node status, to predict recurrence and prognosis in gastric cancer.^[^ ^27^, ^28^ ^]^ Although these parameters are clinically useful, they are insufficient to fully capture tumor heterogeneity and biological complexity, resulting in limited predictive performance.^[^ ^29^, ^30^ ^]^ Recently, radiomics‐based prediction models have emerged as promising tools due to their non‐invasive, quantitative nature in capturing intratumoral heterogeneity.^[^ ^31^, ^32^, ^33^ ^]^ Cao et al. demonstrated that a radiomics signature based on preoperative CT images could predict postoperative recurrence in gastric cancer, achieving moderate accuracy (AUC = 0.833).^[^ ^23^ ^]^ Similarly, Dong et al. validated a radiomic signature predicting lymph node metastasis with an AUC of ≈0.821.^[^ ^34^ ^]^ Despite these advances, radiomics models alone are still limited by their inability to capture histological and biological features that significantly impact tumor behavior.

In recent years, pathomics, an advanced computational pathology approach extracting quantitative morphological and textural features from digital histopathology images, has gained attention in cancer prognosis prediction. Previous research by Chen et al. reported that pathomics could effectively predict recurrence in gastric cancer patients with higher accuracy than traditional pathology assessment alone^[^ ^35^ ^]^. Additionally, Chen et al. developed a histopathological image‐based prediction model to stratify survival in gastric cancer patients, further supporting the predictive value of pathomic features.^[^ ^36^ ^]^ However, the integration of pathomic features with radiomic and clinical features, as conducted in our RSA model, has seldom been thoroughly explored. Our study uniquely integrates these three data modalities, achieving substantial improvements in prediction accuracy compared to previous single‐modality models, highlighting the complementary value of combined radiomic‐pathomic analysis.

In this study, transcriptomic analysis and functional enrichment suggested potential biological underpinnings of our model's predictive capability. We observed significant differences in immune‐related pathways, such as interferon‐α/γ signaling and inflammation‐related IL6/JAK/STAT3 pathway activation, between RSA classified high‐risk and low‐risk patients. This finding aligns with existing literature indicating that an immunosuppressive tumor microenvironment, characterized by increased infiltration of immune‐suppressive cells such as regulatory T cells and tumor‐associated macrophages, as well as activation of immune checkpoint pathways, significantly contributes to the recurrence risk in gastric cancer.^[^ ^37^, ^38^ ^]^ Our RSA‐derived signature notably distinguished patients based on immune infiltration and checkpoint expression, suggesting a robust biological basis underpinning the radiomic‐pathomic characteristics that our model captured. To improve clinical interpretability, we further visualized how radiomic and pathomic features jointly influenced recurrence predictions using SHAP‐based methods. For example, radiomic heterogeneity combined with stromal‐rich histology increased predicted risk in high‐risk patients. These visual tools help clinicians understand not only which features matter, but how they interact to shape individualized risk profiles. These findings reinforce the potential clinical utility of RSA risk stratification, possibly guiding immunotherapy or immune checkpoint blockade in high‐risk LAGC patients.

Despite the promising performance and extensive validation, our study has several limitations. First, it was retrospectively designed, and despite stringent inclusion and exclusion criteria, potential selection bias and confounding variables cannot be completely excluded. Second, while the RSA model underwent robust validation across multiple institutions, the generalizability of our findings to different patient populations or imaging/pathology protocols requires further prospective validation in diverse clinical settings. Although we included a public dataset (TCIA cohort) to preliminarily evaluate model generalizability, the overall study population was still relatively homogeneous in terms of ethnicity and geography. Future international validation involving broader populations and heterogeneous imaging protocols is warranted to support wider clinical applicability. Third, although transcriptomic analysis provided initial mechanistic insights, the specific causal relationships between identified radiomic/pathomic features and biological pathways remain to be elucidated through in‐depth functional experimental validation. Fourth, while the RSA model is built on routinely available data types such as preoperative contrast‐enhanced CT scans and digitized histological slides, it has not yet been translated into a fully automated or clinician‐facing platform. However, the model architecture is modular and compatible with standard DICOM and whole‐slide image formats, which supports future integration into digital clinical workflows. Additionally, our current RSA model may be less effective in predicting recurrence after neoadjuvant chemotherapy, highlighting the need to develop or adapt predictive models specific for patients undergoing preoperative systemic treatments. Future studies should incorporate multi‐omics data, including genomics and proteomics, to further enhance predictive accuracy, refine biological interpretability, and comprehensively personalize treatment strategies.

In conclusion, we developed and validated a novel interpretable multimodal fusion model integrating clinical, radiomic, and pathomic features, which demonstrated superior accuracy and generalizability for predicting postoperative recurrence and survival in patients with locally advanced gastric cancer. Compared with existing clinical‐only or single‐modality models, our RSA model offers substantial improvement in predictive performance, enabling more precise risk stratification, individualized prognosis, and potentially optimized treatment selection. The biological insights gained through transcriptomic analysis further substantiate the clinical applicability of the RSA model, emphasizing its potential utility for guiding individualized clinical decision‐making and personalized therapy in gastric cancer management.

## Conflict of Interest

The authors declare no conflict of interest.

## Author Contributions

P.D., J.Y., S.C., and H.G. contributed equally to this work. Y.L., L.M., and Q.Z. contributed equally as co‐corresponding authors. Q.Z. and L.M. performed conception and design; Q.Z. performed administrative support; P.D., H.G., J.Y., S.C., J.W., H.W., W.M., Y.T., R.G., L.Z., N.M., X.L., Z.G., L.M., and Q.Z. contributed in Provision of study materials or patients; P.D., H.G., J.Y., S.C., J.W., H.W., and W.M. contributed in collection and assembly of data; P.D., H.G., J.Y., and S.C. performed Data analysis and interpretation; P.D., H.G., J.Y., and S.C. wrote the manuscript; and all authors gave the final approval of manuscript.

## Supporting information



Supporting Information

## Data Availability

The participant data with identifiers used to support the findings of this study were supplied by Qun Zhao under license and thus cannot be made freely available. The requests for access to these data should be made to Qun Zhao, zhaoqun@hebmu.edu.cn.
