# Supplementary material for: Interpretable Multimodal Fusion Model Enhances Postoperative Recurrence Prediction in Gastric Cancer
Source: Adv Sci (Weinh). 2025 Sep 13;12(43):e08190. doi: 10.1002/advs.202508190 (PMC12631903; doi:10.1002/advs.202508190)
Supplement: Supplementary file 1 — Supporting Information [file ADVS-12-e08190-s001.docx]

**Supporting Information**

**Supplementary Figures**······································1

**Supplementary Tables**······································23

**Supplementary Methods**·································141

**Supplementary Figures**

**
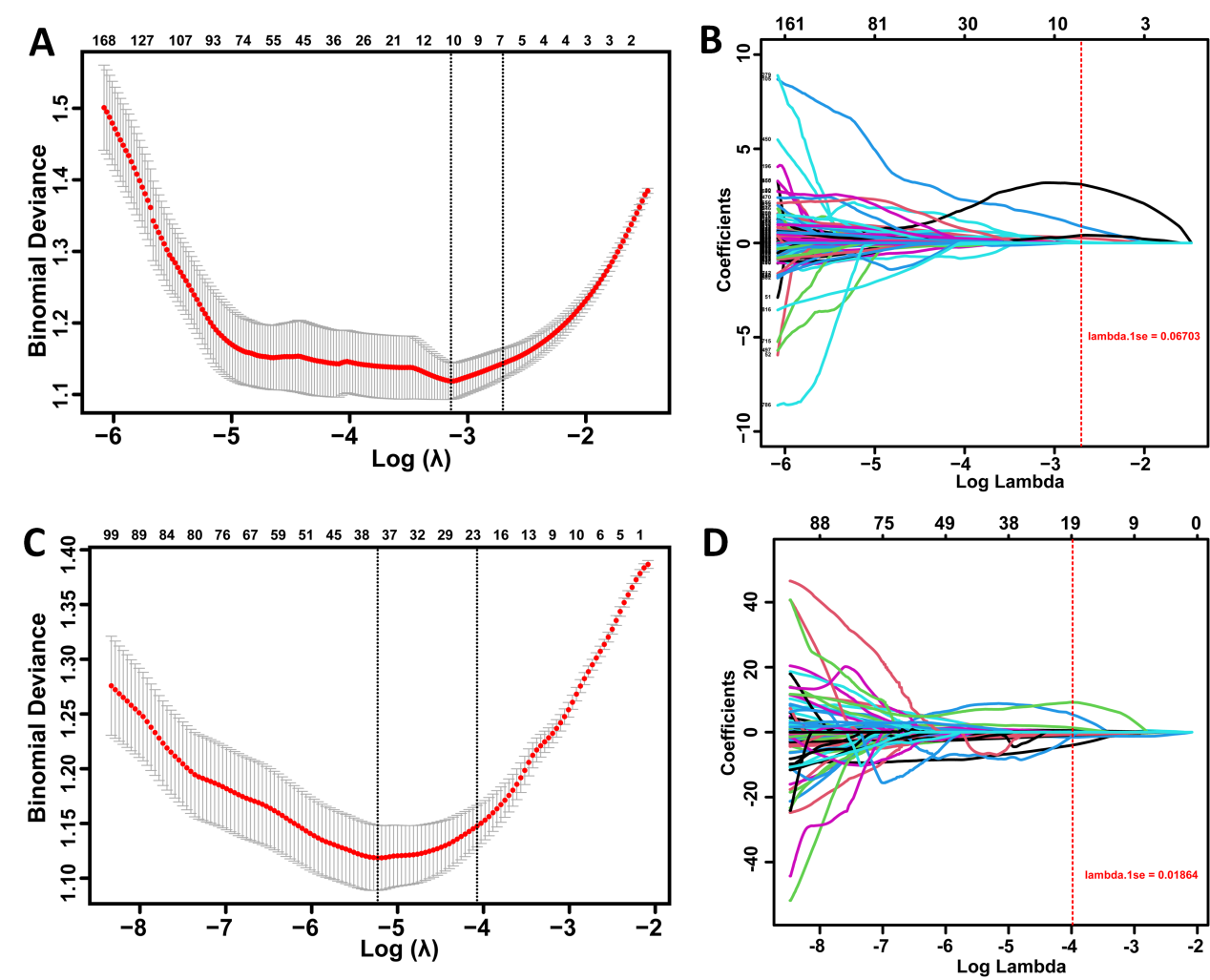
**

**Figure S1. LASSO regression was employed to reduce the radiomic and pathomic features in the training cohort.** Specifically, LASSO was used for feature selection to identify candidate predictors. **(A)** Ten-fold cross-validation was applied to determine the optimal penalty parameter lambda (λ) for the radiomic feature selection model. To prevent overfitting, the "one-standard-error" rule was used, yielding an optimal λ value of 0.06703 and determining the number of selected features. **(B)** Among the 816 radiomic features, the distribution of LASSO coefficients at the optimal λ resulted in 6 non-zero coefficients. **(C)** Similarly, ten-fold cross-validation was used to identify the optimal λ (0.01864) for the pathomic feature selection model, also based on the one-standard-error rule. **(D)** Among the 512 pathomic features, the LASSO coefficient distribution at the optimal λ yielded 21 non-zero coefficients.

**
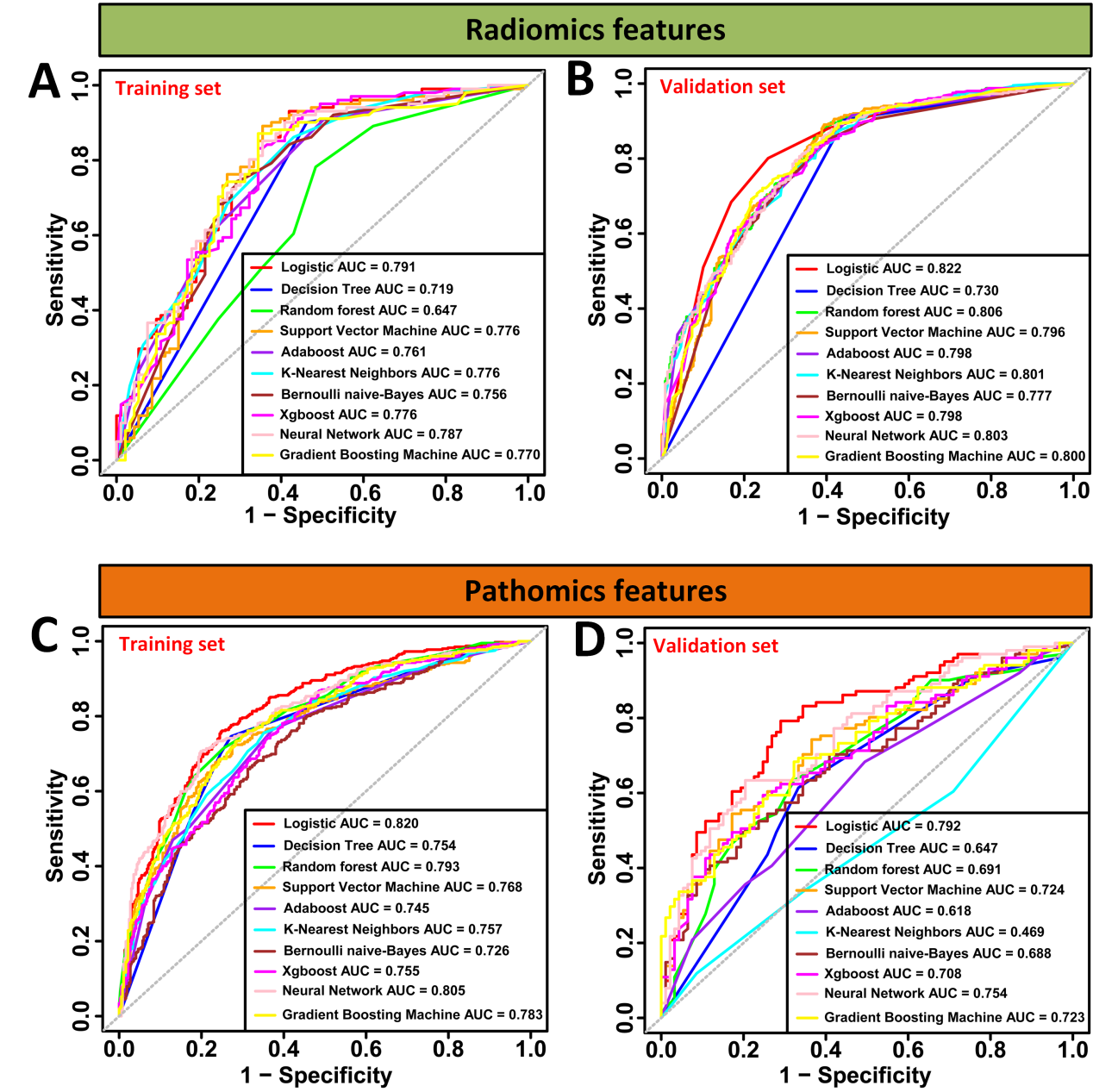
**

**Figure S2. Classification performance of the radiomic and pathomic models was evaluated in both the training and validation cohorts. This figure illustrates the classification performance of ten machine learning algorithms applied to the radiomic and pathomic models, quantified using ROC curves and the corresponding AUC values. (A)** In the radiomic model for the training set, logistic regression (AUC = 0.791) and neural network (AUC = 0.787) achieved the highest performance. **(B)** In the radiomic model for the validation set, logistic regression (AUC = 0.822) and random forest (AUC = 0.806) performed best. **(C)** For the pathomic model in the training set, logistic regression (AUC = 0.820) and neural network (AUC = 0.805) showed the highest accuracy. **(D)** In the pathomic model for the validation set, logistic regression (AUC = 0.792) and neural network (AUC = 0.754) achieved the best classification performance.


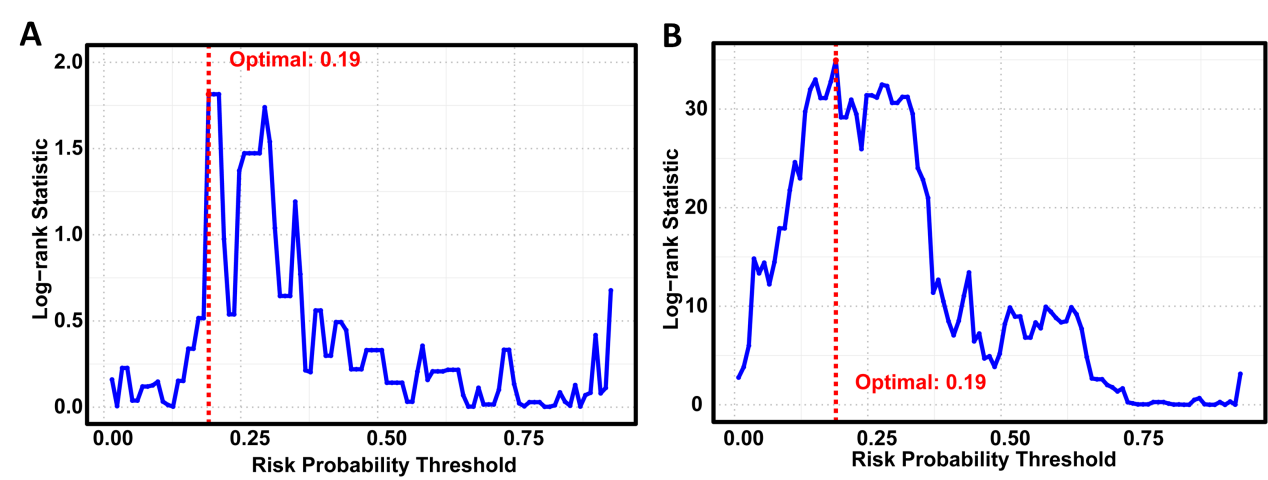


**Figure S3. Optimal Threshold Identification for Predicting Benefit from Adjuvant Chemotherapy Using the RSA Model.** Line plot illustrating the maximally selected log-rank statistics for various RSA-predicted recurrence probability thresholds. Each threshold was tested to stratify patients into low-risk and high-risk groups, and the log-rank statistic was computed for the survival difference between groups. (A) Internal validation cohort: the optimal threshold was identified at 0.19, corresponding to the maximum log-rank statistic. (B) External validation cohort: a similar threshold of 0.19 was observed, indicating good reproducibility and robustness of the cut-off across independent cohorts. The red dashed line indicates the optimal threshold selected in each cohort.


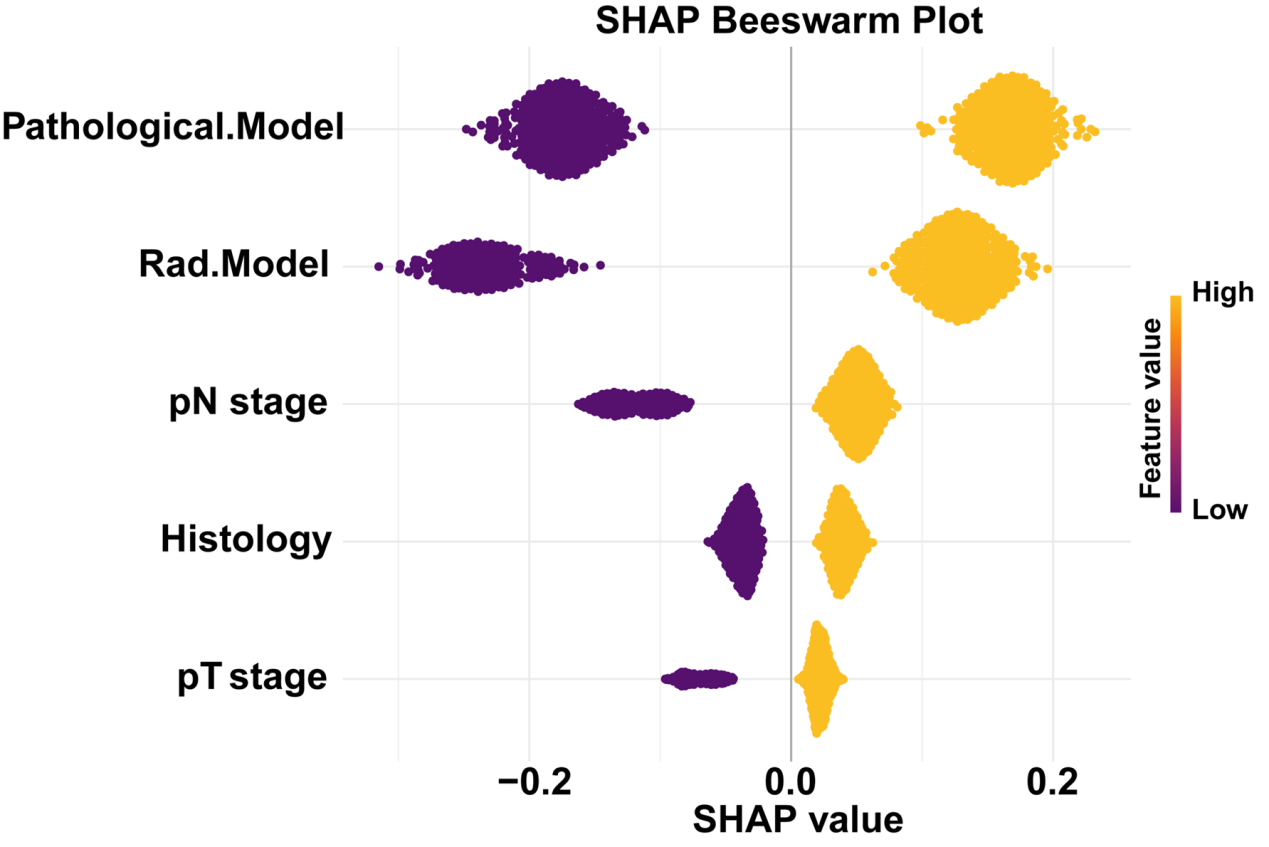


**Figure S4. SHAP beeswarm plot illustrating the relative contribution of each component to the RSA model.** This plot displays the SHAP values of the five final input variables used in the RSA model, including the radiomic score, pathomic score, and three clinical features (pT stage, pN stage, and histology). Each point represents a single patient. The x-axis indicates the SHAP value, which reflects the direction and magnitude of a variable’s impact on recurrence prediction. Features on the left contribute negatively (decreasing risk), while those on the right contribute positively (increasing risk). Colors represent the original feature values, with yellow indicating high values and purple indicating low values. The radiomic and pathomic scores exhibit the largest SHAP value distributions, highlighting their dominant influence on the model output compared to clinical features.


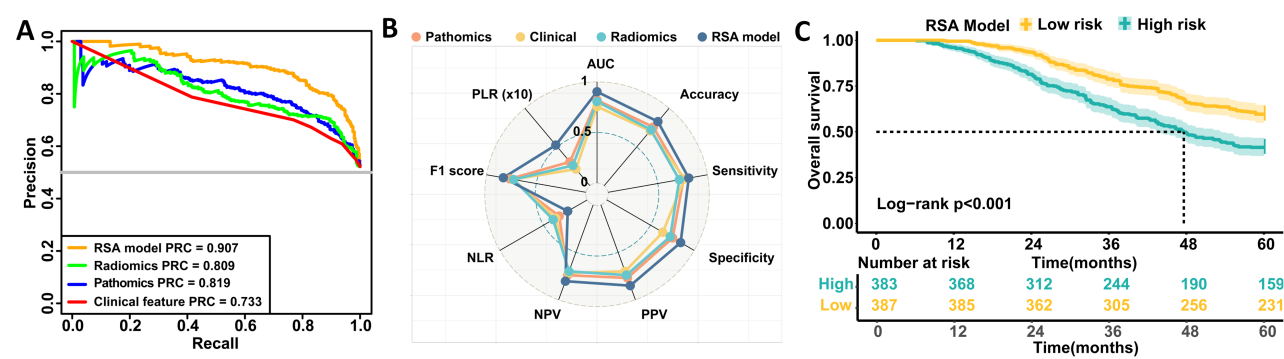


**Figure S5. (A)** Precision-recall curves of various predictive models in the training cohort. **(B)** Radar plot illustrating the performance metrics of different predictive models in the training cohort. **(C)** Kaplan-Meier survival curves with log-rank tests stratifying training cohort patients into low-risk and high-risk groups, based on optimal Youden index cutoff from the nomogram.

**
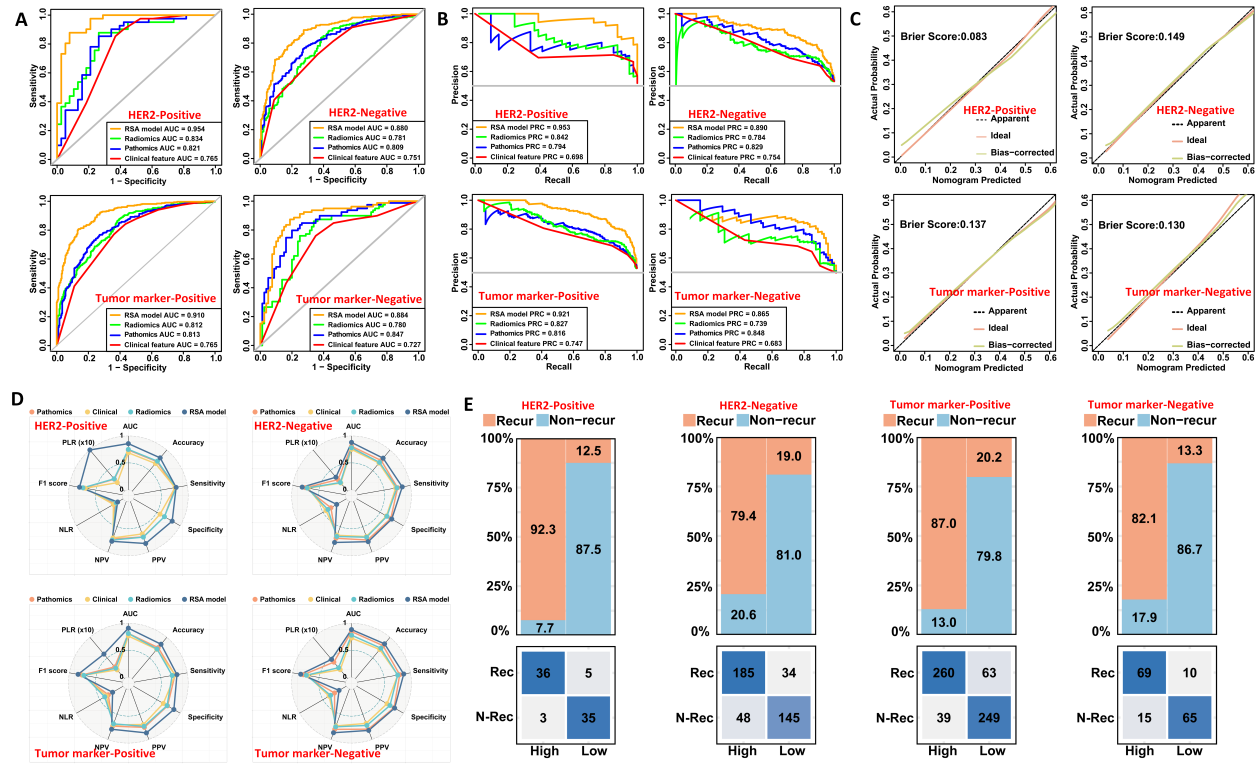
**

**Figure S6. The training process of the RSA model for predicting early recurrence in patients with LAGC was conducted through a stratified analysis based on the expression levels of tumor markers in peripheral blood and HER2 molecular status in biopsy tissue.** **(A)** ROC curves of different predictive models across stratified subgroups in the validation cohort. **(B)** Precision recall curves of different predictive models in the stratified validation subgroups. **(C)** Calibration curves of various models across stratified validation subgroups. **(D)** Radar plots comparing the predictive performance of different models in each stratified validation subgroup. **(E)** Confusion matrices of the predictive models across the stratified validation subgroups.

**
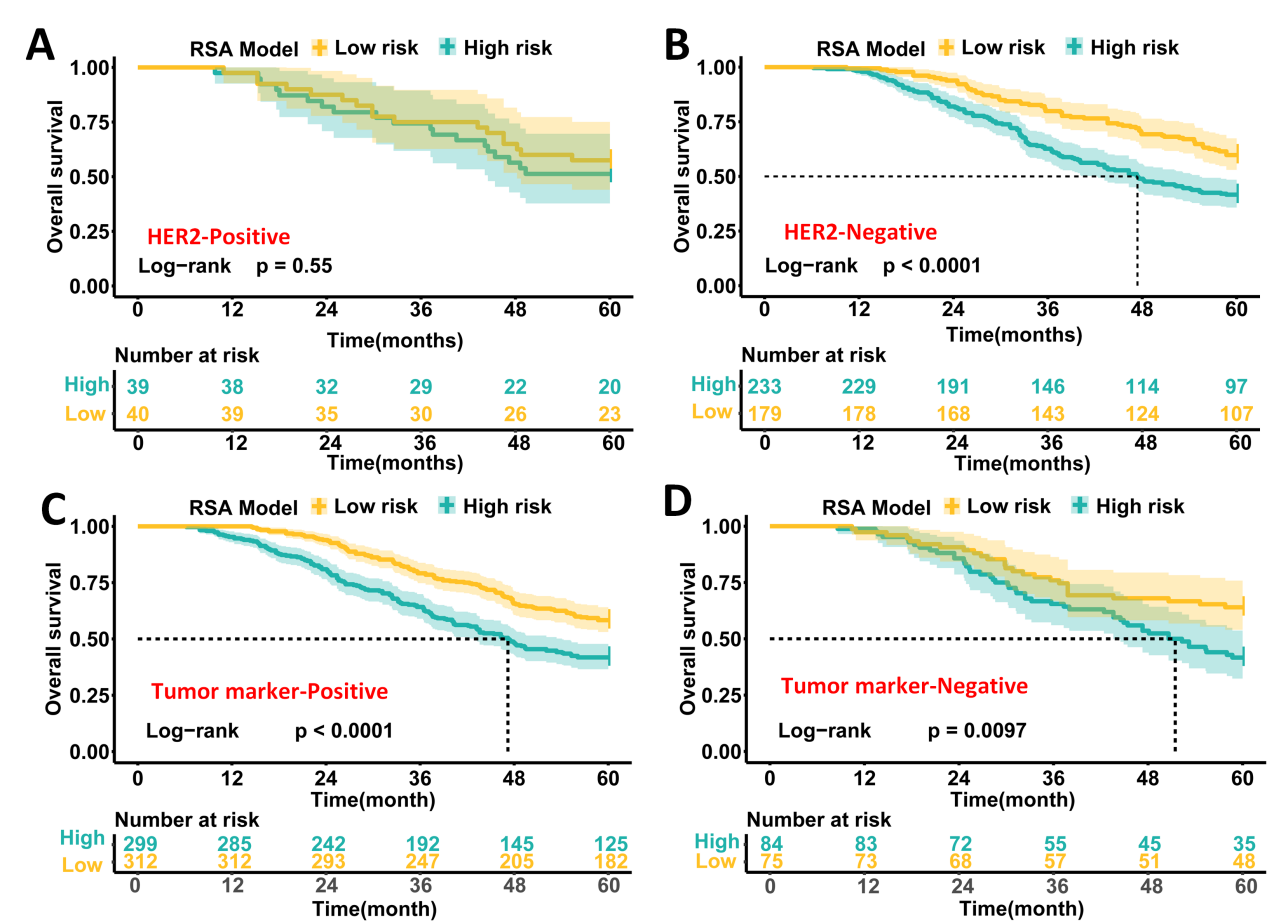
**

**Figure S7. Survival analysis of high- and low-risk groups defined by the RSA model was conducted through stratified analysis based on HER2 expression status in biopsy tissue and tumor marker levels in peripheral blood. (A)** Five-year overall survival comparison in the HER2-positive subgroup. **(B)** Five-year overall survival comparison in the HER2-negative subgroup. **(C)** Five-year overall survival comparison in the peripheral blood tumor marker positive subgroup. **(D)** Five-year overall survival comparison in the peripheral blood tumor marker negative subgroup.

**
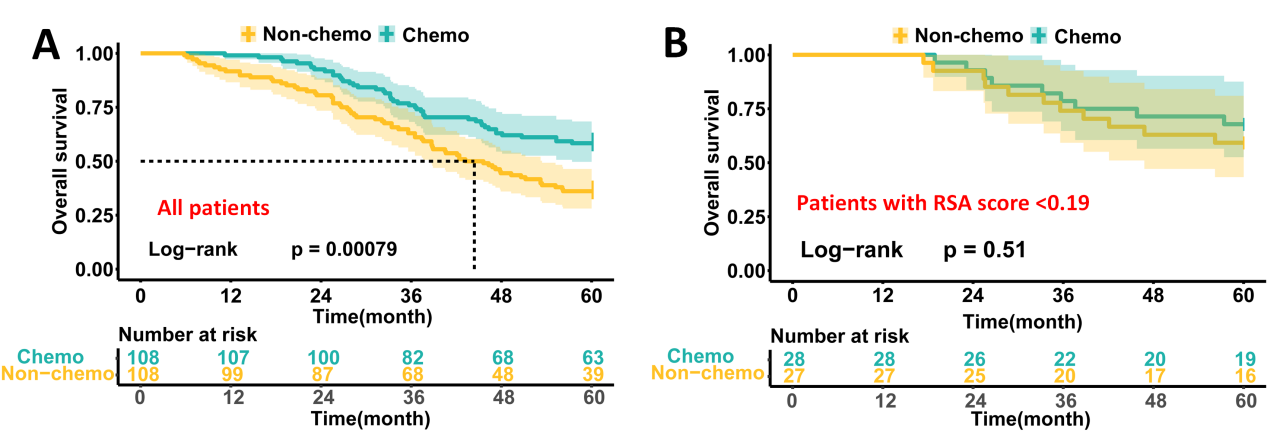
**

#### **Figure S8. After propensity score matching, Kaplan–Meier survival curves were used to compare overall survival between patients who received adjuvant chemotherapy and those who did not, stratified by RSA model–predicted risk probabilities in the training cohort. (A)** Five-year overall survival comparison between patients who received AC and those who did not, following 1:1 propensity score matching. **(B)** Five-year overall survival comparison in the low-risk subgroup (RSA-predicted risk probability < 0.19) between patients who received AC and those who did not.

####
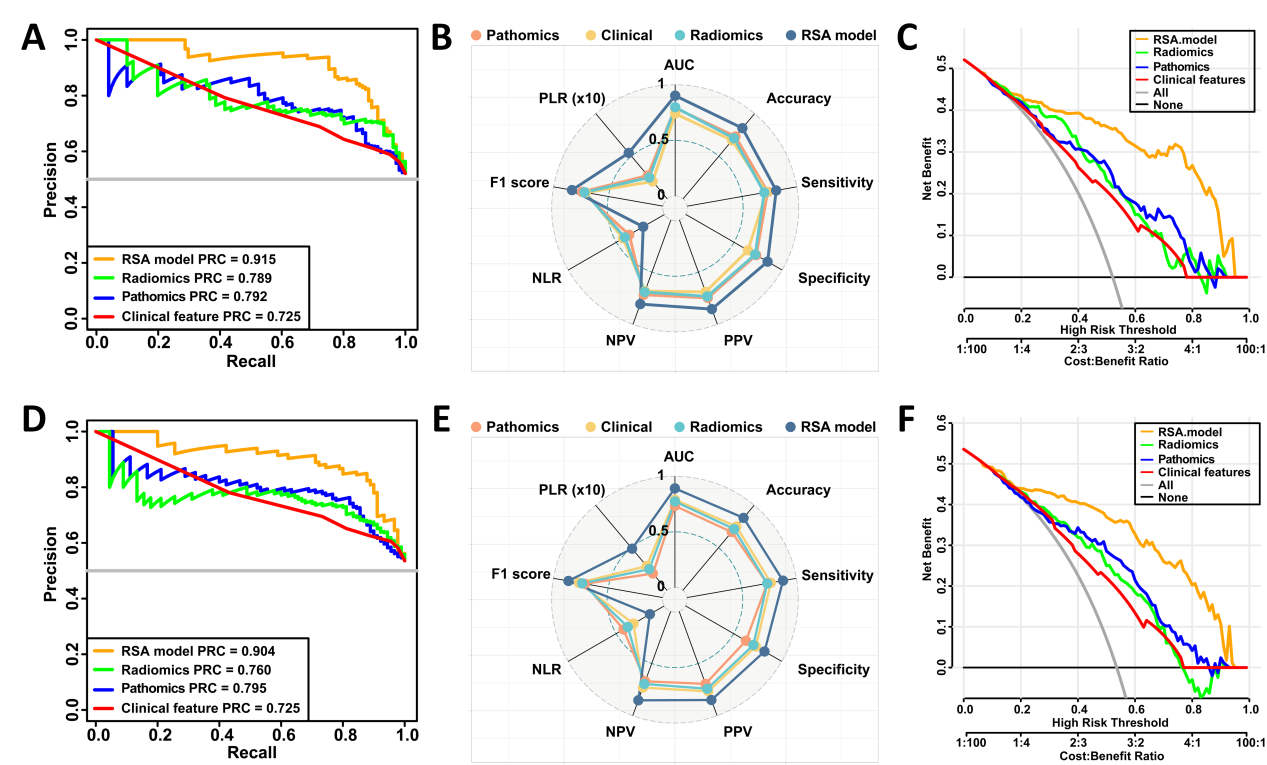


**Figure S9. (A)** Precision-Recall curves of various predictive models in Internal Validation Set I.**(B)** Radar plot illustrating the performance metrics of different predictive models in Internal Validation Set I. **(C)** Decision Curve Analysis curves for different predictive models in Internal Validation Set I. **(D)** Precision-Recall curves of various predictive models in Internal Validation Set II. **(E)** Radar plot illustrating the performance metrics of different predictive models in Internal Validation Set II. **(F)** Decision Curve Analysis curve of the RSA model in Internal Validation Set II.

**
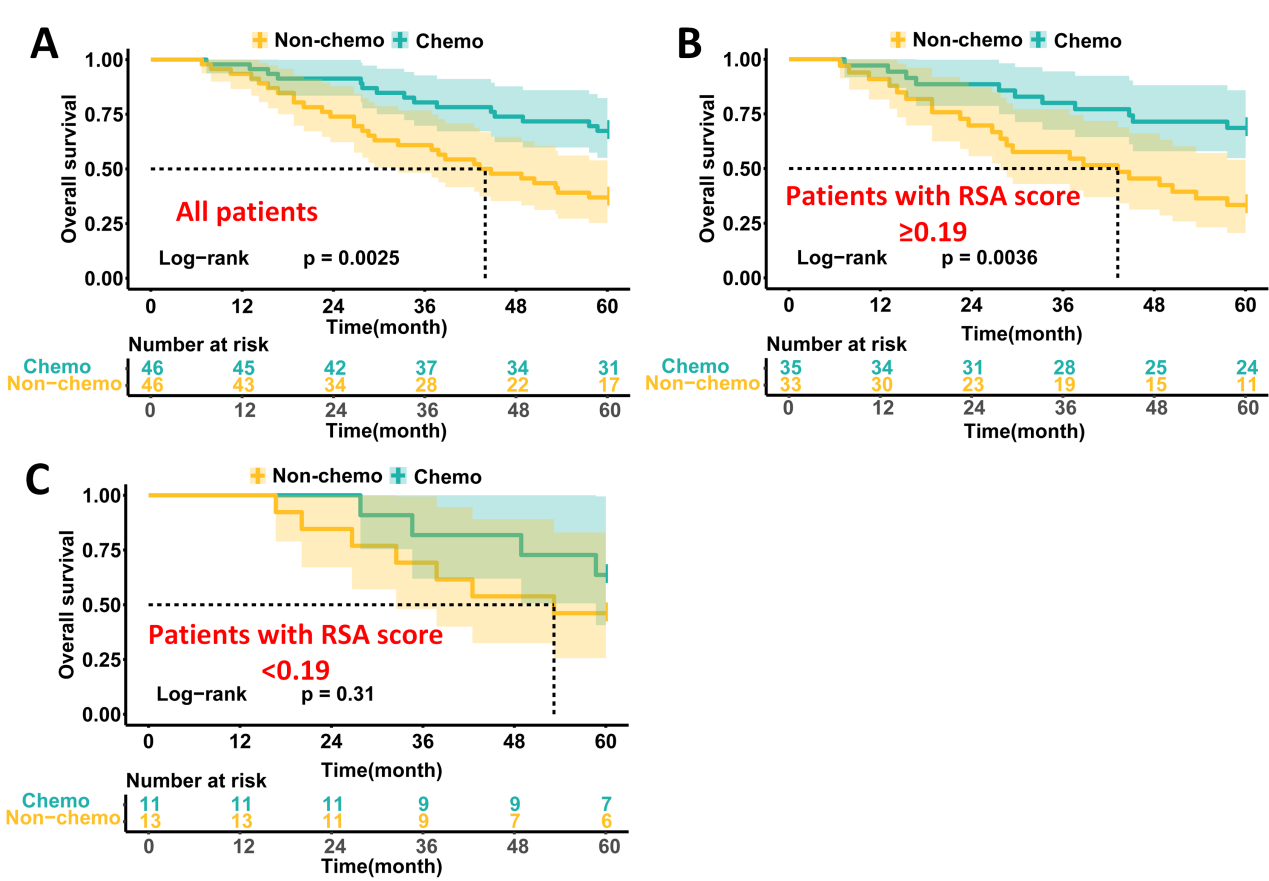
**

#### **Figure S10. After propensity score matching, Kaplan–Meier survival curves were used to compare overall survival between patients who received adjuvant chemotherapy and those who did not, stratified by RSA model–predicted risk probabilities in the internal validation cohort. (A)** Five-year overall survival comparison between patients who received AC and those who did not after 1:1 propensity score matching. **(B)** Five-year overall survival comparison in the low-risk subgroup (RSA-predicted probability < 0.19) between patients who received AC and those who did not.**(C)** Five-year overall survival comparison in the high-risk subgroup (RSA-predicted probability ≥ 0.19) between patients who received AC and those who did not.

**
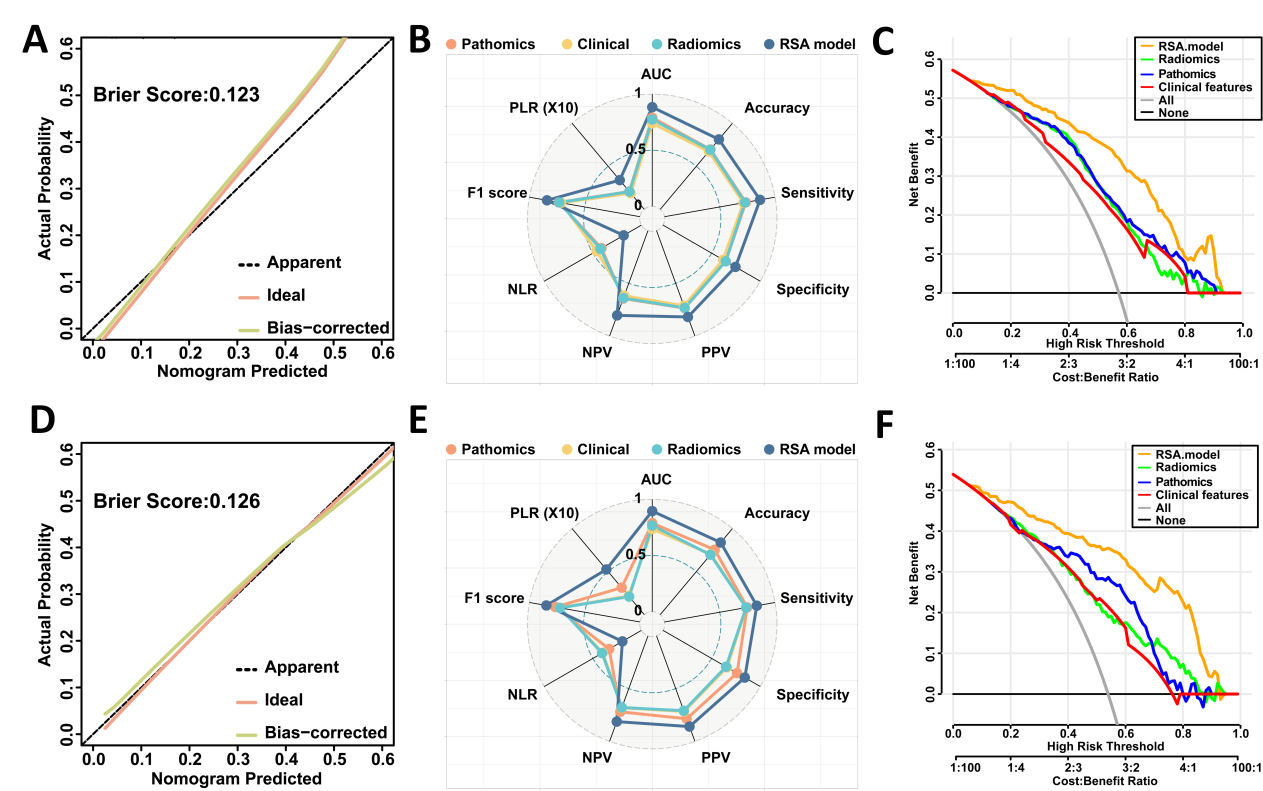
**

**Figure S11. (A)** Calibration curve of the RSA model in External Validation Set I. **(B)** Radar plot illustrating the performance metrics of different predictive models in External Validation Set I. **(C)** Decision Curve Analysis curves for different predictive models in External Validation Set I. **(D)** Calibration curve of the RSA model in External Validation Set II. **(E)** Radar plot illustrating the performance metrics of different predictive models in External Validation Set II. **(F)** Decision Curve Analysis curves curves for various predictive models in External Validation Set II.

**
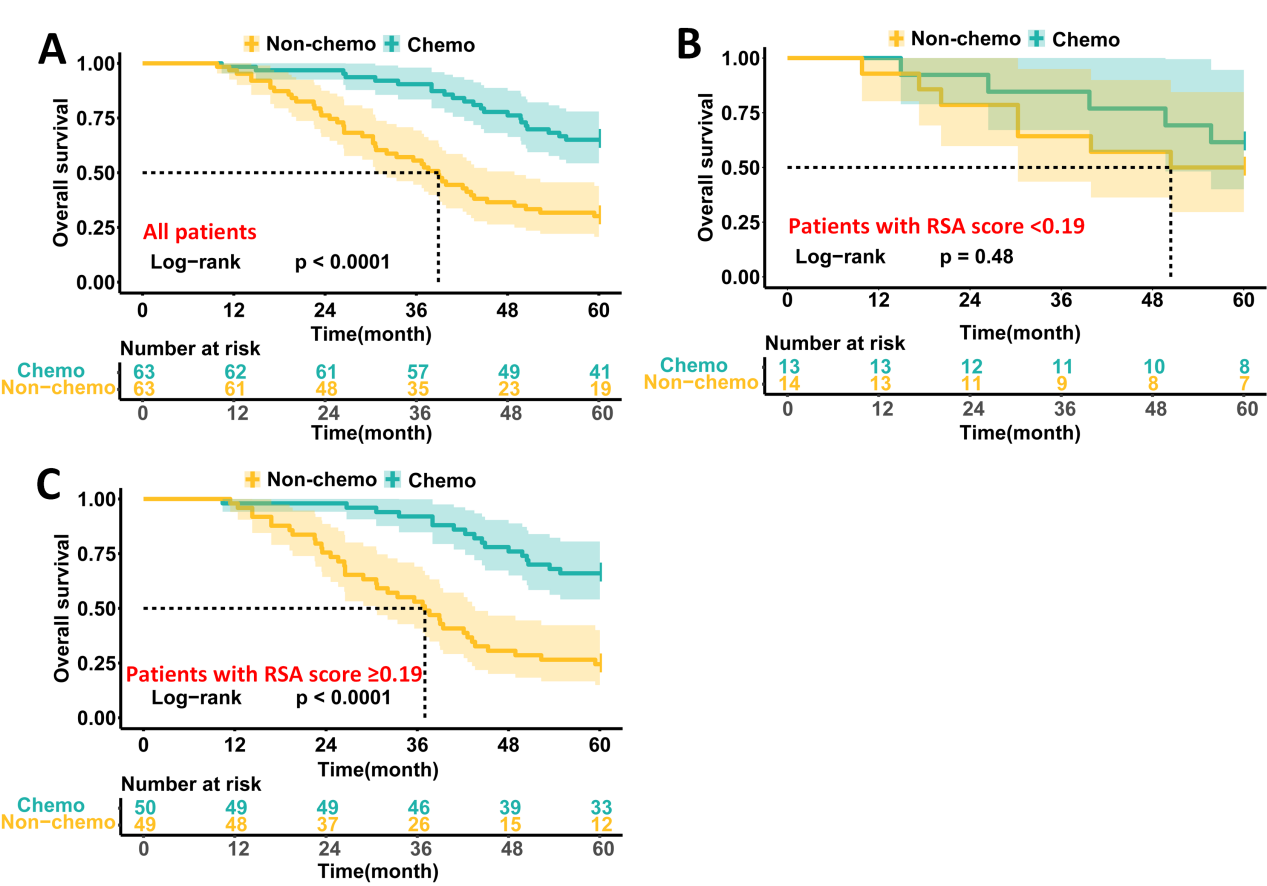
**

**Figure S12. After propensity score matching, Kaplan–Meier survival curves were used to compare overall survival between patients who received adjuvant chemotherapy and those who did not, stratified by RSA model predicted risk probabilities in the external validation cohort.** (A) Five-year overall survival comparison between patients who received AC and those who did not after 1:1 propensity score matching. (B) Five-year overall survival comparison in the low-risk subgroup (RSA-predicted probability < 0.19) between patients who received AC and those who did not. (C) Five-year overall survival comparison in the high-risk subgroup (RSA-predicted probability ≥ 0.19) between patients who received AC and those who did not.

**
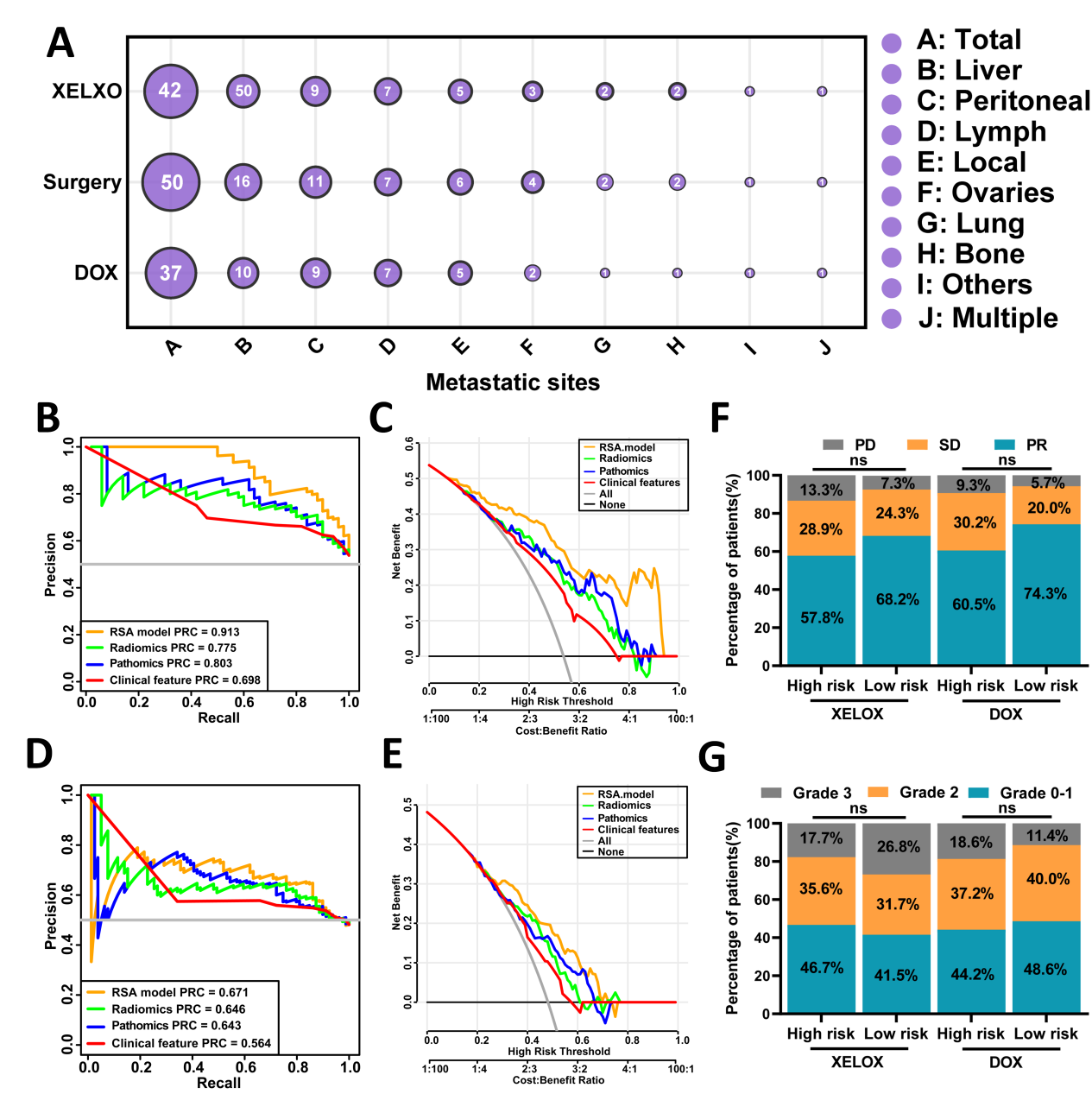
**

**Figure S13. (A)** Postoperative recurrence across different treatment groups within the prospective cohort. (**B)** Precision-Recall curves of various predictive models in the surgery-only subgroup. **(C)** Decision Curve Analysis curves for different predictive models in the surgery-only subgroup. **(D)** Precision-Recall curves of various predictive models in the neoadjuvant chemotherapy subgroup. **(E)** Decision Curve Analysis curves for various predictive models in the neoadjuvant chemotherapy subgroup. **(F)** Comparative analysis of imaging response among different chemotherapy regimens in the neoadjuvant chemotherapy subgroup, stratified by high- and low-risk groups based on Youden index thresholds. **(G)** Comparative analysis of postoperative pathological grade among different chemotherapy regimens in the neoadjuvant chemotherapy subgroup, stratified by high- and low-risk groups based on Youden index thresholds.

**
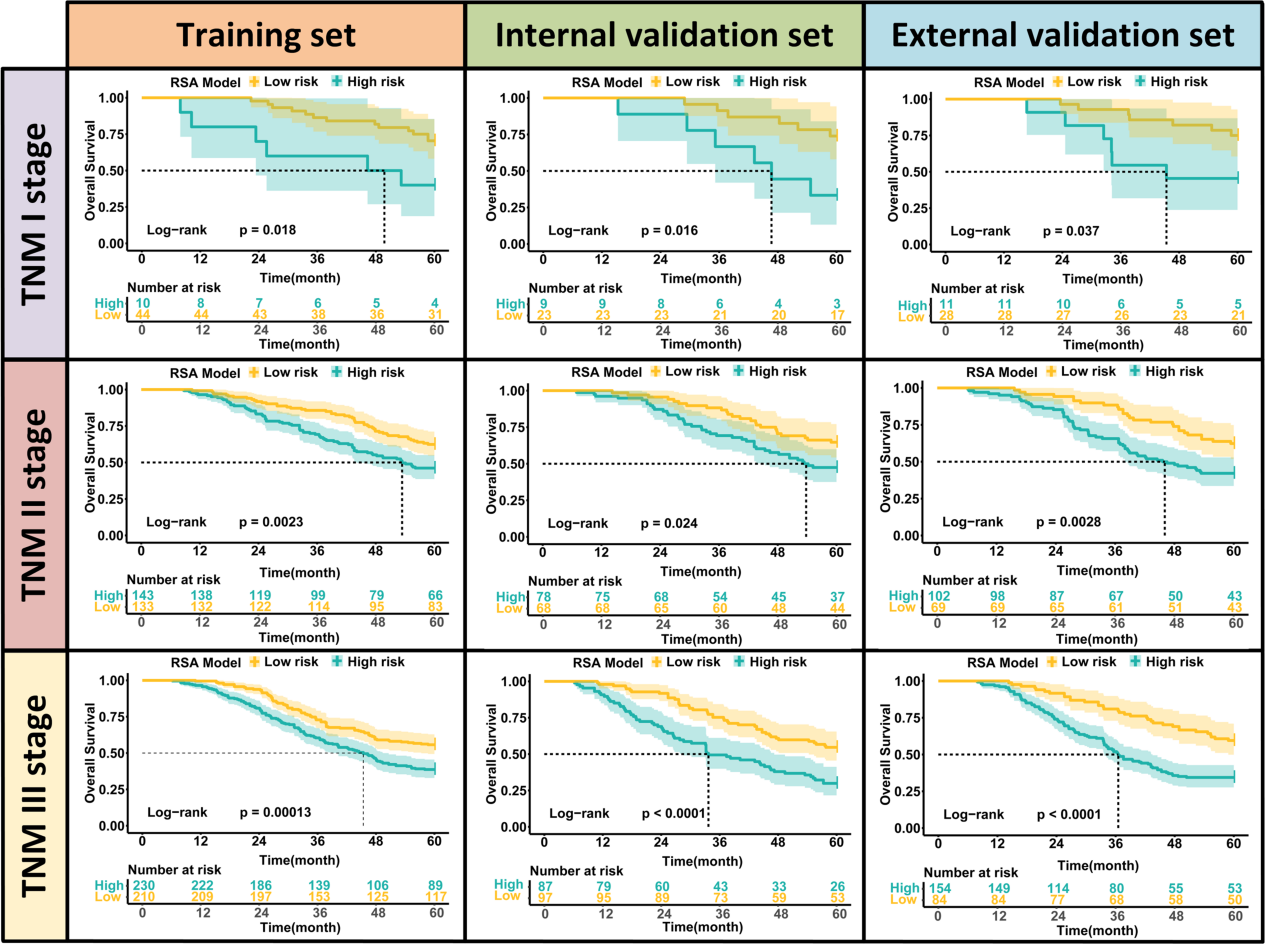
**

**Figure S14.** Prognostic survival analysis of high- and low-risk groups, as defined by the RSA model using Youden index thresholds derived from nomograms, across different TNM stages in the training, internal validation, and external validation cohorts.

**
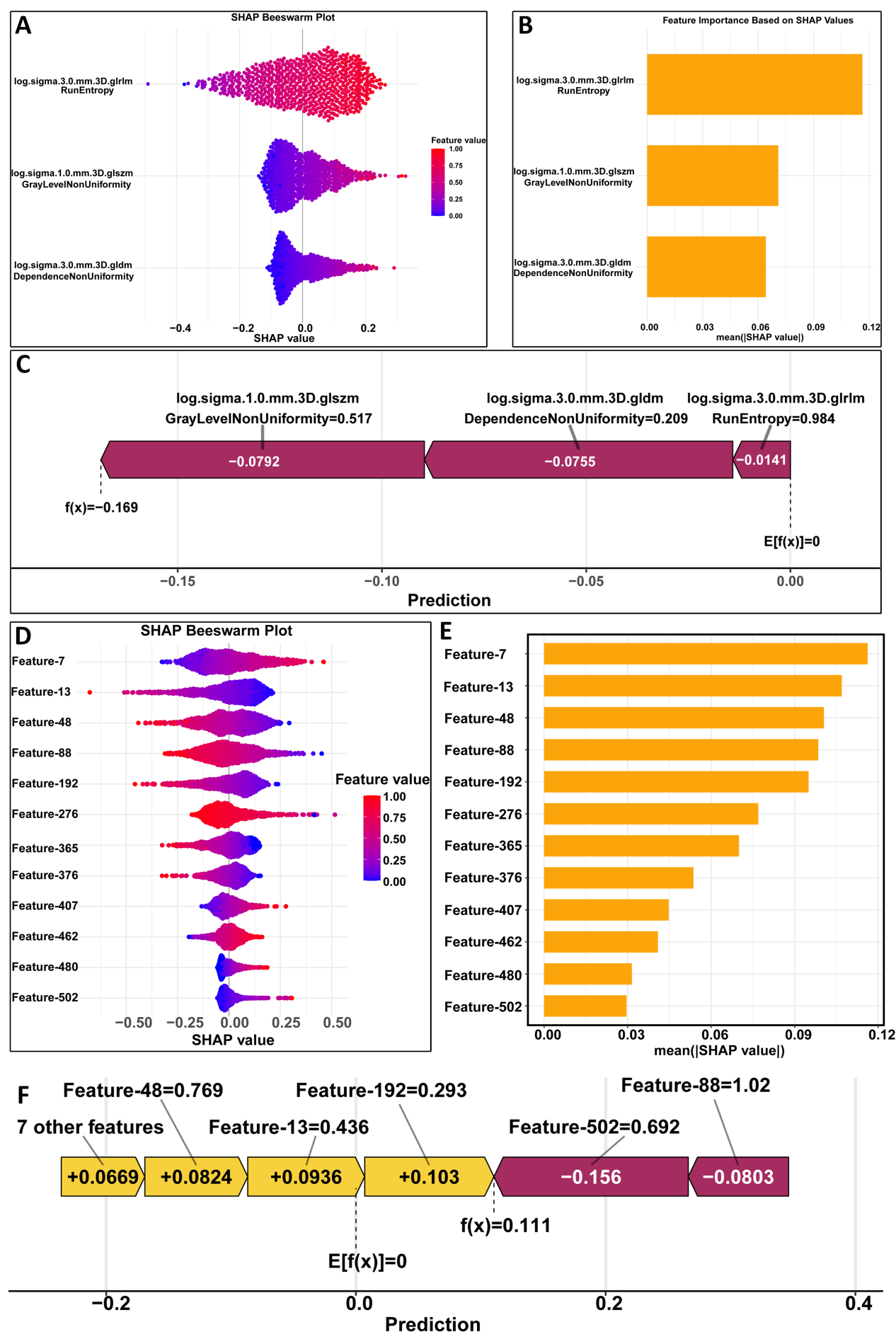
**

**Figure S15. SHAP interpretation of the RSA model. (A-C)** SHAP analysis based on the imaging-based logistic regression model. **(A)** In the SHAP beeswarm plot, each row represents a feature, the x-axis indicates the SHAP value, and each point corresponds to an individual sample. **(B)** Feature importance ranking based on the mean absolute SHAP values. **(C)** SHAP analysis illustrating the contribution of features to recurrence versus non-recurrence in patients with LAGC. **(D-F)** SHAP analysis based on the pathology-based logistic regression model. **(D)** In the SHAP beeswarm plot, each row represents a feature, the x-axis indicates the SHAP value, and each point corresponds to a sample. **(E)** Feature importance ranking based on the mean absolute SHAP values. **(F)** SHAP analysis illustrating the contribution of features to recurrence versus non-recurrence in patients with LAGC.

**
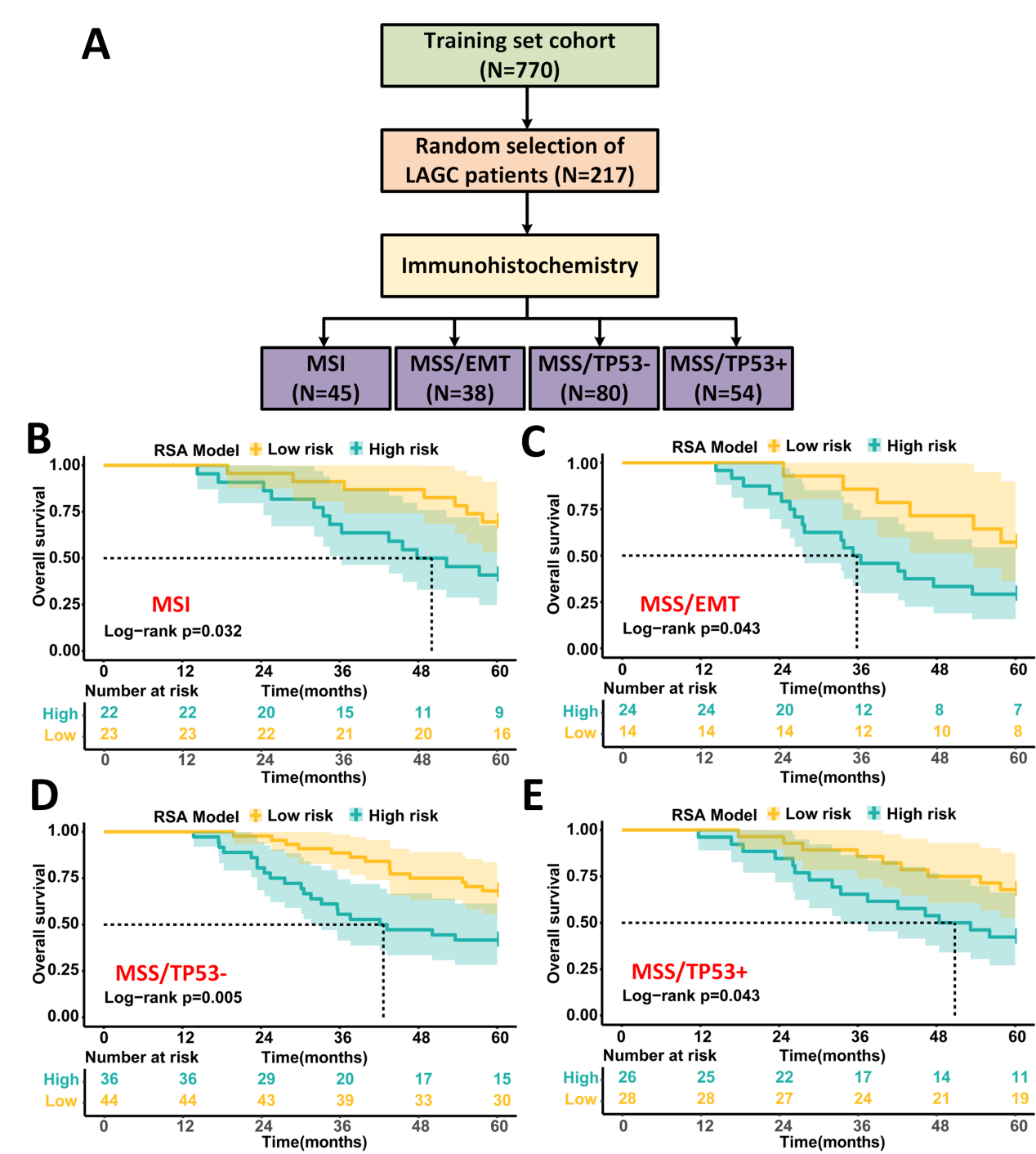
**

**Figure S16. (A)** Flowchart illustrating the selection process for ACRG molecular subtypes. **(B-E)** Survival analysis of RSA-defined high- and low-risk groups within distinct ACRG molecular subtypes: (E) MSI subtype, (F) MSS/EMT subtype, (G) MSS/TP53− subtype, and (H) MSS/TP53+ subtype.

**
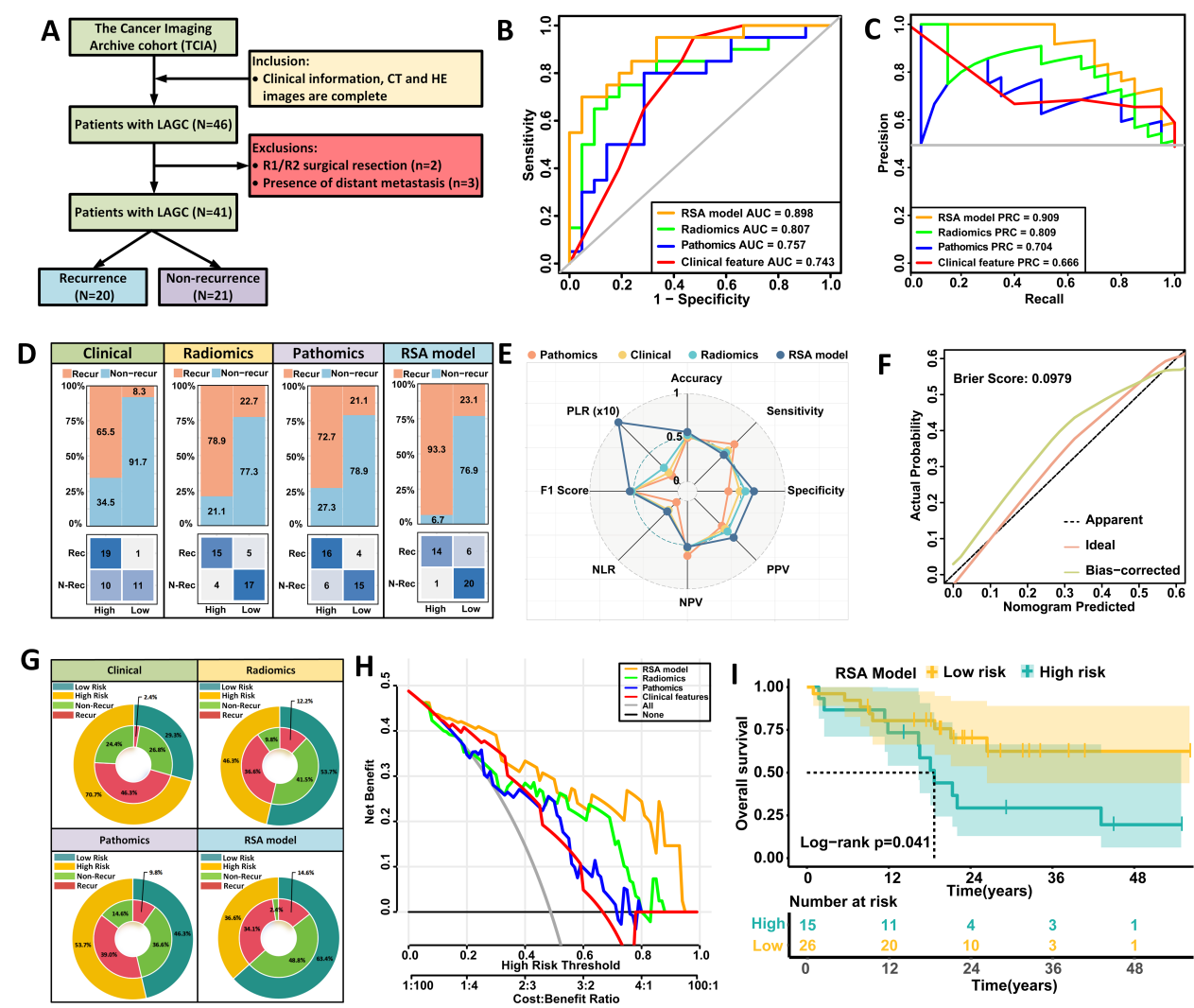
**

**Figure S17. External validation of the RSA model in TCIA cohort. (A)** Inclusion and exclusion criteria for the TCIA external dataset. **(B)** ROC curves of various predictive models in the TCIA validation cohort. **(C)** Precision-Recall curves of various predictive models in the TCIA validation cohort. **(D)** Confusion matrices of different predictive models in the TCIA validation cohort. **(E)** Radar plot illustrating the performance metrics of various predictive models in the TCIA validation cohort. **(F)** Calibration curve of the RSA model in the TCIA validation cohort. **(G)** Double-layer concentric circle diagram demonstrating the clinical utility of different predictive models in the TCIA validation cohort. **(H)** Decision Curve Analysis curves of various predictive models in the TCIA validation cohort. **(I)** Kaplan-Meier survival curves with log-rank test results for patients in the TCIA validation cohort, stratified into high- and low-risk groups based on Youden index thresholds derived from nomograms.

**
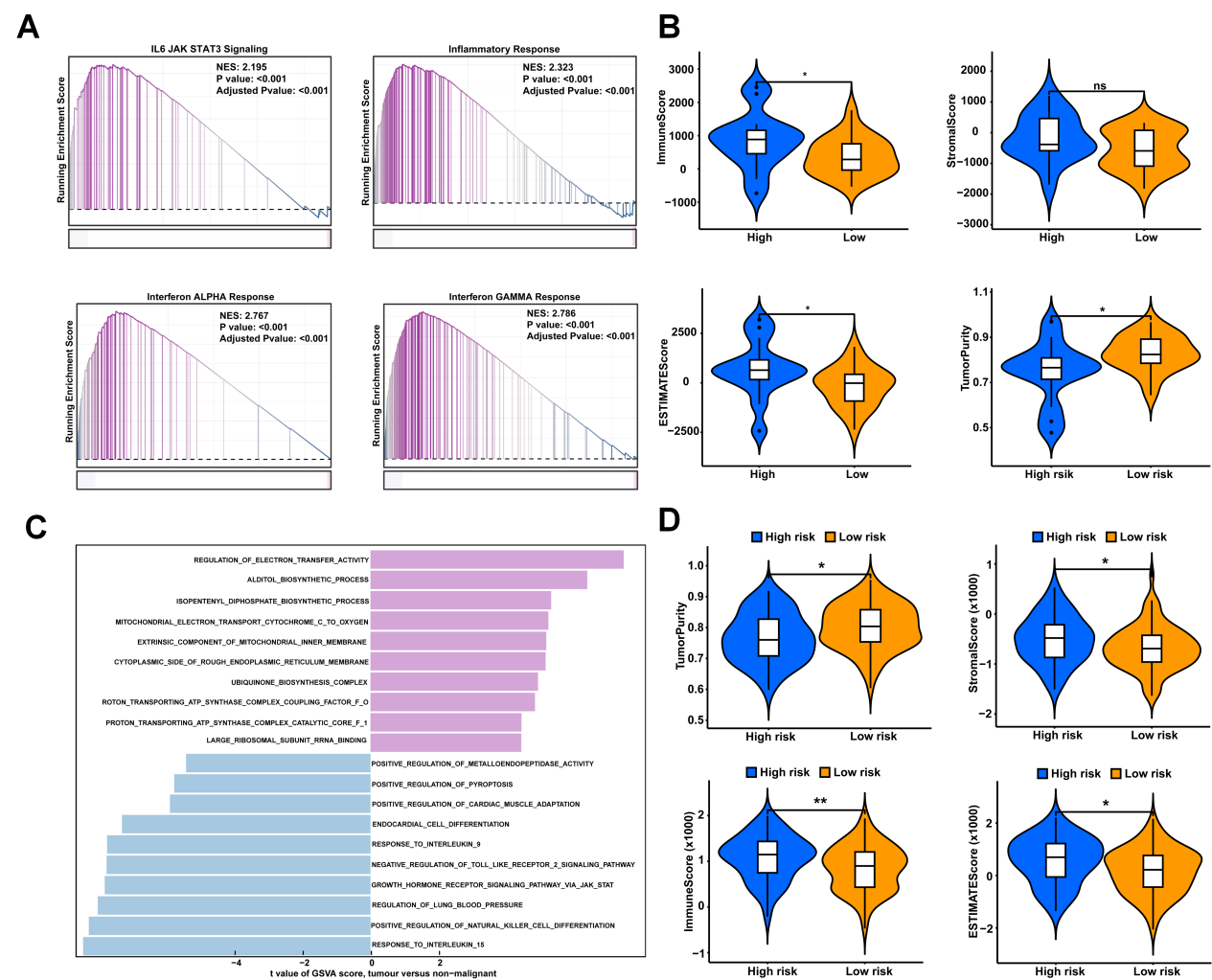
**

**Figure S18. Differences in pathway enrichment and immune microenvironment between high-risk and low-risk groups in TCGA and transcriptome sequencing gastric cancer cohorts. (A)** Results of GSEA enrichment analysis of TCGA gastric cancer samples. **(B)** The violin plots show the differences in tumor purity, immune score, ESTIMATE score, and stromal score between high-risk and low-risk group cancer tissues. **(C)** GSVA enrichment analysis results of RNA sequencing data of gastric cancer samples. **(D)** ESTIMATE analysis evaluates differences in immune microenvironment between high-risk and low-risk groups. (ns = not significant, *P < 0.05, **P < 0.01).

**
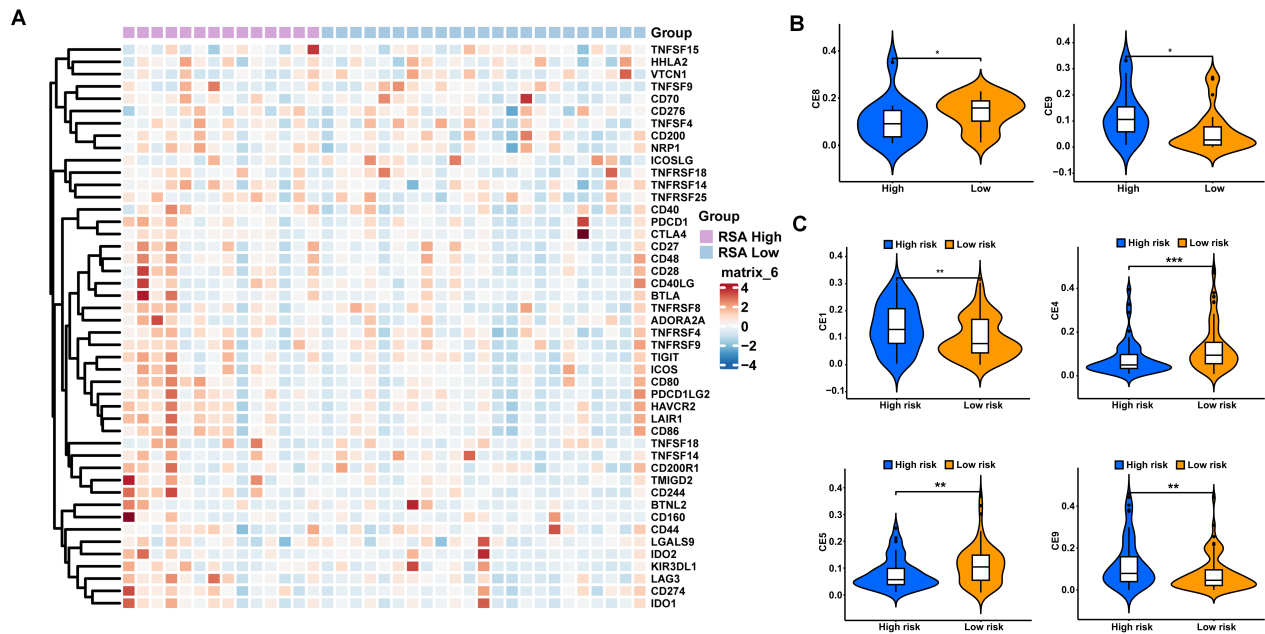
**

**Figure S19. Differences in immune ecology between high-risk and low-risk groups in TCGA and transcriptome sequencing gastric cancer cohorts.** (A) Heat map showing the expression clustering patterns of immune checkpoint genes in cancer tissues of high- and low-risk groups. (B-C) Violin plot showing differences in cancer ecosystem abundance of TCGA (B) and RNA sequencing data (C) between sequencing samples in high-risk and low-risk groups. (*P < 0.05, **P < 0.01, ***P < 0.001).

**
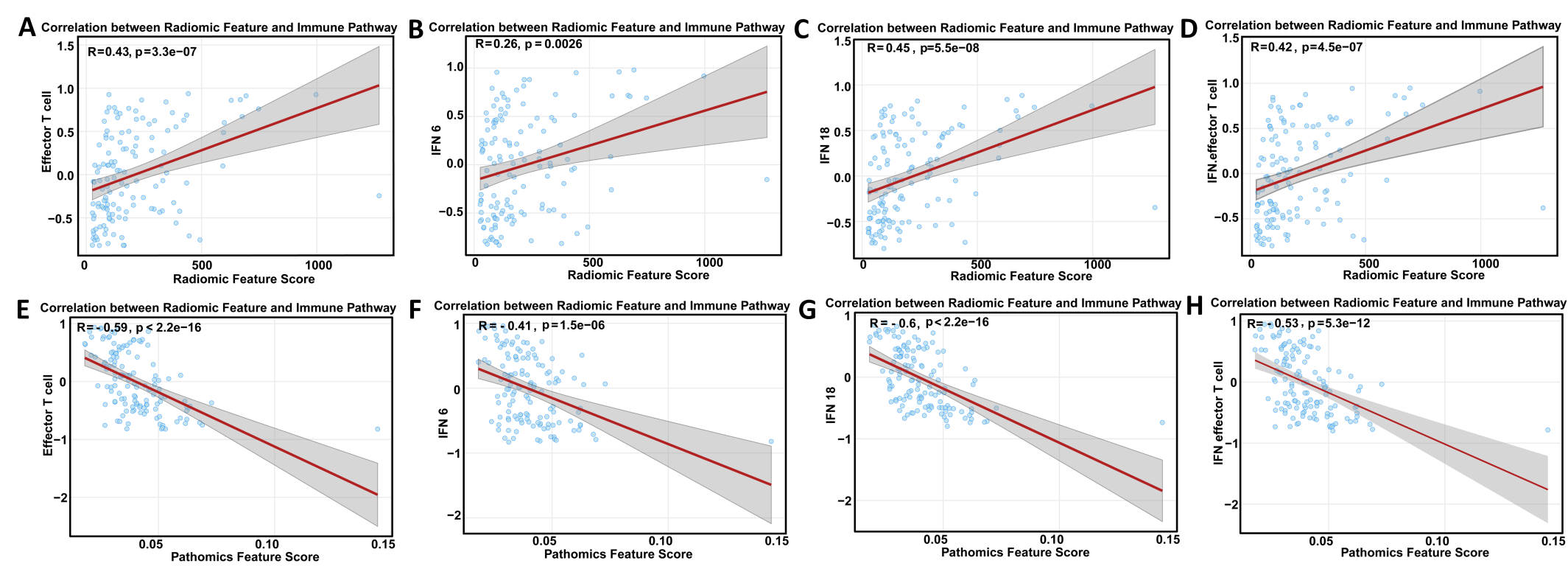
**

**Figure S20. Correlation between multimodal imaging-derived feature scores and immune-related pathway activity.** (A-D) show Spearman correlations between radiomic feature scores and immune activation signatures, including Effector T cell (A), IFNγ (B), IFNα (C), and IFN effector T cell response (D). Higher radiomic scores were significantly associated with increased immune activity across all modules. (E-H) display Spearman correlations between pathomic feature scores and the same immune pathways. In contrast to radiomics, higher pathomic scores were negatively correlated with immune activation, suggesting an immune-suppressed tumor phenotype. Shaded areas represent 95% confidence intervals for the regression lines. All correlations were statistically significant, with corresponding R and P values indicated in each panel.

**Supplementary Tables**

**Table S1. Predictive performance of different machine learning models based on radiomics features**

| **Radiomics Model** | **AUC (95%CI)** | **Accuracy (95%CI)** | **Sensitivity (95%CI)** | **Specificity (95%CI)** |
| --- | --- | --- | --- | --- |
| **Training set** |  |  |  |  |
| Logistic | **0.822(0.793-0.852)** | **0.749 (0.717-0.779)** | **0.881 (0.844-0.910)** | **0.606 (0.554-0.656)** |
| Decision Tree | **0.73 (0.701-0.759)** | **0.738 (0.705-0.768)** | **0.903 (0.869-0.929)** | **0.557 (0.505-0.608)** |
| Random forest | **0.806 (0.776-0.837)** | **0.773 (0.741-0.802)** | **0.801 (0.758-0.838)** | **0.742 (0.693-0.785)** |
| Support Vector Machine | **0.796 (0.765-0.828)** | **0.739 (0.706-0.769)** | **0.821 (0.779-0.856)** | **0.649 (0.598-0.698)** |
| Adaboost | **0.798 (0.768-0.827)** | **0.709 (0.675-0.741)** | **0.654 (0.605-0.700)** | **0.769 (0.722-0.810)** |
| K-Nearest Neighbors | **0.801 (0.771-0.832)** | **0.721 (0.687-0.752)** | **0.786 (0.742-0.824)** | **0.649 (0.598-0.698)** |
| Bernoulli naive-Bayes | **0.777 (0.744-0.809)** | **0.719 (0.686-0.751)** | **0.736 (0.690-0.778)** | **0.701 (0.651-0.747)** |
| Xgboost | **0.798 (0.766-0.829)** | **0.727 (0.694-0.758)** | **0.746 (0.700-0.788)** | **0.707 (0.657-0.752)** |
| Neural Network | **0.803 (0.773-0.834)** | **0.716 (0.682-0.747)** | **0.706 (0.659-0.750)** | **0.726 (0.676-0.770)** |
| Gradient Boosting Machine | **0.800 (0.769-0.831)** | **0.696 (0.662-0.728)** | **0.782 (0.730-0.827)** | **0.642 (0.597-0.685)** |
| **Validation set** |  |  |  |  |
| Logistic | **0.791 (0.727-0.855)** | **0.763 (0.696-0.820)** | **0.931(0.858-0.969)** | **0.581 (0.474-0.681)** |
| Decision Tree | **0.719 (0.661-0.778)** | **0.727(0.657-0.787)** | **0.901 (0.821-0.949)** | **0.538 (0.432-0.641)** |
| Random forest | **0.647 (0.570-0.723)** | **0.588(0.515-0.657)** | **0.604 (0.501-0.698)** | **0.570 (0.463-0.671)** |
| Support Vector Machine | **0.776 (0.707-0.846)** | **0.747(0.679-0.806)** | **0.832 (0.741-0.896)** | **0.660 (0.549-0.749)** |
| Adaboost | **0.761 (0.696-0.826)** | **0.686(0.615-0.749)** | **0.614 (0.511-0.708)** | **0.763 (0.662-0.843)** |
| K-Nearest Neighbors | **0.776 (0.712-0.841)** | **0.706(0.636-0.768)** | **0.683 (0.582-0.770)** | **0.731 (0.628-0.815)** |
| Bernoulli naive-Bayes | **0.756 (0.688-0.825)** | **0.722(0.652-0.782)** | **0.733 (0.634-0.814)** | **0.710 (0.605-0.797)** |
| Xgboost | **0.776 (0.709-0.843)** | **0.706(0.636-0.768)** | **0.752 (0.655-0.831)** | **0.656 (0.549-0.749)** |
| Neural Network | **0.787 (0.721-0.852)** | **0.716(0.647-0.778)** | **0.693 (0.592-0.779)** | **0.742 (0.639-0.825)** |
| Gradient Boosting Machine | **0.770 (0.702-0.839)** | **0.655(0.583-0.720)** | **0.750 (0.628-0.844)** | **0.603 (0.512-0.688)** |

**Table S2. Multivariate analysis of radiomics affecting postoperative recurrence in patients with LAGC**

| **Radiomics features** | **coefficient estimates** | **CI_Lower** | **CI_Upper** | **Std.Error** | **Waldχ** | **P** |
| --- | --- | --- | --- | --- | --- | --- |
| log-sigma-1-0-mm-3D glszm GrayLevelNonUniformity | **0.935** | **0.393** | **1.499** | **0.282** | **3.318** | **＜0.001** |
| log-sigma-3-0-mm-3D gldm DependenceNonUniformity | **0.696** | **0.208** | **1.215** | **0.257** | **2.711** | **＜0.001** |
| log-sigma-3-0-mm-3D glrlm RunEntropy | **5.584** | **3.763** | **7.484** | **0.948** | **5.889** | **＜0.001** |

**Table S3. Performances of three ResNet processing of WSI for gastric cancer prediction in HMU-GC-HE-30K**

| **Variable** | **ResNet 18** | **ResNet 34** | **ResNet 50** |
| --- | --- | --- | --- |
| **AUC (95%CI)** | **0.9996 (0.9968–1.0000)** | **0.9040 (0.8600–0.9420)** | **0.9397 (0.902–0.968)** |
| **ACC (95%CI)** | **0.9779 (0.9632–0.9905)** | **0.5534 (0.5101–0.5962)** | **0.6350 (0.570–0.698)** |
| **SENS (95%CI)** | **0.9759 (0.9502–0.9918)** | **0.5467 (0.4700–0.6200)** | **0.6388 (0.570–0.703)** |
| **SPEC (95%CI)** | **0.9968 (0.9875–0.9997)** | **0.9358 (0.8900–0.9700)** | **0.9476 (0.915–0.975)** |

**Note: AUC= area under the curve: ACC= accuracy; SENS = sensitivity, SPEC= specificity: Cl= confidence interval.**

**Table S4. Predictive performance of different machine learning models based on pathomic features**

| **Pathomic Model** | **AUC (95%CI)** | **Accuracy (95%CI)** | **Sensitivity (95%CI)** | **Specificity (95%CI)** |
| --- | --- | --- | --- | --- |
| **Training set** |  |  |  |  |
| Logistic | **0.820 (0.790-0.849)** | **0.749 (0.717-0.779)** | **0.779 (0.734-0.818)** | **0.717 (0.668-0.762)** |
| Decision Tree | **0.754 (0.720-0.787)** | **0.738 (0.705-0.768)** | **0.746 (0.700-0.788)** | **0.728 (0.679-0.772)** |
| Random forest | **0.793 (0.762-0.825)** | **0.721 (0.687-0.752)** | **0.744 (0.698-0.785)** | **0.696 (0.645-0.742)** |
| Support Vector Machine | **0.768 (0.735-0.801)** | **0.719 (0.686-0.751)** | **0.714 (0.667-0.757)** | **0.726 (0.676-0.770)** |
| Adaboost | **0.745 (0.711-0.779)** | **0.699 (0.665-0.731)** | **0.764 (0.718-0.804)** | **0.628 (0.576-0.677)** |
| K-Nearest Neighbors | **0.757 (0.724-0.791)** | **0.706 (0.673-0.738)** | **0.749 (0.703-0.790)** | **0.660 (0.609-0.708)** |
| Bernoulli naive-Bayes | **0.726 (0.690-0.761)** | **0.668 (0.633-0.701)** | **0.694 (0.646-0.738)** | **0.639 (0.587-0.687)** |
| Xgboost | **0.755 (0.721-0.788)** | **0.691 (0.657-0.723)** | **0.744 (0.698-0.785)** | **0.633 (0.581-0.682)** |
| Neural Network | **0.805 (0.774-0.835)** | **0.732 (0.699-0.763)** | **0.744 (0.698-0.785)** | **0.720 (0.671-0.765)** |
| Gradient Boosting Machine | **0.783 (0.751-0.815)** | **0.677 (0.642-0.709)** | **0.820 (0.764-0.865)** | **0.612 (0.569-0.653)** |
| **Validation set** |  |  |  |  |
| Logistic | **0.792 (0.728-0.856)** | **0.747 (0.679-0.806)** | **0.792 (0.698-0.864)** | **0.699 (0.594-0.787)** |
| Decision Tree | **0.647 (0.572-0.723)** | **0.634 (0.562-0.701)** | **0.653 (0.552-0.744)** | **0.613 (0.506-0.710)** |
| Random forest | **0.691 (0.616-0.766)** | **0.640 (0.567-0.706)** | **0.683 (0.582-0.770)** | **0.591 (0.484-0.691)** |
| Support Vector Machine | **0.724 (0.653-0.796)** | **0.665 (0.593-0.730)** | **0.624 (0.521-0.717)** | **0.710 (0.605-0.797)** |
| Adaboost | **0.618 (0.541-0.696)** | **0.598 (0.525-0.667)** | **0.683 (0.582-0.770)** | **0.505 (0.400-0.61)** |
| K-Nearest Neighbors | **0.469 (0.391-0.547)** | **0.644 (0.572-0.711)** | **0.644 (0.541-0.735)** | **0.645 (0.538-0.740)** |
| Bernoulli naive-Bayes | **0.688 (0.615-0.762)** | **0.634 (0.562-0.701)** | **0.663 (0.562-0.752)** | **0.602 (0.495-0.701)** |
| Xgboost | **0.708 (0.636-0.781)** | **0.613 (0.541-0.682)** | **0.723 (0.623-0.805)** | **0.495 (0.390-0.600)** |
| Neural Network | **0.754 (0.687-0.822)** | **0.660 (0.588-0.725)** | **0.703 (0.603-0.788)** | **0.613 (0.506-0.710)** |
| Gradient Boosting Machine | **0.723 (0.653-0.794)** | **0.639 (0.567-0.706)** | **0.754 (0.624-0.852)** | **0.586 (0.498-0.670)** |

**Table S5. Characteristics of patients in the training, internal validation, and external validation cohorts[n(%)]**

| **Variable** | **Total cohort**  **(n=1580)** | **Training cohort (n=770)** | **Internal validation cohort (n=362)** | **External validation cohort (n=448)** | ***P* value** |
| --- | --- | --- | --- | --- | --- |
| **Gender** |  |  |  |  | **0.076** |
| Male | **1036(65.6%)** | **511(66.4%)** | **220(60.8%)** | **305(68.1%)** |  |
| Female | **544(34.4%)** | **259(33.6%)** | **142(39.2%)** | **143(31.9%)** |  |
| **Age, years** |  |  |  |  | **0.085** |
| ≤65 | **738(46.7%)** | **353(45.8%)** | **187(51.7%)** | **198(44.2%)** |  |
| ＞65 | **842(53.3%)** | **417(54.2%)** | **175(48.3%)** | **250(55.8%)** |  |
| **ECOG PS** |  |  |  |  | **0.057** |
| 0-1 | **779(49.3%)** | **363(47.1%)** | **198(54.7%)** | **218(48.7%)** |  |
| 2 | **801(50.7%)** | **407(52.9%)** | **164(45.3%)** | **230(51.3%)** |  |
| **pT stage** |  |  |  |  | **0.268** |
| T2 | **171(10.8%)** | **85(11.0%)** | **47(13.0%)** | **39(8.7%)** |  |
| T3 | **218(13.8%)** | **100(13.0%)** | **55(15.2%)** | **63(14.1%)** |  |
| T4 | **1191(75.4%)** | **585(76.0%)** | **260(71.8%)** | **346(77.2%)** |  |
| **pN stage** |  |  |  |  | **0.426** |
| N0 | **443(28.0%)** | **227(29.5%)** | **94(26.0%)** | **122(27.2%)** |  |
| N+ | **1137(72.0%)** | **543(70.5%)** | **268(74.0%)** | **326(72.8%)** |  |
| **Primary site** |  |  |  |  | **0.293** |
| Up 1/3 | **521(33.0%)** | **252(32.7%)** | **119(32.9%)** | **150(33.5%)** |  |
| Middle 1/3 | **230(14.6%)** | **124(16.1%)** | **41(11.3%)** | **65(14.5%)** |  |
| Lower 1/3 | **829(52.5%)** | **394(51.2%)** | **202(55.8%)** | **233(52.0%)** |  |
| **Tumor size, cm** |  |  |  |  | **0.273** |
| ≤5 | **839(53.1%)** | **405(52.6%)** | **205(56.6%)** | **229(51.1%)** |  |
| ＞5 | **741(46.9%)** | **365(47.4%)** | **157(43.4%)** | **219(48.9%)** |  |
| **Histology** |  |  |  |  | **0.346** |
| None/Low | **1237(78.3%)** | **593(77.0%)** | **283(78.2%)** | **361(80.6%)** |  |
| High/Median | **343(21.7%)** | **177(23.0%)** | **79(21.8%)** | **87(19.4%)** |  |
| **Chemotherapy** |  |  |  |  | **0.432** |
| Yes | **1304(82.5%)** | **627(81.4%)** | **306(84.5%)** | **371(82.8%)** |  |
| No | **276(17.5%)** | **143(18.6%)** | **56(15.5%)** | **77(17.2%)** |  |
| **SII^*^** |  |  |  |  | **0.009** |
| Low | **911(57.7%)** | **465(60.4%)** | **184(50.8%)** | **262(58.5%)** |  |
| High | **669(42.3%)** | **305(39.6%)** | **178(49.2%)** | **186(41.5%)** |  |
| **PNI^*^** |  |  |  |  | **0.762** |
| Low | **343(21.7%)** | **172(22.3%)** | **79(21.8%)** | **92(20.5%)** |  |
| High | **1237(78.3%)** | **598(77.7%)** | **283(78.2%)** | **356(79.5%)** |  |
| **NLR^*^** |  |  |  |  | **0.707** |
| Low | **958(60.6%)** | **473(61.4%)** | **213(58.8%)** | **272(60.7%)** |  |
| High | **622(39.4%)** | **297(38.6%)** | **149(41.2%)** | **176(39.3%)** |  |
| **PLR^*^** |  |  |  |  | **0.208** |
| Low | **722(45.7%)** | **361(46.9%)** | **172(47.5%)** | **189(42.2%)** |  |
| High | **858(54.3%)** | **409(53.1%)** | **190(52.5%)** | **259(57.8%)** |  |
| **Recurrence** |  |  |  |  | **0.461** |
| Yes | **843(53.4%)** | **402(52.2%)** | **191(52.8%)** | **250(55.8%)** |  |
| No | **737(46.6%)** | **368(47.8%)** | **171(47.2%)** | **198(44.2%)** |  |

**Note:** ECOG PS = Eastern Cooperative Oncology Group Performance Status; SII = Systemic Immune-inflammation Index; PNI = Prognostic Nutritional Index; NLR = Neutrophil to Lymphocyte Ratio; PLR = Platelet to Lymphpcyte Ratio. * Divided into high and low groups using the median as the critical value.

**Table S6. Clinical characteristics of patients according to the recurrence status in the training internal and external validation cohorts [n(%)].**

| **Variables** | **Training cohort  (n=770)** | | ***P* value** | **Internal validation cohort (n=362)** | | ***P* value** | **External validation cohort (n=448)** | | ***P* value** |
| --- | --- | --- | --- | --- | --- | --- | --- | --- | --- |
|  | **Non-Recur (N=368)** | **Recur**  **(N=402)** |  | **Non-Recur**  **(N=171)** | **Recur (N=191)** |  | **Non-Recur**  **(N=198)** | **Recur**  **(N=250)** |  |
| **Gender** |  |  | **0.342** |  |  | **0.274** |  |  | **0.327** |
| Male | **238(64.7%)** | **273(67.9%)** |  | **109(63.7%)** | **111(58.1%)** |  | **130(65.7%)** | **175(70.0%)** |  |
| Female | **130(35.3%)** | **129(32.1%)** |  | **62(36.3%)** | **80(41.9%)** |  | **68(34.3%)** | **75(30.0%)** |  |
| **Age, years** |  |  | **0.591** |  |  | **0.482** |  |  | **0.922** |
| ≤65 | **165(44.8%)** | **188(46.8%)** |  | **85(49.7%)** | **102(53.4%)** |  | **87(43.9%)** | **111(44.4%)** |  |
| ＞65 | **203(55.2%)** | **214(53.2%)** |  | **86(50.3%)** | **89(46.6%)** |  | **111(56.1%)** | **139(55.6%)** |  |
| **ECOG PS** |  |  | **0.071** |  |  | **0.073** |  |  | **0.614** |
| 0-1 | **186(50.5%)** | **177(44.0%)** |  | **102(59.6%)** | **96(50.3%)** |  | **99(50.0%)** | **119(47.6%)** |  |
| 2 | **182(49.5%)** | **225(56.0%)** |  | **69(44.4%)** | **95(49.7%)** |  | **99(50.0%)** | **131(52.4%)** |  |
| **T stage** |  |  | **0.000** |  |  | **0.000** |  |  | **0.000** |
| T2/T3 | **132(35.9%)** | **53(13.2%)** |  | **71(41.5%)** | **31(16.2%)** |  | **67(33.8%)** | **35(14.0%)** |  |
| T4 | **236(64.1%)** | **349(86.8%)** |  | **100(58.5%)** | **160(83.8%)** |  | **131(66.2%)** | **215(86.0%)** |  |
| **N stage** |  |  | **0.000** |  |  | **0.000** |  |  | **0.000** |
| N0 | **175(47.6%)** | **52(12.9%)** |  | **69(40.4%)** | **25(13.1%)** |  | **85(42.9%)** | **37(14.8%)** |  |
| N+ | **193(52.4%)** | **350(82.1%)** |  | **102(59.6%)** | **166(86.9%)** |  | **113(57.1%)** | **213(85.2%)** |  |
| **Primary site** |  |  | **0.150** |  |  | **0.822** |  |  | **0.564** |
| Up 1/3 | **133(36.1%)** | **119(29.6%)** |  | **59(34.5%)** | **60(31.4%)** |  | **66(33.3%)** | **84(33.6%)** |  |
| Middle 1/3 | **55(14.9%)** | **69(17.2%)** |  | **19(11.1%)** | **22(11.5%)** |  | **25(12.6%)** | **40(16.0%)** |  |
| Lower 1/3 | **180(48.9%)** | **214(53.2%)** |  | **93(54.4%)** | **109(57.1%)** |  | **107(54.0%)** | **126(50.4%)** |  |
| **Tumor size, cm** |  |  | **0.131** |  |  | **0.018** |  |  | **0.362** |
| ≤5 | **204(55.4%)** | **201(50.0%)** |  | **108(63.2%)** | **97(50.8%)** |  | **106(53.5%)** | **123(49.2%)** |  |
| ＞5 | **164(44.6%)** | **201(50.0%)** |  | **63(36.8%)** | **94(49.2%)** |  | **92(46.5%)** | **127(50.8%)** |  |
| **Histology** |  |  | **0.000** |  |  | **0.000** |  |  | **0.000** |
| None/Low | **259(70.4%)** | **334(83.1%)** |  | **116(67.8%)** | **167(87.4%)** |  | **138(69.7%)** | **223(89.2%)** |  |
| High/Median | **109(29.6%)** | **68(16.9%)** |  | **55(32.2%)** | **24(12.6%)** |  | **60(30.3%)** | **27(10.8%)** |  |
| **SII^*^** |  |  | **0.482** |  |  | **0.056** |  |  | **0.536** |
| Low | **227(61.7%)** | **238(59.2%)** |  | **96(56.1%)** | **88(46.1%)** |  | **119(60.1%)** | **143(57.2%)** |  |
| High | **141(38.3%)** | **164(40.8%)** |  | **75(43.9%)** | **103(53.9%)** |  | **79(39.9%)** | **107(42.8%)** |  |
| **PNI^*^** |  |  | **0.579** |  |  | **0.107** |  |  | **0.752** |
| Low | **79(21.5%)** | **93(23.1%)** |  | **31(18.1%)** | **48(25.1%)** |  | **42(21.2%)** | **50(20.0%)** |  |
| High | **289(78.5%)** | **309(76.9%)** |  | **140(81.9%)** | **143(74.9%)** |  | **156(78.8%)** | **200(80.0%)** |  |
| **NLR^*^** |  |  | **0.548** |  |  | **0.323** |  |  | **0.310** |
| Low | **222(60.3%)** | **251(62.4%)** |  | **96(56.1%)** | **117(61.3%)** |  | **115(58.1%)** | **157(62.8%)** |  |
| High | **146(39.7%)** | **151(37.6%)** |  | **75(43.9%)** | **74(38.7%)** |  | **83(48.9%)** | **93(37.2%)** |  |
| **PLR^*^** |  |  | **0.005** |  |  | **0.003** |  |  | **0.016** |
| Low | **153(41.6%)** | **208(51.7%)** |  | **67(39.2%)** | **105(55.0%)** |  | **71(35.9%)** | **118(47.2%)** |  |
| High | **215(58.4%)** | **194(48.3%)** |  | **104(60.8%)** | **86(45.0%)** |  | **127(64.1%)** | **132(52.8%)** |  |

**Note:** ECOG PS = Eastern Cooperative Oncology Group Performance Status; SII = Systemic Immune-inflammation Index; PNI = Prognostic Nutritional Index; NLR = Neutrophil to Lymphocyte Ratio; PLR = Platelet to Lymphpcyte Ratio. * Divided into high and low groups using the median as the critical value.

**Table S7. Univariate and multivariate logistic regression analysis of factors affecting postoperative recurrence in patients with LAGC in the training set**

| **Variable** | **Univariate logistic regression** | |  | **Multivariate logistic regression** | |
| --- | --- | --- | --- | --- | --- |
|  | **OR (95%CI)** | ***P* value** |  | **OR (95%CI)** | ***P* value** |
| **Gender** |  | **0.545** |  |  |  |
| Male | **Reference** |  |  |  |  |
| Female | **0.882 (0.587-1.325)** |  |  |  |  |
| **Age, years** |  | **0.482** |  |  |  |
| ≤65 | **Reference** |  |  |  |  |
| ＞65 | **1.152 (0.777-1.708)** |  |  |  |  |
| **ECOG PS** |  | **0.556** |  |  |  |
| 0-1 | **Reference** |  |  |  |  |
| 2 | **1.124 (0.761-1.660)** |  |  |  |  |
| **T stage** |  | **0.014** |  |  | **0.012** |
| T2/T3 | **Reference** |  |  | **Reference** |  |
| T4 | **1.783 (1.127-2.822)** |  |  | **1.789 (1.136-2.816)** |  |
| **N stage** |  | **＜0.001** |  |  | **＜0.001** |
| N0 | **Reference** |  |  | **Reference** |  |
| N+ | **2.857 (1.842-4.433)** |  |  | **2.886 (1.870-4.453)** |  |
| **Primary site** |  | **0.853** |  |  |  |
| Up 1/3 | **Reference** |  |  |  |  |
| Middle 1/3 | **1.061 (0.684-1.648)** | **0.791** |  |  |  |
| Lower 1/3 | **1.170 (0.672-2.036)** | **0.578** |  |  |  |
| **Tumor size, cm** |  | **0.108** |  |  |  |
| ≤5 | **Reference** |  |  |  |  |
| ＞5 | **1.384 (0.931-2.057)** |  |  |  |  |
| **Histology** |  | **0.007** |  |  | **0.011** |
| High/Median | **Reference** |  |  | **Reference** |  |
| None/Low | **1.720 (1.162-2.546)** |  |  | **1.646 (1.120-2.419)** |  |
| **SII^*^** |  | **0.060** |  |  |  |
| Low | **Reference** |  |  |  |  |
| High | **1.706 (0.978-2.977)** |  |  |  |  |
| **PNI^*^** |  | **0.374** |  |  |  |
| Low | **Reference** |  |  |  |  |
| High | **0.806 (0.501-1.296)** |  |  |  |  |
| **NLR^*^** |  | **0.081** |  |  |  |
| High | **Reference** |  |  |  |  |
| Low | **0.625 (0.369-1.059)** |  |  |  |  |
| **PLR^*^** |  | **0.029** |  |  | **0.054** |
| High | **Reference** |  |  | **Reference** |  |
| Low | **0.602 (0.381-0.951)** |  |  | **0.673 (0.458-1.089)** |  |
| **Radiomics score** |  | **＜0.001** |  |  | **＜0.001** |
| Low | **Reference** |  |  | **Reference** |  |
| High | **7.936 (5.140-12.254)** |  |  | **7.871 (5.139-12.054)** |  |
| **Pathomic score** |  | **＜0.001** |  |  | **＜0.001** |
| Low | **Reference** |  |  | **Reference** |  |
| High | **7.390 (4.955-11.022)** |  |  | **7.193 (4.852-10.662)** |  |

**Note:** ECOG PS = Eastern Cooperative Oncology Group Performance Status; SII = Systemic Immune-inflammation Index; PNI = Prognostic Nutritional Index; NLR = Neutrophil to Lymphocyte Ratio; PLR = Platelet to Lymphpcyte Ratio. * Divided into high and low groups using the median as the critical value.

**Table S8. Comparison of performance indicators of different models in predicting postoperative recurrence in patients with LAGC in training set**

| **Variable** | **AUC** | **Accuracy** | **Sensitivity** | **Specificity** | **PPV** | **NPV** | **PLR (x10)** | **NLR** | **F1 score** |
| --- | --- | --- | --- | --- | --- | --- | --- | --- | --- |
| Radiomics | **0.806** | **0.721** | **0.716** | **0.726** | **0.740** | **0.701** | **0.391** | **0.728** | **0.261** |
| Pathomics | **0.820** | **0.756** | **0.759** | **0.753** | **0.770** | **0.741** | **0.321** | **0.764** | **0.307** |
| Clinical features | **0.756** | **0.708** | **0.769** | **0.641** | **0.701** | **0.717** | **0.361** | **0.733** | **0.214** |
| RSA model | **0.903** | **0.827** | **0.811** | **0.845** | **0.851** | **0.804** | **0.224** | **0.831** | **0.524** |

Note: AUC-area under the curve; PPV-Positive Predictive Value; NVP-Negative Predictive Value; PLR-Positive Likelihood Ratio; NLR-Negative Likelihood Ratio.

**Table S9. Proportion of different models in predicting postoperative recurrence in LAGC patients**

| **Sequence** | **Clinical features vs. RSA model** | **Radiomics vs. RSA model** | **Pathomics vs. RSA models** |
| --- | --- | --- | --- |
| **Training set** |  |  |  |
| **NRI (95%CI)** | **0.492 (0.365-0.621)** | **0.339 (0.200-0.455)** | **0.289 (0.168-0.409)** |
| **IDI (95%CI)** | **0.294 (0.261-0.390)** | **0.227 (0.200-0.256)** | **0.198 (0.170-0.225)** |
| **DeLong Test** | **＜0.001** | **＜0.001** | **＜0.001** |
| **Internal validation set 1** |  |  |  |
| **NRI (95%CI)** | **0.662 (0.391-0.934)** | **0.393 (0.139-0.656)** | **0.394 (0..151-0.644)** |
| **IDI (95%CI)** | **0.318 (0.255-0.378)** | **0.225 (0.166-0.282)** | **0.245 (0.183-0.307)** |
| **DeLong Test** | **＜0.001** | **＜0.001** | **＜0.001** |
| **Internal validation set 2** |  |  |  |
| **NRI (95%CI)** | **0.674 (0.407-0.952)** | **0.463 (0.168-0.764)** | **0.342 (0.119-0.591)** |
| **IDI (95%CI)** | **0.299 (0.229-0.368)** | **0.250 (0.185-0.312)** | **0.223 (0.163-0.283)** |
| **DeLong Test** | **0.0005** | **0.0002** | **＜0.001** |
| **External validation set 1** |  |  |  |
| **NRI (95%CI)** | **0.622 (0.349-0.913)** | **0.314 (0.116-0.511)** | **0.359 (0.179-0.552)** |
| **IDI (95%CI)** | **0.275 (0.215-0.336)** | **0.239 (0.183-0.289)** | **0.177 (0.121-0.230)** |
| **DeLong Test** | **＜0.001** | **＜0.001** | **＜0.001** |
| **External validation set 2** |  |  |  |
| **NRI (95%CI)** | **0.558 (0.264-0.845)** | **0.581 (0.295-0.850)** | **0.285 (0.043-0.542)** |
| **IDI (95%CI)** | **0.306 (0.241-0.369)** | **0.279 (0.214-0.343)** | **0.166 (0.102-0.228)** |
| **DeLong Test** | **＜0.001** | **＜0.001** | **＜0.001** |
| **Prospective cohort**  **initial surgery group** |  |  |  |
| **NRI (95%CI)** | **0.598 (0.211-0.973)** | **0.306 (-0.100-0.705)** | **0.254 (-0.114-0.617)** |
| **IDI (95%CI)** | **0.302 (0.202-0.398)** | **0.210 (0.124-0.298)** | **0.170 (0.077-0.262)** |
| **DeLong Test** | **＜0.001** | **0.009** | **0.011** |
| **Prospective cohort**  **neoadjuvant chemotherapy group** |  |  |  |
| **NRI (95%CI)** | **0.429 (0.110-0.774)** | **0.108 (-0.136-0.356)** | **0.137 (-0.116-0.396)** |
| **IDI (95%CI)** | **0.196 (0.121-0.274)** | **0.133 (0.068-0.196)** | **0.128 (0.059-0.202)** |
| **DeLong Test** | **0.003** | **0.070** | **0.134** |

**Table S10. Univariate and multivariate Cox regression analysis of the factors affecting 5-year overall survival in patients with LAGC after surgery in the training set.**

| **Variable** | **Univariate analysis** | |  | **Multivariate analysis** | |
| --- | --- | --- | --- | --- | --- |
|  | **HR (95%CI)** | ***P* value** |  | **HR (95%CI)** | ***P* value** |
| **Gender** |  | **0.129** |  |  |  |
| Male | **Reference** |  |  |  |  |
| Female | **0.843 (0.676-1.051)** |  |  |  |  |
| **Age, years** |  | **0.062** |  |  |  |
| ≤65 | **Reference** |  |  |  |  |
| ＞65 | **0.822 (0.669-1.010)** |  |  |  |  |
| **ECOG PS** |  | **0.420** |  |  |  |
| 0-1 | **Reference** |  |  |  |  |
| 2 | **0.919 (0.750-1.128)** |  |  |  |  |
| **T stage** |  | **0.018** |  |  | **0.011** |
| T2/T3 | **Reference** |  |  | **Reference** |  |
| T4 | **1.989 (1.556-2.295)** |  |  | **1.961 (1.736-2.255)** |  |
| **N stage** |  | **0.011** |  |  | **0.010** |
| N0 | **Reference** |  |  | **Reference** |  |
| N+ | **2.291 (1.878-2.704)** |  |  | **2.251 (1.951-2.645)** |  |
| **Primary site** |  | **0.469** |  |  |  |
| Up 1/3 | **Reference** |  |  |  |  |
| Middle 1/3 | **1.009 (0.800-1.273)** | **0.940** |  |  |  |
| Lower 1/3 | **1.187 (0.894-1.577)** | **0.235** |  |  |  |
| **Tumor size, cm** |  | **0.288** |  |  |  |
| ≤5 | **Reference** |  |  |  |  |
| ＞5 | **0.893 (0.726-1.100)** |  |  |  |  |
| **Histology** |  | **0.018** |  |  | **0.340** |
| High/Median | **Reference** |  |  | **Reference** |  |
| None/Low | **1.900 (1.576-2.295)** |  |  | **0.905 (0.737-1.111)** |  |
| **SII^*^** |  | **0.591** |  |  |  |
| Low | **Reference** |  |  |  |  |
| High | **0.927 (0.704-1.221)** |  |  |  |  |
| **PNI^*^** |  | **0.526** |  |  |  |
| Low | **Reference** |  |  |  |  |
| High | **1.087 (0.841-1.404)** |  |  |  |  |
| **NLR^*^** |  | **0.150** |  |  |  |
| Low | **Reference** |  |  |  |  |
| High | **1.215 (0.932-1.584)** |  |  |  |  |
| **PLR^*^** |  | **0.985** |  |  |  |
| Low | **Reference** |  |  |  |  |
| High | **1.002 (0.795-1.263)** |  |  |  |  |
| **Recurrence** |  | **0.013** |  |  | **0.017** |
| No | **Reference** |  |  | **Reference** |  |
| Yes | **2.035 (1.777-2.380)** |  |  | **2.001 (1.753-2.330)** |  |
| **Radiomics score** |  | **0.025** |  |  | **0.016** |
| Low | **Reference** |  |  | **Reference** |  |
| High | **1.955 (1.713-2.279)** |  |  | **1.948 (1.710-2.265)** |  |
| **Pathomic score** |  | **0.008** |  |  | **0.009** |
| Low | **Reference** |  |  | **Reference** |  |
| High | **2.108 (1.839-2.464)** |  |  | **2.106 (1.837-2.462)** |  |
| **RSA model score** |  |  |  |  | **0.004** |
| Low | **Reference** | **0.006** |  | **Reference** |  |
| High | **2.497 (2.027-3.181)** |  |  | **2.598 (2.101-3.319)** |  |

**Note:** ECOG PS = Eastern Cooperative Oncology Group Performance Status; SII = Systemic Immune-inflammation Index; PNI = Prognostic Nutritional Index; NLR = Neutrophil to Lymphocyte Ratio; PLR = Platelet to Lymphpcyte Ratio. * Divided into high and low groups using the median as the critical value.

**Table S11. Comparison of performance indicators of different models for predicting postoperative recurrence of LAGC patients in the training set based on different expressions of molecular marker HER2 and peripheral blood tumor markers.**

| **Variable** | **AUC** | **Accuracy** | **Sensitivity** | **Specificity** | **PPV** | **NPV** | **PLR (x10)** | **NLR** | **F1 score** |
| --- | --- | --- | --- | --- | --- | --- | --- | --- | --- |
| **HER2 positive** |  |  |  |  |  |  |  |  |  |
| Clinical features | **0.834** | **0.810** | **0.878** | **0.737** | **0.783** | **0.848** | **0.166** | **0.828** | **0.334** |
| Radiomics | **0.821** | **0.797** | **0.854** | **0.737** | **0.778** | **0.824** | **0.199** | **0.814** | **0.324** |
| Pathomics | **0.765** | **0.747** | **0.854** | **0.632** | **0.714** | **0.800** | **0.232** | **0.778** | **0.232** |
| RSA model | **0.954** | **0.899** | **0.878** | **0.921** | **0.923** | **0.875** | **0.132** | **0.900** | **1.112** |
| **HER2 negative** |  |  |  |  |  |  |  |  |  |
| Clinical features | **0.781** | **0.699** | **0.744** | **0.648** | **0.706** | **0.691** | **0.395** | **0.724** | **0.211** |
| Radiomics | **0.809** | **0.745** | **0.758** | **0.731** | **0.761** | **0.727** | **0.331** | **0.760** | **0.281** |
| Pathomics | **0.751** | **0.682** | **0.721** | **0.637** | **0.693** | **0.668** | **0.437** | **0.707** | **0.199** |
| RSA model | **0.880** | **0.801** | **0.845** | **0.751** | **0.794** | **0.810** | **0.207** | **0.819** | **0.340** |
| **Tumor marker positive** |  |  |  |  |  |  |  |  |  |
| Clinical features | **0.812** | **0.717** | **0.715** | **0.719** | **0.740** | **0.692** | **0.396** | **0.728** | **0.254** |
| Radiomics | **0.813** | **0.750** | **0.768** | **0.729** | **0.761** | **0.737** | **0.318** | **0.764** | **0.283** |
| Pathomics | **0.765** | **0.709** | **0.771** | **0.639** | **0.705** | **0.713** | **0.359** | **0.737** | **0.213** |
| RSA model | **0.910** | **0.833** | **0.805** | **0.865** | **0.870** | **0.798** | **0.226** | **0.836** | **0.594** |
| **Tumor marker negative** |  |  |  |  |  |  |  |  |  |
| Clinical features | **0.780** | **0.736** | **0.759** | **0.713** | **0.723** | **0.750** | **0.338** | **0.741** | **0.264** |
| Radiomics | **0.847** | **0.799** | **0.797** | **0.800** | **0.797** | **0.800** | **0.253** | **0.797** | **0.399** |
| Pathomics | **0.727** | **0.704** | **0.759** | **0.650** | **0.682** | **0.732** | **0.370** | **0.719** | **0.217** |
| RSA model | **0.884** | **0.843** | **0.873** | **0.813** | **0.821** | **0.867** | **0.156** | **0.847** | **0.466** |

Note: AUC-area under the curve; PPV-Positive Predictive Value; NVP-Negative Predictive Value; PLR-Positive Likelihood Ratio; NLR-Negative Likelihood Ratio.

**Table S12. Baseline characteristics of patients in the training set were stratified by adjuvant chemotherapy received before and after PSM.**

| **Variable** | **Before PSM** | | ***P* value** | **After PSM** | | ***P* value** |
| --- | --- | --- | --- | --- | --- | --- |
|  | **Chemotherapy**  **(N=627)** | **Non-Chemotherapy**  **(N=143)** |  | **Chemotherapy**  **(N=108)** | **Non-Chemotherapy**  **(N=108)** |  |
| **Gender** |  |  | **0.001** |  |  | **0.889** |
| Male | **433(69.1%)** | **78(54.5%)** |  | **65(60.2%)** | **66(61.1%)** |  |
| Female | **194(30.9%)** | **65(45.5%)** |  | **43(39.8%)** | **42(38.9%)** |  |
| **Age, years** |  |  | **0.917** |  |  | **0.783** |
| ≤65 | **288(45.9%)** | **65(45.5%)** |  | **44(40.7%)** | **46(42.6%)** |  |
| ＞65 | **339(54.1%)** | **78(54.5%)** |  | **64(59.3%)** | **62(57.4%)** |  |
| **ECOG PS** |  |  | **0.118** |  |  | **0.683** |
| 0-1 | **304(48.5%)** | **59(41.3%)** |  | **50(46.3%)** | **53(49.1%)** |  |
| 2 | **323(51.5%)** | **84(58.7%** |  | **58(53.7%)** | **55(50.9%)** |  |
| **T stage** |  |  | **0.001** |  |  | **1.000** |
| T2/T3 | **136(21.7%)** | **49(34.3%)** |  | **23(21.3%)** | **23(21.3%)** |  |
| T4 | **491(78.3%)** | **94(65.7%)** |  | **85(78.7%)** | **85(78.7%)** |  |
| **N stage** |  |  | **0.009** |  |  | **0.650** |
| N0 | **172(27.4%)** | **55(38.5%)** |  | **29(26.9%)** | **32(29.6%)** |  |
| N+ | **455(72.6%)** | **88(61.5%)** |  | **79(73.1%)** | **76(70.4%)** |  |
| **Primary site** |  |  | **0.076** |  |  | **0.177** |
| Up 1/3 | **198(31.6%)** | **54(37.8%)** |  | **30(27.8%)** | **40(37.0%)** |  |
| Middle 1/3 | **96(15.3%)** | **28(19.6%)** |  | **20(18.5%)** | **12(11.1%)** |  |
| Lower 1/3 | **333(53.1%)** | **61(42.7%)** |  | **58(53.7%)** | **56(51.9%)** |  |
| **Tumor size, cm** |  |  | **0.018** |  |  | **0.218** |
| ≤5 | **317(50.6%)** | **88(61.5%)** |  | **55(50.9%)** | **64(59.3%)** |  |
| ＞5 | **310(49.4%)** | **55(38.5%)** |  | **53(49.1%)** | **44(40.7%)** |  |
| **Histology** |  |  | **0.014** |  |  | **0.513** |
| None/Low | **494(78.8%)** | **99(69.2%)** |  | **82(75.9%)** | **86(79.6%)** |  |
| High/Median | **133(21.2%)** | **44(30.8%)** |  | **26(24.1%)** | **22(20.4%)** |  |
| **SII^*^** |  |  | **0.076** |  |  | **0.264** |
| Low | **388(61.9%)** | **77(53.8%)** |  | **70(64.8%)** | **62(57.4%)** |  |
| High | **239(38.1%)** | **66(46.2%)** |  | **38(35.2%)** | **46(42.6%)** |  |
| **PNI^*^** |  |  | **0.367** |  |  | **0.333** |
| Low | **136(21.7%)** | **36(25.2%)** |  | **28(25.9%)** | **22(20.4%)** |  |
| High | **491(78.3%)** | **107(74.8%)** |  | **80(74.1%)** | **86(79.6%)** |  |
| **NLR^*^** |  |  | **0.061** |  |  | **0.780** |
| Low | **395(63.0%)** | **78(54.5%)** |  | **67(62.0%)** | **65(60.2%)** |  |
| High | **232(37.0%)** | **65(45.5%)** |  | **41(38.0%)** | **43(39.8%)** |  |
| **PLR^*^** |  |  | **0.994** |  |  | **0.785** |
| Low | **294(46.9%)** | **67(46.9%)** |  | **52(48.1%)** | **50(46.3%)** |  |
| High | **333(53.1%)** | **76(53.1%)** |  | **56(51.9%)** | **58(53.7%)** |  |

**Note:** ECOG PS = Eastern Cooperative Oncology Group Performance Status; SII = Systemic Immune-inflammation Index; PNI = Prognostic Nutritional Index; NLR = Neutrophil to Lymphocyte Ratio; PLR = Platelet to Lymphpcyte Ratio. * Divided into high and low groups using the median as the critical value.

**Table S13. Univariate and multivariate analyses of overall survival were performed for patients with an RSA model score of 0.19 or greater in the matched training cohort.**

| **Variable** | **Univariate analysis** | |  | **Multivariate analysis** | |
| --- | --- | --- | --- | --- | --- |
|  | **HR (95%CI)** | ***P* value** |  | **HR (95%CI)** | ***P* value** |
| **Gender** |  | **0.910** |  |  |  |
| Male | **Reference** |  |  |  |  |
| Female | **0.975 (0.630-1.508)** |  |  |  |  |
| **Age, years** |  | **0.303** |  |  |  |
| ≤65 | **Reference** |  |  |  |  |
| ＞65 | **0.793 (0.509-1.234)** |  |  |  |  |
| **ECOG PS** |  | **0.846** |  |  |  |
| 0-1 | **Reference** |  |  |  |  |
| 2 | **1.044 (0.676-1.613)** |  |  |  |  |
| **T stage** |  | **0.022** |  |  | **0.017** |
| T2/T3 | **Reference** |  |  | **Reference** |  |
| T4 | **3.427 (1.198-9.801)** |  |  | **3.373 (1.238-9.191)** |  |
| **N stage** |  | **0.713** |  |  |  |
| N0 | **Reference** |  |  |  |  |
| N+ | **1.133 (0.583-2.202)** |  |  |  |  |
| **Primary site** |  | **0.389** |  |  |  |
| Up 1/3 | **Reference** |  |  |  |  |
| Middle 1/3 | **1.361 (0.848-2.182)** | **0.201** |  |  |  |
| Lower 1/3 | **1.340 (0.701-2.563)** | **0.376** |  |  |  |
| **Tumor size, cm** |  | **0.545** |  |  |  |
| ≤5 | **Reference** |  |  |  |  |
| ＞5 | **0.872 (0.560-1.358)** |  |  |  |  |
| **Histology** |  | **0.745** |  |  |  |
| High/Median | **Reference** |  |  |  |  |
| None/Low | **0.930 (0.602-1.438)** |  |  |  |  |
| **Chemotherapy** |  | **＜0.001** |  |  | **0.001** |
| Yes | **Reference** |  |  | **Reference** |  |
| No | **2.198 (1.427-3.384)** |  |  | **2.087 (1.375-3.166)** |  |
| **SII^*^** |  | **0.158** |  |  |  |
| High | **Reference** |  |  |  |  |
| Low | **0.645 (0.351-1.186)** |  |  |  |  |
| **PNI^*^** |  | **0.919** |  |  |  |
| Low | **Reference** |  |  |  |  |
| High | **0.971 (0.555-1.700)** |  |  |  |  |
| **NLR^*^** |  | **0.191** |  |  |  |
| Low | **Reference** |  |  |  |  |
| High | **1.512 (0.814-2.810)** |  |  |  |  |
| **PLR^*^** |  | **0.205** |  |  |  |
| High | **Reference** |  |  |  |  |
| Low | **0.736 (0.458-1.183)** |  |  |  |  |

**Note:** ECOG PS = Eastern Cooperative Oncology Group Performance Status; SII = Systemic Immune-inflammation Index; PNI = Prognostic Nutritional Index; NLR = Neutrophil to Lymphocyte Ratio; PLR = Platelet to Lymphpcyte Ratio. * Divided into high and low groups using the median as the critical value

**Table S14. Comparison of clinical characteristics of LAGC patients admitted in different time periods in the internal validation set.**

| **Variable** | **Total**  **(N=362)** | **Internal validation-1 (2012-2014-N=194)** | **Internal validation-2 (2017-2019-N=168)** | ***P* value** |
| --- | --- | --- | --- | --- |
| **Gender** |  |  |  | **0.650** |
| Male | **220(60.8%)** | **120(61.9%)** | **100(59.5%)** |  |
| Female | **142(39.2%)** | **74(38.1%)** | **68(40.5%)** |  |
| **Age, years** |  |  |  | **0.640** |
| ≤65 | **187(51.7%)** | **98(50.5%)** | **89(53.0%)** |  |
| ＞65 | **175(48.3%)** | **96(49.5%)** | **79(47.0%)** |  |
| **ECOG PS** |  |  |  | **0.814** |
| 0-1 | **198(54.7%)** | **105(54.1%)** | **93(55.4%)** |  |
| 2 | **164(45.3%)** | **89(45.9%)** | **75(44.6%)** |  |
| **T stage** |  |  |  | **0.877** |
| T2/T3 | **102(28.2%)** | **54(27.8%)** | **48(28.6%)** |  |
| T4 | **260(71.8%)** | **140(72.2%)** | **120(71.4%)** |  |
| **N stage** |  |  |  | **0.311** |
| N0 | **94(24.6%)** | **52(26.8%)** | **42(25.0%)** |  |
| N+ | **268(75.4%)** | **142(73.2%)** | **126(75.0%)** |  |
| **Primary site** |  |  |  | **0.786** |
| Up 1/3 | **119(32.9%)** | **66(34.0%)** | **53(31.5%)** |  |
| Middle 1/3 | **41(11.3%)** | **23(11.9%)** | **18(10.7%)** |  |
| Lower 1/3 | **202(55.8%)** | **105(54.1%)** | **97(57.7%)** |  |
| **Tumor size, cm** |  |  |  | **0.855** |
| ≤5 | **205(56.6%)** | **109(56.2%)** | **96(57.1%)** |  |
| ＞5 | **157(43.4%)** | **85(43.8%)** | **72(42.9%)** |  |
| **Histology** |  |  |  | **0.931** |
| None/Low | **283(78.2%)** | **152(78.4%)** | **131(78.0%)** |  |
| High/Median | **79(21.8%)** | **42(21.6%)** | **37(22.0%)** |  |
| **SII^*^** |  |  |  | **0.583** |
| Low | **184(50.8%)** | **96(49.5%)** | **88(52.4%)** |  |
| High | **178(49.2%)** | **98(50.5%)** | **80(47.6%)** |  |
| **PNI^*^** |  |  |  | **0.551** |
| Low | **79(21.8%)** | **40(20.6%)** | **39(23.2%)** |  |
| High | **283(78.2%)** | **154(79.4%)** | **129(76.8%)** |  |
| **NLR^*^** |  |  |  | **0.855** |
| Low | **213(58.8%)** | **115(59.3%)** | **98(58.3%)** |  |
| High | **149(41.2%)** | **79(40.7%)** | **70(41.7%)** |  |
| **PLR^*^** |  |  |  | **0.646** |
| Low | **172(47.5%)** | **90(46.4%)** | **82(48.8%)** |  |
| High | **190(52.5%)** | **104(53.6%)** | **86(51.2%)** |  |

**Note:** ECOG PS = Eastern Cooperative Oncology Group Performance Status; SII = Systemic Immune-inflammation Index; PNI = Prognostic Nutritional Index; NLR = Neutrophil to Lymphocyte Ratio; PLR = Platelet to Lymphpcyte Ratio. * Divided into high and low groups using the median as the critical value.

**Table S15. Comparison of performance indicators of different models in predicting postoperative recurrence in patients with LAGC in internal validation set.**

| **Variable** | **AUC** | **Accuracy** | **Sensitivity** | **Specificity** | **PPV** | **NPV** | **PLR (x10)** | **NLR** | **F1 score** |
| --- | --- | --- | --- | --- | --- | --- | --- | --- | --- |
| **Internal validation set -1 (2012-2014)** |  |  |  |  |  |  |  |  |  |
| Clinical features | **0.798** | **0.711** | **0.703** | **0.720** | **0.732** | **0.691** | **0.412** | **0.717** | **0.251** |
| Radiomics | **0.792** | **0.732** | **0.733** | **0.731** | **0.747** | **0.716** | **0.366** | **0.740** | **0.273** |
| Pathomics | **0.740** | **0.686** | **0.723** | **0.645** | **0.689** | **0.682** | **0.430** | **0.705** | **0.204** |
| RSA model | **0.902** | **0.830** | **0.812** | **0.849** | **0.854** | **0.806** | **0.221** | **0.832** | **0.539** |
| **Internal validation set-2 (2017-2019)** |  |  |  |  |  |  |  |  |  |
| Clinical features | **0.774** | **0.720** | **0.733** | **0.705** | **0.742** | **0.696** | **0.379** | **0.737** | **0.249** |
| Radiomics | **0.790** | **0.750** | **0.767** | **0.731** | **0.767** | **0.731** | **0.319** | **0.767** | **0.285** |
| Pathomics | **0.734** | **0.685** | **0.733** | **0.628** | **0.695** | **0.671** | **0.424** | **0.714** | **0.197** |
| RSA model | **0.892** | **0.851** | **0.878** | **0.821** | **0.850** | **0.853** | **0.149** | **0.863** | **0.489** |

Note: AUC-area under the curve; PPV-Positive Predictive Value; NVP-Negative Predictive Value; PLR-Positive Likelihood Ratio; NLR-Negative Likelihood Ratio.

**Table S16. Univariate and multivariate Cox regression analysis of the factors affecting the 5-year overall survival rate of LAGC patients after surgery in the internal validation set -1 (2012-2014).**

| **Variable** | **Univariate analysis** | |  | **Multivariate analysis** | |
| --- | --- | --- | --- | --- | --- |
|  | **HR (95%CI)** | ***P* value** |  | **HR (95%CI)** | ***P* value** |
| **Gender** |  | **0.987** |  |  |  |
| Male | **Reference** |  |  |  |  |
| Female | **1.004 (0.626-1.611)** |  |  |  |  |
| **Age, years** |  | **0.073** |  |  |  |
| ≤65 | **Reference** |  |  |  |  |
| ＞65 | **1.537 (0.961-2.460)** |  |  |  |  |
| **ECOG PS** |  | **0.631** |  |  |  |
| 2 | **Reference** |  |  |  |  |
| 0-1 | **0.898 (0.579-1.393)** |  |  |  |  |
| **T stage** |  | **0.760** |  |  |  |
| T4 | **Reference** |  |  |  |  |
| T2/T3 | **0.921 (0.542-1.565)** |  |  |  |  |
| **N stage** |  | **0.014** |  |  | **0.040** |
| N+ | **Reference** |  |  | **Referenc** |  |
| N0 | **0.498 (0.285-0.870)** |  |  | **0.583 (0.347-0.977)** |  |
| **Primary site** |  | **0.682** |  |  |  |
| Up 1/3 | **Reference** |  |  |  |  |
| Middle 1/3 | **1.204 (0.733-1.979)** | **0.463** |  |  |  |
| Lower 1/3 | **1.308 (0.621-2.754)** | **0.479** |  |  |  |
| **Tumor size, cm** |  | **0.947** |  |  |  |
| ≤5 | **Reference** |  |  |  |  |
| ＞5 | **1.015 (0.652-1.580)** |  |  |  |  |
| **Histology** |  | **0.715** |  |  |  |
| None/Low | **Reference** |  |  |  |  |
| High/Median | **0.920 (0.589-1.438)** |  |  |  |  |
| **SII^*^** |  | **0.223** |  |  |  |
| Low | **Reference** |  |  |  |  |
| High | **1.307 (0.850-2.009)** |  |  |  |  |
| **PNI^*^** |  | **0.942** |  |  |  |
| High | **Reference** |  |  |  |  |
| Low | **1.019 (0.605-1.717)** |  |  |  |  |
| **NLR^*^** |  | **0.660** |  |  |  |
| High | **Reference** |  |  |  |  |
| Low | **0.905 (0.580-1.412)** |  |  |  |  |
| **PLR^*^** |  | **0.264** |  |  |  |
| High | **Reference** |  |  |  |  |
| Low | **0.778 (0.501-1.209)** |  |  |  |  |
| **Recurrence** |  | **0.602** |  |  |  |
| Yes | **Reference** |  |  |  |  |
| No | **0.816 (0.380-1.751)** |  |  |  |  |
| **Radiomics score** |  | **0.237** |  |  |  |
| High | **Reference** |  |  |  |  |
| Low | **0.676 (0.353-1.294)** |  |  |  |  |
| **Pathomic score** |  | **0.159** |  |  |  |
| High | **Reference** |  |  |  |  |
| Low | **0.716 (0.450-1.140)** |  |  |  |  |
| **RSA model score** |  | **0.001** |  |  | **＜0.001** |
| Low | **Reference** |  |  | **Reference** |  |
| High | **4.272 (1.818-10.038)** |  |  | **2.381 (1.487-3.814)** |  |

**Note:** ECOG PS = Eastern Cooperative Oncology Group Performance Status; SII = Systemic Immune-inflammation Index; PNI = Prognostic Nutritional Index; NLR = Neutrophil to Lymphocyte Ratio; PLR = Platelet to Lymphpcyte Ratio. * Divided into high and low groups using the median as the critical value.

**Table S17. Univariate and multivariate Cox regression analysis of the factors affecting the 5-year overall survival rate of LAGC patients after surgery in the internal validation set -2 (2017-2019).**

| **Variable** | **Univariate analysis** | |  | **Multivariate analysis** | |
| --- | --- | --- | --- | --- | --- |
|  | **HR (95%CI)** | ***P* value** |  | **HR (95%CI)** | ***P* value** |
| **Gender** |  | **0.993** |  |  |  |
| Male | **Reference** |  |  |  |  |
| Female | **0.998 (0.636-1.566)** |  |  |  |  |
| **Age, years** |  | **0.988** |  |  |  |
| ≤65 | **Reference** |  |  |  |  |
| ＞65 | **0.997 (0.643-1.544)** |  |  |  |  |
| **ECOG PS** |  | **0.191** |  |  |  |
| 0-1 | **Reference** |  |  |  |  |
| 2 | **1.339 (0.865-2.072)** |  |  |  |  |
| **T stage** |  | **0.671** |  |  |  |
| T4 | **Reference** |  |  |  |  |
| T2/T3 | **0.885 (0.504-1.554)** |  |  |  |  |
| **N stage** |  | **0.664** |  |  |  |
| N+ | **Reference** |  |  |  |  |
| N0 | **0.888 (0.519-1.519)** |  |  |  |  |
| **Primary site** |  | **0.556** |  |  |  |
| Up 1/3 | **Reference** |  |  |  |  |
| Middle 1/3 | **1.308 (0.796-2.150)** | **0.290** |  |  |  |
| Lower 1/3 | **1.212 (0.597-2.459)** | **0.594** |  |  |  |
| **Tumor size, cm** |  | **0.377** |  |  |  |
| ＞5 | **Reference** |  |  |  |  |
| ≤5 | **0.817 (0.521-1.280)** |  |  |  |  |
| **Histology** |  | **0.399** |  |  |  |
| None/Low | **Reference** |  |  |  |  |
| High/Median | **0.828 (0.535-1.283)** |  |  |  |  |
| **SII^*^** |  | **0.010** |  |  | **0.062** |
| High | **Reference** |  |  | **Reference** |  |
| Low | **0.558 (0.358-0.869)** |  |  | **0.674 (0.446-1.020)** |  |
| **PNI^*^** |  | **0.448** |  |  |  |
| Low | **Reference** |  |  |  |  |
| High | **0.824 (0.500-1.358)** |  |  |  |  |
| **NLR^*^** |  | **0.201** |  |  |  |
| High | **Reference** |  |  |  |  |
| Low | **0.748 (0.479-1.167)** |  |  |  |  |
| **PLR^*^** |  | **0.396** |  |  |  |
| Low | **Reference** |  |  |  |  |
| High | **0.828 (0.535-1.281)** |  |  |  |  |
| **Recurrence** |  | **0.103** |  |  |  |
| No | **Reference** |  |  |  |  |
| Yes | **1.646 (0.905-2.997)** |  |  |  |  |
| **Radiomics score** |  | **0.095** |  |  |  |
| High | **Reference** |  |  |  |  |
| Low | **0.628 (0.364-1.084)** |  |  |  |  |
| **Pathomic score** |  | **0.141** |  |  |  |
| High | **Reference** |  |  |  |  |
| Low | **0.629 (0.340-1.165)** |  |  |  |  |
| **RSA model score** |  | **0.016** |  |  | **0.001** |
| Low | **Reference** |  |  | **Reference** |  |
| High | **2.790 (1.212-6.424)** |  |  | **2.138 (1.390-3.286)** |  |

**Note:** ECOG PS = Eastern Cooperative Oncology Group Performance Status; SII = Systemic Immune-inflammation Index; PNI = Prognostic Nutritional Index; NLR = Neutrophil to Lymphocyte Ratio; PLR = Platelet to Lymphpcyte Ratio. * Divided into high and low groups using the median as the critical value.

**Table S18. Baseline characteristics of patients in the internal validation set were stratified by adjuvant chemotherapy received before and after PSM.**

| **Variable** | **Before PSM** | | ***P* value** | **After PSM** | | ***P* value** |
| --- | --- | --- | --- | --- | --- | --- |
|  | **Chemotherapy**  **(N=308)** | **Non-Chemotherapy**  **(N=54)** |  | **Chemotherapy**  **(N=46)** | **Non-Chemotherapy**  **(N=46)** |  |
| **Gender** |  |  | **0.079** |  |  | **0.527** |
| Male | **193(62.7%)** | **27(50.0%)** |  | **28(60.9%)** | **25(54.3%)** |  |
| Female | **115(37.3%)** | **27(50.0%)** |  | **18(39.1%)** | **21(45.7%)** |  |
| **Age, years** |  |  | **0.534** |  |  | **0.830** |
| ≤65 | **157(51.0%)** | **30(55.6%)** |  | **29(63.0%)** | **28(60.9%)** |  |
| ＞65 | **151(49.0%)** | **24(44.4%)** |  | **17(37.0%)** | **18(39.1%)** |  |
| **ECOG PS** |  |  | **0.305** |  |  | **0.529** |
| 0-1 | **165(53.6%)** | **33(61.1%)** |  | **24(52.2%)** | **27(58.7%)** |  |
| 2 | **143(46.4%)** | **21(38.9%)** |  | **22(47.8%)** | **19(41.3%)** |  |
| **T stage** |  |  | **0.292** |  |  | **0.797** |
| T2/T3 | **90(29.2%)** | **12(22.2%)** |  | **9(19.6%)** | **10(21.7%)** |  |
| T4 | **218(70.8%)** | **42(77.8%)** |  | **37(80.4%)** | **36(78.3%)** |  |
| **N stage** |  |  | **0.838** |  |  | **0.562** |
| N0 | **84(27.3%)** | **14(25.9%)** |  | **6(13.0%)** | **8(17.4%)** |  |
| N+ | **224(72.7%)** | **40(74.1%)** |  | **40(87.0%)** | **38(82.6%)** |  |
| **Primary site** |  |  | **0.110** |  |  | **0.289** |
| Up 1/3 | **106(34.4%)** | **13(24.1%)** |  | **18(39.1%)** | **11(23.9%)** |  |
| Middle 1/3 | **31(10.1%)** | **10(18.5%)** |  | **6(13.0%)** | **8(17.4%)** |  |
| Lower 1/3 | **171(55.5%)** | **31(57.4%)** |  | **22(47.8%)** | **27(58.7%)** |  |
| **Tumor size, cm** |  |  | **0.107** |  |  | **1.000** |
| ≤5 | **169(54.9%)** | **36(66.7%)** |  | **27(58.7%)** | **27(58.7%)** |  |
| ＞5 | **139(45.1%)** | **18(33.3%)** |  | **19(41.3%)** | **19(41.3%)** |  |
| **Histology** |  |  | **0.939** |  |  | **0.804** |
| None/Low | **241(78.2%)** | **42(77.8%)** |  | **36(78.3%)** | **35(76.1%)** |  |
| High/Median | **67(21.8%)** | **12(22.2%)** |  | **10(21.7%)** | **11(23.9%)** |  |
| **SII^*^** |  |  | **0.870** |  |  | **0.400** |
| Low | **156(50.6%)** | **28(51.9%)** |  | **28(60.9%)** | **24(52.2%)** |  |
| High | **152(49.4%)** | **26(48.1%)** |  | **18(39.1%)** | **22(47.8%)** |  |
| **PNI^*^** |  |  | **0.132** |  |  | **0.815** |
| Low | **63(20.5%)** | **16(29.6%)** |  | **12(26.1%)** | **13(28.3%)** |  |
| High | **245(79.5%)** | **38(70.4%)** |  | **34(73.9%)** | **33(71.7%)** |  |
| **NLR^*^** |  |  | **0.205** |  |  | **0.666** |
| Low | **177(57.5%)** | **36(66.7%)** |  | **28(60.9%)** | **30(65.2%)** |  |
| High | **131(42.5%)** | **18(33.3%)** |  | **18(39.1%)** | **16(34.8%)** |  |
| **PLR^*^** |  |  | **0.919** |  |  | **0.834** |
| Low | **146(47.4%)** | **26(48.1%)** |  | **21(45.7%)** | **22(47.8%)** |  |
| High | **162(52.6%)** | **28(51.9%)** |  | **25(54.3%)** | **24(52.2%)** |  |

**Note:** ECOG PS = Eastern Cooperative Oncology Group Performance Status; SII = Systemic Immune-inflammation Index; PNI = Prognostic Nutritional Index; NLR = Neutrophil to Lymphocyte Ratio; PLR = Platelet to Lymphpcyte Ratio. * Divided into high and low groups using the median as the critical value

**Table S19. Univariate and multivariate analyses of overall survival were performed for patients with an RSA model score of 0.19 or greater in the matched internal validation set.**

| **Variable** | **Univariate analysis** | |  | **Multivariate analysis** | |
| --- | --- | --- | --- | --- | --- |
|  | **HR (95%CI)** | ***P* value** |  | **HR (95%CI)** | ***P* value** |
| **Gender** |  | **0.070** |  |  |  |
| Male | **Reference** |  |  |  |  |
| Female | **1.997 (0.946-4.216)** |  |  |  |  |
| **Age, years** |  | **0.119** |  |  |  |
| ≤65 | **Reference** |  |  |  |  |
| ＞65 | **1.742 (0.866-3.504)** |  |  |  |  |
| **ECOG PS** |  | **0.248** |  |  |  |
| 0-1 | **Reference** |  |  |  |  |
| 2 | **1.475 (0.763-2.852)** |  |  |  |  |
| **T stage** |  | **0.619** |  |  |  |
| T2/T3 | **Reference** |  |  |  |  |
| T4 | **1.196 (0.591-2.424)** |  |  |  |  |
| **N stage** |  | **0.022** |  |  | **0.090** |
| N+ | **Reference** |  |  | **Reference** |  |
| N0 | **0.412 (0.193-0.880)** |  |  | **0.558 (0.284-1.096)** |  |
| **Primary site** |  | **0.555** |  |  |  |
| Up 1/3 | **Reference** |  |  |  |  |
| Middle 1/3 | **1.115 (0.523-2.377)** | **0.779** |  |  |  |
| Lower 1/3 | **1.744 (0.634-4.795)** | **0.281** |  |  |  |
| **Tumor size, cm** |  | **0.101** |  |  |  |
| ≤5 | **Reference** |  |  |  |  |
| ＞5 | **1.751 (0.896-3.422)** |  |  |  |  |
| **Histology** |  | **0.881** |  |  |  |
| High/Median | **Reference** |  |  |  |  |
| None/Low | **0.950 (0.486-1.859)** |  |  |  |  |
| **Chemotherapy** |  | **0.005** |  |  | **0.007** |
| Yes | **Reference** |  |  | **Reference** |  |
| No | **2.842 (1.377-5.868)** |  |  | **2.390 (1.271-4.492)** |  |
| **SII^*^** |  | **0.712** |  |  |  |
| High | **Reference** |  |  |  |  |
| Low | **0.882 (0.453-1.719)** |  |  |  |  |
| **PNI^*^** |  | **0.185** |  |  |  |
| Low | **Reference** |  |  |  |  |
| High | **0.890 (0.434-1.824)** |  |  |  |  |
| **NLR^*^** |  | **0.185** |  |  |  |
| High | **Reference** |  |  |  |  |
| Low | **0.619 (0.305-1.258)** |  |  |  |  |
| **PLR^*^** |  | **0.118** |  |  |  |
| High | **Reference** |  |  |  |  |
| Low | **0.583 (0.296-1.147)** |  |  |  |  |

**Note:** ECOG PS = Eastern Cooperative Oncology Group Performance Status; SII = Systemic Immune-inflammation Index; PNI = Prognostic Nutritional Index; NLR = Neutrophil to Lymphocyte Ratio; PLR = Platelet to Lymphpcyte Ratio. * Divided into high and low groups using the median as the critical value

**Table S20. Comparison of clinical characteristics of LAGC patients in the external validation set.**

| **Variable** | **Total**  **(N=448)** | **External validation-1 (Northern-N=257)** | **External validation-2 (Southern-N=191)** | ***P* value** |
| --- | --- | --- | --- | --- |
| **Gender** |  |  |  | **0.302** |
| Male | **305(68.1%)** | **180(70.0%)** | **125(65.4%)** |  |
| Female | **143(31.9%)** | **77(30.0%)** | **66(34.6%)** |  |
| **Age, years** |  |  |  | **0.619** |
| ≤65 | **198(44.2%)** | **111(43.2%)** | **87(45.5%)** |  |
| ＞65 | **250(55.8%)** | **146(56.8%)** | **104(54.5%)** |  |
| **ECOG PS** |  |  |  | **0.857** |
| 0-1 | **218(48.7%)** | **126(49.0%)** | **92(48.2%)** |  |
| 2 | **230(51.3%)** | **131(51.0%)** | **99(51.8%)** |  |
| **T stage** |  |  |  | **0.307** |
| T2/T3 | **102(22.8%)** | **63(24.5%)** | **39(20.4%)** |  |
| T4 | **346(77.2%)** | **194(75.5%)** | **152(79.6%)** |  |
| **N stage** |  |  |  | **0.522** |
| N0 | **122(27.2%)** | **67(26.1%)** | **55(28.8%)** |  |
| N+ | **326(72.8%)** | **190(73.9%)** | **136(71.2%)** |  |
| **Primary site** |  |  |  | **0.050** |
| Up 1/3 | **150(33.5%)** | **89(34.6%)** | **61(31.9%)** |  |
| Middle 1/3 | **65(14.5%)** | **45(17.5%)** | **20(10.5%)** |  |
| Lower 1/3 | **233(52.0%)** | **123(47.9%)** | **110(57.6%)** |  |
| **Tumor size, cm** |  |  |  | **0.651** |
| ≤5 | **229(51.1%)** | **129(50.2%)** | **100(52.4%)** |  |
| ＞5 | **219(48.9%)** | **128(49.8%)** | **91(47.6%)** |  |
| **Histology** |  |  |  | **0.051** |
| None/Low | **361(80.6%)** | **199(77.4%)** | **162(84.8%)** |  |
| High/Median | **87(19.4%)** | **58(22.6%)** | **29(15.2%)** |  |
| **SII^*^** |  |  |  | **0.742** |
| Low | **262(58.5%)** | **152(59.1%)** | **110(57.6%)** |  |
| High | **186(41.5%)** | **105(40.9%)** | **81(42.4%)** |  |
| **PNI^*^** |  |  |  | **0.052** |
| Low | **92(20.5%)** | **61(23.7%)** | **31(16.2%)** |  |
| High | **356(79.5%)** | **196(76.3%)** | **160(83.8%)** |  |
| **NLR^*^** |  |  |  | **0.994** |
| Low | **272(60.7%)** | **156(60.7%)** | **116(60.7%)** |  |
| High | **176(39.3%)** | **101(39.3%)** | **75(39.3%)** |  |
| **PLR^*^** |  |  |  | **0.508** |
| Low | **189(42.2%)** | **105(40.9%)** | **84(44.0%)** |  |
| High | **259(57.8%)** | **152(59.1%)** | **107(56.0%)** |  |

**Note:** ECOG PS = Eastern Cooperative Oncology Group Performance Status; SII = Systemic Immune-inflammation Index; PNI = Prognostic Nutritional Index; NLR = Neutrophil to Lymphocyte Ratio; PLR = Platelet to Lymphpcyte Ratio. * Divided into high and low groups using the median as the critical value.

**Table S21. Comparison of performance indicators of different models in predicting postoperative recurrence in patients with LAGC in external validation set.**

| **Variable** | **AUC** | **Accuracy** | **Sensitivity** | **Specificity** | **PPV** | **NPV** | **PLR (x10)** | **NLR** | **F1 score** |
| --- | --- | --- | --- | --- | --- | --- | --- | --- | --- |
| **External validation set (Northern)** |  |  |  |  |  |  |  |  |  |
| Clinical features | **0.774** | **0.693** | **0.728** | **0.645** | **0.733** | **0.640** | **0.422** | **0.730** | **0.205** |
| Radiomics | **0.790** | **0.696** | **0.735** | **0.645** | **0.735** | **0.645** | **0.411** | **0.735** | **0.207** |
| Pathomics | **0.739** | **0.673** | **0.714** | **0.618** | **0.714** | **0.618** | **0.462** | **0.714** | **0.187** |
| RSA model | **0.884** | **0.813** | **0.864** | **0.745** | **0.819** | **0.804** | **0.183** | **0.841** | **0.339** |
| **External validation set (Southern)** |  |  |  |  |  |  |  |  |  |
| Clinical features | **0.767** | **0.696** | **0.738** | **0.648** | **0.710** | **0.679** | **0.405** | **0.724** | **0.209** |
| Radiomics | **0.789** | **0.754** | **0.748** | **0.761** | **0.786** | **0.720** | **0.332** | **0.766** | **0.313** |
| Pathomics | **0.736** | **0.702** | **0.738** | **0.659** | **0.717** | **0.682** | **0.398** | **0.727** | **0.216** |
| RSA model | **0.896** | **0.838** | **0.835** | **0.841** | **0.860** | **0.813** | **0.196** | **0.847** | **0.525** |

Note: AUC-area under the curve; PPV-Positive Predictive Value; NVP-Negative Predictive Value; PLR-Positive Likelihood Ratio; NLR-Negative Likelihood Ratio.

**Table S22. Univariate and multivariate Cox regression analysis of the factors affecting the 5-year overall survival rate of LAGC patients after surgery in the external validation-1 (Northern).**

| **Variable** | **Univariate analysis** | |  | **Multivariate analysis** | |
| --- | --- | --- | --- | --- | --- |
|  | **HR (95%CI)** | ***P* value** |  | **HR (95%CI)** | ***P* value** |
| **Gender** |  | **0.711** |  |  |  |
| Male | **Reference** |  |  |  |  |
| Female | **1.076 (0.731-1.583)** |  |  |  |  |
| **Age, years** |  | **0.787** |  |  |  |
| ≤65 | **Reference** |  |  |  |  |
| ＞65 | **0.951 (0.661-1.369)** |  |  |  |  |
| **ECOG PS** |  | **0.686** |  |  |  |
| 2 | **Reference** |  |  |  |  |
| 0-1 | **0.930 (0.652-1.325)** |  |  |  |  |
| **T stage** |  | **0.331** |  |  |  |
| T2/T3 | **Reference** |  |  |  |  |
| T4 | **1.245 (0.800-1.937)** |  |  |  |  |
| **N stage** |  | **0.813** |  |  |  |
| N0 | **Reference** |  |  |  |  |
| N+ | **1.061 (0.650-1.732)** |  |  |  |  |
| **Primary site** |  | **0.082** |  |  |  |
| Up 1/3 | **Reference** |  |  |  |  |
| Middle 1/3 | **1.487 (0.999-2.214)** | **0.051** |  |  |  |
| Lower 1/3 | **0.921 (0.550-1.540)** | **0.753** |  |  |  |
| **Tumor size, cm** |  | **0.207** |  |  |  |
| ≤5 | **Reference** |  |  |  |  |
| ＞5 | **1.262 (0.879-1.813)** |  |  |  |  |
| **Histology** |  | **0.994** |  |  |  |
| High/Median | **Reference** |  |  |  |  |
| None/Low | **1.001 (0.693-1.448)** |  |  |  |  |
| **SII^*^** |  | **0.495** |  |  |  |
| High | **Reference** |  |  |  |  |
| Low | **0.839 (0.508-1.388)** |  |  |  |  |
| **PNI^*^** |  | **0.258** |  |  |  |
| Low | **Reference** |  |  |  |  |
| High | **0.794 (0.533-1.184)** |  |  |  |  |
| **NLR^*^** |  | **0.251** |  |  |  |
| Low | **Reference** |  |  |  |  |
| High | **1.355 (0.807-2.273)** |  |  |  |  |
| **PLR^*^** |  | **0.370** |  |  |  |
| Low | **Reference** |  |  |  |  |
| High | **0.847 (0.589-1.218)** |  |  |  |  |
| **Recurrence** |  | **0.136** |  |  |  |
| Yes | **Reference** |  |  |  |  |
| No | **0.695 (0.430-1.122)** |  |  |  |  |
| **Radiomics score** |  | **0.347** |  |  |  |
| High | **Reference** |  |  |  |  |
| Low | **0.765 (0.439-1.336)** |  |  |  |  |
| **Pathomic score** |  | **0.891** |  |  |  |
| High | **Reference** |  |  |  |  |
| Low | **0.955 (0.493-1.848)** |  |  |  |  |
| **RSA model score** |  | **0.001** |  |  | **＜0.001** |
| Low | **Reference** |  |  | **Reference** |  |
| High | **3.814 (1.746-8.329)** |  |  | **2.365 (1.596-3.505)** |  |

**Note:** ECOG PS = Eastern Cooperative Oncology Group Performance Status; SII = Systemic Immune-inflammation Index; PNI = Prognostic Nutritional Index; NLR = Neutrophil to Lymphocyte Ratio; PLR = Platelet to Lymphpcyte Ratio. * Divided into high and low groups using the median as the critical value.

**Table S23. Univariate and multivariate Cox regression analysis of the factors affecting the 5-year overall survival rate of LAGC patients after surgery in the external validation-2 (Southern).**

| **Variable** | **Univariate analysis** | |  | **Multivariate analysis** | |
| --- | --- | --- | --- | --- | --- |
|  | **HR (95%CI)** | ***P* value** |  | **HR (95%CI)** | ***P* value** |
| **Gender** |  | **0.356** |  |  |  |
| Male | **Reference** |  |  |  |  |
| Female | **1.237 (0.787-1.945)** |  |  |  |  |
| **Age, years** |  | **0.085** |  |  |  |
| ≤65 | **Reference** |  |  |  |  |
| ＞65 | **1.460 (0.949-2.246)** |  |  |  |  |
| **ECOG PS** |  | **0.034** |  |  | **0.079** |
| 0-1 | **Reference** |  |  | **Reference** |  |
| 2 | **1.593 (1.035-2.452)** |  |  | **1.439 (0.959-2.161)** |  |
| **T stage** |  | **0.205** |  |  |  |
| T2/T3 | **Reference** |  |  |  |  |
| T4 | **1.436 (0.820-2.515)** |  |  |  |  |
| **N stage** |  | **0.381** |  |  |  |
| N0 | **Reference** |  |  |  |  |
| N+ | **1.313 (0.714-2.413)** |  |  |  |  |
| **Primary site** |  | **0.200** |  |  |  |
| Up 1/3 | **Reference** |  |  |  |  |
| Middle 1/3 | **0.652 (0.402-1.056)** | **0.082** |  |  |  |
| Lower 1/3 | **0.761 (0.364-1.593)** | **0.469** |  |  |  |
| **Tumor size, cm** |  | **0.015** |  |  | **0.034** |
| ≤5 | **Reference** |  |  | **Reference** |  |
| ＞5 | **1.755 (1.113-2.766)** |  |  | **1.560 (1.035-2.350)** |  |
| **Histology** |  | **0.191** |  |  |  |
| High/Median | **Reference** |  |  |  |  |
| None/Low | **1.380 (0.852-2.237)** |  |  |  |  |
| **SII^*^** |  | **0.208** |  |  |  |
| High | **Reference** |  |  |  |  |
| Low | **0.744 (0.469-1.180)** |  |  |  |  |
| **PNI^*^** |  | **0.751** |  |  |  |
| High | **Reference** |  |  |  |  |
| Low | **1.099 (0.614-1.965)** |  |  |  |  |
| **NLR^*^** |  | **0.471** |  |  |  |
| Low | **Reference** |  |  |  |  |
| High | **1.195 (0.737-1.938)** |  |  |  |  |
| **PLR^*^** |  | **0.038** |  |  | **0.020** |
| High | **Reference** |  |  | **Reference** |  |
| Low | **0.627 (0.403-0.975)** |  |  | **0.618 (0.412-0.927)** |  |
| **Recurrence** |  | **0.638** |  |  |  |
| Yes | **Reference** |  |  |  |  |
| No | **0.863 (0.468-1.594)** |  |  |  |  |
| **Radiomics score** |  | **0.171** |  |  |  |
| High | **Reference** |  |  |  |  |
| Low | **0.706 (0.428-1.162)** |  |  |  |  |
| **Pathomic score** |  | **0.368** |  |  |  |
| High | **Reference** |  |  |  |  |
| Low | **0.773 (0.441-1.354)** |  |  |  |  |
| **RSA model score** |  | **0.011** |  |  | **＜0.001** |
| Low | **Reference** |  |  | **Reference** |  |
| High | **2.914 (1.274-6.667)** |  |  | **2.296 (1.505-3.503)** |  |

**Note:** ECOG PS = Eastern Cooperative Oncology Group Performance Status; SII = Systemic Immune-inflammation Index; PNI = Prognostic Nutritional Index; NLR = Neutrophil to Lymphocyte Ratio; PLR = Platelet to Lymphpcyte Ratio. * Divided into high and low groups using the median as the critical value.

**Table S24. Baseline characteristics of patients in the external validation set were stratified by adjuvant chemotherapy received before and after PSM.**

| **Variable** | **Before PSM** | | ***P* value** | **After PSM** | | ***P* value** |
| --- | --- | --- | --- | --- | --- | --- |
|  | **Chemotherapy**  **(N=371)** | **Non-Chemotherapy**  **(N=77)** |  | **Chemotherapy**  **(N=63)** | **Non-Chemotherapy**  **(N=63)** |  |
| **Gender** |  |  | **0.145** |  |  | **0.851** |
| Male | **258(69.5%)** | **47(61.0%)** |  | **42(66.7%)** | **41(65.1%)** |  |
| Female | **113(30.5%)** | **30(39.0%)** |  | **21(33.3%)** | **22(34.9%)** |  |
| **Age, years** |  |  | **0.807** |  |  | **0.211** |
| ≤65 | **163(43.9%)** | **35(45.5%)** |  | **26(41.3%)** | **33(52.4%)** |  |
| ＞65 | **208(56.1%)** | **42(54.5%)** |  | **37(58.7%)** | **30(47.6%)** |  |
| **ECOG PS** |  |  | **0.004** |  |  | **0.367** |
| 0-1 | **169(45.6%)** | **49(63.6%)** |  | **24(38.1%)** | **29(46.0%)** |  |
| 2 | **202(54.4%)** | **28(36.4%)** |  | **39(61.9%)** | **34(54.0%)** |  |
| **T stage** |  |  | **0.228** |  |  | **0.264** |
| T2/T3 | **83(22.4%)** | **22(28.6%)** |  | **10(15.9%)** | **15(23.8%)** |  |
| T4 | **288(77.6%)** | **55(71.4%)** |  | **53(84.1%)** | **48(76.2%)** |  |
| **N stage** |  |  | **0.048** |  |  | **0.328** |
| N0 | **94(25.3%)** | **28(36.4%)** |  | **21(33.3%)** | **16(25.4%)** |  |
| N+ | **277(74.7%)** | **49(63.6%)** |  | **42(66.7%)** | **47(74.6%)** |  |
| **Primary site** |  |  | **0.564** |  |  | **0.659** |
| Up 1/3 | **124(33.4%)** | **26(33.8%)** |  | **23(36.5%)** | **19(30.2%)** |  |
| Middle 1/3 | **51(13.7%)** | **14(18.2%)** |  | **4(6.3%)** | **6(9.5%)** |  |
| Lower 1/3 | **196(52.8%)** | **37(48.1%)** |  | **36(57.1%)** | **38(60.3%)** |  |
| **Tumor size, cm** |  |  | **0.362** |  |  | **0.722** |
| ≤5 | **186(50.1%)** | **43(55.8%)** |  | **31(49.2%)** | **33(52.4%)** |  |
| ＞5 | **185(49.9%)** | **34(44.2%)** |  | **32(50.8%)** | **30(47.6%)** |  |
| **Histology** |  |  | **0.000** |  |  | **0.573** |
| None/Low | **314(84.6%)** | **47(61.0%)** |  | **40(63.5%)** | **43(68.3%)** |  |
| High/Median | **57(15.4%)** | **30(39.0%)** |  | **23(36.5%)** | **20(31.7%)** |  |
| **SII^*^** |  |  | **0.125** |  |  | **0.593** |
| Low | **223(60.1%)** | **39(50.6%)** |  | **30(47.6%)** | **33(52.4%)** |  |
| High | **148(39.9%)** | **38(49.4%)** |  | **33(52.4%)** | **30(47.6%)** |  |
| **PNI^*^** |  |  | **0.498** |  |  | **0.086** |
| Low | **74(19.9%)** | **18(23.4%)** |  | **18(28.6%)** | **10(15.9%)** |  |
| High | **297(80.1%)** | **59(76.6%)** |  | **45(71.4%)** | **53(84.1%)** |  |
| **NLR^*^** |  |  | **0.564** |  |  | **0.577** |
| Low | **223(60.1%)** | **49(63.6%)** |  | **42(66.7%)** | **39(61.9%)** |  |
| High | **148(39.9%)** | **28(36.4%)** |  | **21(33.3%)** | **24(38.1%)** |  |
| **PLR^*^** |  |  | **0.902** |  |  | **0.183** |
| Low | **157(42.3%)** | **32(41.6%)** |  | **17(27.0%)** | **24(38.1%)** |  |
| High | **214(57.7%)** | **45(58.4%)** |  | **46(73.0%)** | **39(61.9%)** |  |

**Note:** ECOG PS = Eastern Cooperative Oncology Group Performance Status; SII = Systemic Immune-inflammation Index; PNI = Prognostic Nutritional Index; NLR = Neutrophil to Lymphocyte Ratio; PLR = Platelet to Lymphpcyte Ratio. * Divided into high and low groups using the median as the critical value

**Table S25. Univariate and multivariate analyses of overall survival were performed for patients with an RSA model score of 0.19 or greater in the matched external validation set.**

| **Variable** | **Univariate analysis** | |  | **Multivariate analysis** | |
| --- | --- | --- | --- | --- | --- |
|  | **HR (95%CI)** | ***P* value** |  | **HR (95%CI)** | ***P* value** |
| **Gender** |  | **0.070** |  |  |  |
| Male | **Reference** |  |  |  |  |
| Female | **1.997 (0.946-4.216)** |  |  |  |  |
| **Age, years** |  | **0.119** |  |  |  |
| ≤65 | **Reference** |  |  |  |  |
| ＞65 | **1.742 (0.866-3.504)** |  |  |  |  |
| **ECOG PS** |  | **0.248** |  |  |  |
| 0-1 | **Reference** |  |  |  |  |
| 2 | **1.475 (0.763-2.852)** |  |  |  |  |
| **T stage** |  | **0.619** |  |  |  |
| T2/T3 | **Reference** |  |  |  |  |
| T4 | **1.196 (0.591-2.424)** |  |  |  |  |
| **N stage** |  | **0.022** |  |  | **0.090** |
| N+ | **Reference** |  |  | **Reference** |  |
| N0 | **0.412 (0.193-0.880)** |  |  | **0.558 (0.284-1.096)** |  |
| **Primary site** |  | **0.555** |  |  |  |
| Up 1/3 | **Reference** |  |  |  |  |
| Middle 1/3 | **1.115 (0.523-2.377)** | **0.779** |  |  |  |
| Lower 1/3 | **1.744 (0.634-4.795)** | **0.281** |  |  |  |
| **Tumor size, cm** |  | **0.101** |  |  |  |
| ≤5 | **Reference** |  |  |  |  |
| ＞5 | **1.751 (0.896-3.422)** |  |  |  |  |
| **Histology** |  | **0.881** |  |  |  |
| High/Median | **Reference** |  |  |  |  |
| None/Low | **0.950 (0.486-1.859)** |  |  |  |  |
| **Chemotherapy** |  | **0.005** |  |  | **0.007** |
| Yes | **Reference** |  |  | **Reference** |  |
| No | **2.842 (1.377-5.868)** |  |  | **2.390 (1.271-4.492)** |  |
| **SII^*^** |  | **0.712** |  |  |  |
| High | **Reference** |  |  |  |  |
| Low | **0.882 (0.453-1.719)** |  |  |  |  |
| **PNI^*^** |  | **0.185** |  |  |  |
| Low | **Reference** |  |  |  |  |
| High | **0.890 (0.434-1.824)** |  |  |  |  |
| **NLR^*^** |  | **0.185** |  |  |  |
| High | **Reference** |  |  |  |  |
| Low | **0.619 (0.305-1.258)** |  |  |  |  |
| **PLR^*^** |  | **0.118** |  |  |  |
| High | **Reference** |  |  |  |  |
| Low | **0.583 (0.296-1.147)** |  |  |  |  |

**Note:** ECOG PS = Eastern Cooperative Oncology Group Performance Status; SII = Systemic Immune-inflammation Index; PNI = Prognostic Nutritional Index; NLR = Neutrophil to Lymphocyte Ratio; PLR = Platelet to Lymphpcyte Ratio. * Divided into high and low groups using the median as the critical value

**Table S26. Comparison of clinical characteristics of LAGC patients in the prospective validation cohort (NCT 02555358).**

| **Variable** | **Total**  **(N=257)** | **Surgery Cohort (N=93)** | **XELOX Cohort**  **(N=86)** | **DOX Cohort**  **(N=78)** | ***P* value** |
| --- | --- | --- | --- | --- | --- |
| **Gender** |  |  |  |  | **0.355** |
| Male | **173(67.3%)** | **64(68.8%)** | **53(61.6%)** | **56(71.8%)** |  |
| Female | **84(32.7%)** | **29(31.2%)** | **33(38.4%)** | **22(28.2%)** |  |
| **Age, years** |  |  |  |  | **0.660** |
| ≤65 | **123(47.9%)** | **41(44.1%)** | **43(50.0%)** | **39(50.0%)** |  |
| ＞65 | **134(52.1%)** | **52(55.9%)** | **43(50.0%)** | **39(50.0%)** |  |
| **ECOG PS** |  |  |  |  | **0.499** |
| 0-1 | **123(47.9%)** | **47(50.5%)** | **43(50.0%)** | **33(42.3%)** |  |
| 2 | **134(52.1%)** | **46(49.5%)** | **43(50.0%)** | **45(57.7%)** |  |
| **T stage** |  |  |  |  | **0.373** |
| T2/T3 | **68(26.5%)** | **29(31.2%)** | **22(25.6%)** | **17(21.8%)** |  |
| T4 | **189(73.5%)** | **64(68.8%)** | **64(74.4%)** | **61(78.2%)** |  |
| **N stage** |  |  |  |  | **0.085** |
| N0 | **78(30.4%)** | **26(28.0%)** | **21(24.4%)** | **31(39.7%)** |  |
| N+ | **179(69.6%)** | **67(72.0%)** | **65(75.6%)** | **47(60.3%)** |  |
| **Primary site** |  |  |  |  | **0.751** |
| Up 1/3 | **91(35.4%)** | **32(34.4%)** | **29(33.7%)** | **30(38.5%)** |  |
| Middle 1/3 | **38(14.8%)** | **16(17.2%)** | **10(11.6%)** | **12(15.4%)** |  |
| Lower 1/3 | **128(49.8%)** | **45(48.4%)** | **47(54.7%)** | **36(46.2%)** |  |
| **Tumor size, cm** |  |  |  |  | **0.926** |
| ≤5 | **144(56.0%)** | **52(55.9%)** | **47(54.7%)** | **45(57.7%)** |  |
| ＞5 | **113(44.0%)** | **41(44.1%)** | **39(45.3%)** | **33(42.3%)** |  |
| **Histology** |  |  |  |  | **0.199** |
| None/Low | **215(83.7%)** | **73(78.5%)** | **73(84.9%)** | **69(88.5%)** |  |
| High/Median | **42(16.3%)** | **20(21.5%)** | **13(15.1%)** | **9(11.5%)** |  |
| **SII^*^** |  |  |  |  | **0.366** |
| Low | **154(59.9%)** | **61(65.6%)** | **48(55.8%)** | **45(57.7%)** |  |
| High | **103(40.1%)** | **32(34.4%)** | **38(44.2%)** | **33(42.3%)** |  |
| **PNI^*^** |  |  |  |  | **0.219** |
| Low | **45(17.5%)** | **12(12.9%)** | **15(17.4%)** | **18(23.1%)** |  |
| High | **212(82.5%)** | **81(87.1%)** | **71(82.6%)** | **60(76.9%)** |  |
| **NLR^*^** |  |  |  |  | **0.841** |
| Low | **152(59.1%)** | **57(61.3%)** | **49(57.0%)** | **46(59.0%)** |  |
| High | **105(40.9%)** | **36(38.7%)** | **37(43.0%)** | **32(41.0%)** |  |
| **PLR^*^** |  |  |  |  | **0.244** |
| Low | **119(46.3%)** | **47(50.5%)** | **42(48.8%)** | **30(38.5%)** |  |
| High | **138(53.7%)** | **46(49.5%)** | **44(51.2%)** | **48(61.5%)** |  |

**Note:** ECOG PS = Eastern Cooperative Oncology Group Performance Status; SII = Systemic Immune-inflammation Index; PNI = Prognostic Nutritional Index; NLR = Neutrophil to Lymphocyte Ratio; PLR = Platelet to Lymphpcyte Ratio. * Divided into high and low groups using the median as the critical value.

**Table S27. Comparison of performance indicators of different models in predicting postoperative recurrence in patients with LAGC in prospective validation set.**

| **Variable** | **AUC** | **Accuracy** | **Sensitivity** | **Specificity** | **PPV** | **NPV** | **PLR (x10)** | **NLR** | **F1 score** |
| --- | --- | --- | --- | --- | --- | --- | --- | --- | --- |
| **Surgical cohort** |  |  |  |  |  |  |  |  |  |
| Clinical features | **0.774** | **0.742** | **0.780** | **0.698** | **0.750** | **0.732** | **0.315** | **0.765** | **0.258** |
| Radiomics | **0.792** | **0.720** | **0.700** | **0.744** | **0.761** | **0.681** | **0.403** | **0.729** | **0.274** |
| Pathomics | **0.708** | **0.677** | **0.820** | **0.512** | **0.661** | **0.710** | **0.352** | **0.732** | **0.168** |
| RSA model | **0.889** | **0.817** | **0.840** | **0.791** | **0.824** | **0.810** | **0.202** | **0.832** | **0.401** |
| **Neoadjuvant chemotherapy cohort** |  |  |  |  |  |  |  |  |  |
| Clinical features | **0.686** | **0.683** | **0.759** | **0.612** | **0.645** | **0.732** | **0.393** | **0.698** | **0.196** |
| Radiomics | **0.694** | **0.677** | **0.732** | **0.635** | **0.648** | **0.711** | **0.438** | **0.683** | **0.198** |
| Pathomics | **0.627** | **0.604** | **0.658** | **0.553** | **0.578** | **0.635** | **0.618** | **0.615** | **0.147** |
| RSA model | **0.736** | **0.707** | **0.861** | **0.565** | **0.648** | **0.814** | **0.247** | **0.739** | **0.198** |

Note: AUC-area under the curve; PPV-Positive Predictive Value; NVP-Negative Predictive Value; PLR-Positive Likelihood Ratio; NLR-Negative Likelihood Ratio

**Table S28. Comparison of baseline characteristics before and after PSM in LAGC patients with different TNM stages.**

| **Variable** | **TNM stage I** | | | | | |
| --- | --- | --- | --- | --- | --- | --- |
|  | **Before PSM** | | ***P* value** | **After PSM** | | ***P* value** |
|  | **RSA high**  **(N=31)** | **RSA low (N=95)** |  | **RSA high**  **(N=30)** | **RSA low (N=30)** |  |
| **Gender** |  |  | **0.172** |  |  | **0.222** |
| Male | **26(83.9%)** | **68(71.6%)** |  | **25(83.3%)** | **21(70.0%)** |  |
| Female | **5(16.1%)** | **27(28.4%)** |  | **5(16.7%)** | **9(30.0%)** |  |
| **Age, years** |  |  | **0.997** |  |  | **0796** |
| ≤65 | **16(51.6%)** | **49(51.6%)** |  | **15(50.0%)** | **14(46.7%)** |  |
| ＞65 | **15(48.4%)** | **46(48.4%)** |  | **15(50.0%)** | **16(53.3%)** |  |
| **ECOG PS** |  |  | **0.831** |  |  | **0.795** |
| 0-1 | **14(45.2%)** | **45(47.4%)** |  | **13(43.3%)** | **14(46.7%)** |  |
| 2 | **17(54.8%)** | **50(52.6%)** |  | **17(56.7%)** | **16(53.3%)** |  |
| **Primary site** |  |  | **0.836** |  |  | **0.647** |
| Up 1/3 | **11(35.5%)** | **31(32.6%)** |  | **16(53.3%)** | **20(66.7%)** |  |
| Middle 1/3 | **3(9.7%)** | **13(13.7%)** |  | **4(13.3%)** | **3(10.0%)** |  |
| Lower 1/3 | **17(54.8%)** | **51(53.7%)** |  | **10(33.3%)** | **7(23.3%)** |  |
| **Tumor size, cm** |  |  | **0.354** |  |  | **1.000** |
| ≤5 | **16(51.6%)** | **58(61.1%)** |  | **16(53.3%)** | **16(53.3%)** |  |
| ＞5 | **15(48.4%)** | **37(38.9%)** |  | **14(46.7%)** | **14(46.7%)** |  |
| **Histology** |  |  | **0.163** |  |  | **0.584** |
| None/Low | **22(71.0%)** | **54(56.8%)** |  | **21(70.0%)** | **19(63.3%)** |  |
| High/Median | **9(29.0%)** | **41(43.2%)** |  | **9(30.0%)** | **11(36.7%)** |  |
| **SII^*^** |  |  | **0.631** |  |  | **0.774** |
| Low | **22(71.0%)** | **63(66.3%)** |  | **22(73.3%)** | **21(70.0%)** |  |
| High | **9(29.0%)** | **32(33.7%)** |  | **8(26.7%)** | **9(30.0%)** |  |
| **PNI^*^** |  |  | **0.355** |  |  | **0.795** |
| Low | **16(51.6%)** | **40(42.1%)** |  | **16(53.3%)** | **17(56.7%)** |  |
| High | **15(48.4%)** | **55(57.9%)** |  | **14(46.7%)** | **13(43.3%)** |  |
| **NLR^*^** |  |  | **0.413** |  |  | **0.766** |
| Low | **23(74.2%)** | **63(66.3%)** |  | **23(76.7%)** | **22(73.3%)** |  |
| High | **8(25.8%)** | **32(33.7%)** |  | **7(23.3%)** | **8(26.7%)** |  |
| **PLR^*^** |  |  | **0.306** |  |  | **0.176** |
| Low | **8(25.8%)** | **34(35.8%)** |  | **8(26.7%)** | **13(43.3%)** |  |
| High | **23(74.2%)** | **61(64.2%)** |  | **22(73.3%)** | **17(56.7%)** |  |
| **Variable** | **TNM stage II** | | | | | |
|  | **Before PSM** | | ***P* value** | **After PSM** | | ***P* value** |
|  | **RSA high**  **(N=303)** | **RSA low (N=276)** |  | **RSA high**  **(N=173)** | **RSA low (N=173)** |  |
| **Gender** |  |  | **0.318** |  |  | **1.000** |
| Male | **199(65.7%)** | **192(69.6%)** |  | **120(69.4%)** | **120(69.4%)** |  |
| Female | **104(34.3%)** | **84(30.4%)** |  | **53(30.6%)** | **53(30.6%)** |  |
| **Age, years** |  |  | **0.004** |  |  | **0.643** |
| ≤65 | **107(35.3%)** | **130(47.1%)** |  | **52(30.1%)** | **56(32.4%)** |  |
| ＞65 | **196(64.7%)** | **146(52.9%)** |  | **121(69.9%)** | **117(67.6%)** |  |
| **ECOG PS** |  |  | **0.788** |  |  | **0.665** |
| 0-1 | **145(47.9%)** | **129(46.7%)** |  | **79(45.7%)** | **75(43.4%)** |  |
| 2 | **158(52.1%)** | **147(53.3%)** |  | **94(54.3%)** | **98(56.6%)** |  |
| **Primary site** |  |  | **0.256** |  |  | **0.989** |
| Up 1/3 | **100(33.0%)** | **99(35.9%)** |  | **53(30.6%)** | **52(30.1%)** |  |
| Middle 1/3 | **62(20.5%)** | **42(15.2%)** |  | **37(21.4%)** | **38(22.0%)** |  |
| Lower 1/3 | **141(46.5%)** | **135(48.9%)** |  | **83(48.0%)** | **83(48.0%)** |  |
| **Tumor size, cm** |  |  | **0.674** |  |  | **0.666** |
| ≤5 | **144(47.5%)** | **136(49.3%)** |  | **78(45.1%)** | **82(47.4%)** |  |
| ＞5 | **159(52.5%)** | **140(50.7%)** |  | **95(54.9%)** | **91(52.6%)** |  |
| **Histology** |  |  | **0.036** |  |  | **0.828** |
| None/Low | **168(55.4%)** | **129(46.7%)** |  | **97(56.1%)** | **99(57.2%)** |  |
| High/Median | **135(44.6%)** | **147(53.3%)** |  | **76(43.9%)** | **74(42.8%)** |  |
| **SII^*^** |  |  | **0.998** |  |  | **0.830** |
| Low | **168(55.4%)** | **153(55.4%)** |  | **87(50.3%)** | **89(51.4%)** |  |
| High | **135(44.6%)** | **123(44.6%)** |  | **86(49.7%)** | **84(48.6%)** |  |
| **PNI^*^** |  |  | **0.227** |  |  | **1.000** |
| Low | **60(19.8%)** | **44(15.9%)** |  | **33(19.1%)** | **33(19.1%)** |  |
| High | **243(80.2%)** | **232(84.1%)** |  | **140(80.9%)** | **140(80.9%)** |  |
| **NLR^*^** |  |  | **0.167** |  |  | **0.830** |
| Low | **165(54.5%)** | **166(60.1%)** |  | **86(49.7%)** | **84(48.6%)** |  |
| High | **138(45.5%)** | **110(39.9%)** |  | **87(50.3%)** | **89(51.4%)** |  |
| **PLR^*^** |  |  | **0.967** |  |  | **0.747** |
| Low | **140(46.2%)** | **128(46.4%)** |  | **88(50.9%)** | **85(49.1%)** |  |
| High | **163(53.8%)** | **148(53.6%)** |  | **85(49.1%)** | **88(50.9%)** |  |
| **Variable** | **TNM stage III** | | | | | |
|  | **Before PSM** | | ***P* value** | **After PSM** | | ***P* value** |
|  | **RSA high**  **(N=520)** | **RSA low (N=355)** |  | **RSA high**  **(N=266)** | **RSA low (N=266)** |  |
| **Gender** |  |  | **0.550** |  |  | **0.587** |
| Male | **337(64.8%)** | **237(66.8%)** |  | **174(65.4%)** | **168(63.2)** |  |
| Female | **183(35.2%)** | **118(33.2%)** |  | **92(34.6%)** | **98(36.8%)** |  |
| **Age, years** |  |  | **0.859** |  |  | **0.095** |
| ≤65 | **217(41.7%)** | **146(41.1%)** |  | **122(45.9%)** | **103(38.7%)** |  |
| ＞65 | **303(58.3%)** | **209(58.9%)** |  | **144(54.1%)** | **163(61.3%)** |  |
| **ECOG PS** |  |  | **0.902** |  |  | **0.862** |
| 0-1 | **260(50.0%)** | **179(50.4%)** |  | **131(49.2%)** | **133(50.0%)** |  |
| 2 | **260(50.0%)** | **176(49.6%)** |  | **135(50.8%)** | **133(50.0%)** |  |
| **Primary site** |  |  | **0.006** |  |  | **0.552** |
| Up 1/3 | **158(30.4%)** | **110(31.0%)** |  | **84(31.6%)** | **81(30.5%)** |  |
| Middle 1/3 | **64(12.3%)** | **70(19.7%)** |  | **50(18.8%)** | **42(15.8%)** |  |
| Lower 1/3 | **298(57.3%)** | **175(49.3%)** |  | **132(49.6%)** | **143(53.8%)** |  |
| **Tumor size, cm** |  |  | **0.144** |  |  | **0.931** |
| ≤5 | **281(54.0%)** | **174(49.0%)** |  | **138(51.9%)** | **137(51.5%)** |  |
| ＞5 | **239(46.0%)** | **181(51.0%)** |  | **128(48.1%)** | **129(48.5%)** |  |
| **Histology** |  |  | **0.638** |  |  | **0.140** |
| None/Low | **275(52.9%)** | **182(51.3%)** |  | **128(48.1%)** | **145(54.5%)** |  |
| High/Median | **245(47.1%)** | **173(48.7%)** |  | **138(51.9%)** | **121(45.5%)** |  |
| **SII^*^** |  |  | **0.189** |  |  | **0.486** |
| Low | **306(58.8%)** | **193(54.4%)** |  | **142(53.4%)** | **150(56.4%)** |  |
| High | **214(41.2%)** | **162(45.6%)** |  | **124(46.6%)** | **116(43.6%)** |  |
| **PNI^*^** |  |  | **0.260** |  |  | **0.221** |
| Low | **133(25.6%)** | **79(22.3%)** |  | **69(25.9%)** | **57(21.4%)** |  |
| High | **387(74.4%)** | **276(77.7%)** |  | **197(74.1%)** | **209(78.6%)** |  |
| **NLR^*^** |  |  | **0.230** |  |  | **0.861** |
| Low | **317(61.0%)** | **202(56.9%)** |  | **151(56.8%)** | **149(56.0%)** |  |
| High | **203(39.0%)** | **153(43.1%)** |  | **115(43.2%)** | **117(44.0%)** |  |
| **PLR^*^** |  |  | **0.910** |  |  | **0.083** |
| Low | **247(47.5%)** | **170(47.9%)** |  | **118(44.4%)** | **138(51.9%)** |  |
| High | **273(52.5%)** | **185(52.1%)** |  | **148(55.6%)** | **128(48.1%)** |  |

**Note:** ECOG PS = Eastern Cooperative Oncology Group Performance Status; SII = Systemic Immune-inflammation Index; PNI = Prognostic Nutritional Index; NLR = Neutrophil to Lymphocyte Ratio; PLR = Platelet to Lymphpcyte Ratio. * Divided into high and low groups using the median as the critical value.

**Table S29. Univariate and multivariate Cox regression analysis of the factors affecting the 5-year overall survival rate of patients with LAGC in TNM stage I after surgery.**

| **Variable** | **Univariate analysis** | |  | **Multivariate analysis** | |
| --- | --- | --- | --- | --- | --- |
|  | **HR (95%CI)** | ***P* value** |  | **HR (95%CI)** | ***P* value** |
| **Gender** |  | **0.755** |  |  |  |
| Male | **Reference** |  |  |  |  |
| Female | **1.236 (0.326-4.684)** |  |  |  |  |
| **Age, years** |  | **0.454** |  |  |  |
| ≤65 | **Reference** |  |  |  |  |
| ＞65 | **1.478 (0.532-4.106)** |  |  |  |  |
| **ECOG PS** |  | **0.293** |  |  |  |
| 2 | **Reference** |  |  |  |  |
| 0-1 | **0.561 (0.191-1.646)** |  |  |  |  |
| **Primary site** |  | **0.123** |  |  |  |
| Up 1/3 | **Reference** |  |  |  |  |
| Middle 1/3 | **0.554 (0.175-1.755)** | **0.315** |  |  |  |
| Lower 1/3 | **0.072 (0.006-0.914)** | **0.042** |  |  |  |
| **Tumor size, cm** |  | **0.075** |  |  |  |
| ＞5 | **Reference** |  |  |  |  |
| ≤5 | **0.369 (0.123-1.107)** |  |  |  |  |
| **Histology** |  | **0.009** |  |  | **0.058** |
| None/Low | **Reference** |  |  | **Reference** |  |
| High/Median | **0.169 (0.045-0.637)** |  |  | **0.346 (0.116-1.036)** |  |
| **SII^*^** |  | **0.457** |  |  |  |
| Low | **Reference** |  |  |  |  |
| High | **1.527 (0.501-4.658)** |  |  |  |  |
| **PNI^*^** |  | **0.457** |  |  |  |
| High | **Reference** |  |  |  |  |
| Low | **1.527 (0.501-4.658)** |  |  |  |  |
| **NLR^*^** |  | **0.539** |  |  |  |
| Low | **Reference** |  |  |  |  |
| High | **1.691 (0.317-9.036)** |  |  |  |  |
| **PLR^*^** |  | **0.171** |  |  |  |
| High | **Reference** |  |  |  |  |
| Low | **0.426 (0.126-1.444)** |  |  |  |  |
| **Recurrence** |  | **0.668** |  |  |  |
| Yes | **Reference** |  |  |  |  |
| No | **0.791 (0.270-2.311)** |  |  |  |  |
| **RSA model score** |  | **0.001** |  |  | **0.010** |
| Low | **Reference** |  |  | **Reference** |  |
| High | **9.869 (2.601-37.443)** |  |  | **3.810 (1.384-10.487)** |  |

**Note:** ECOG PS = Eastern Cooperative Oncology Group Performance Status; SII = Systemic Immune-inflammation Index; PNI = Prognostic Nutritional Index; NLR = Neutrophil to Lymphocyte Ratio; PLR = Platelet to Lymphpcyte Ratio. * Divided into high and low groups using the median as the critical value.

**Table S30. Univariate and multivariate Cox regression analysis of the factors affecting the 5-year overall survival rate of patients with LAGC in TNM stage II after surgery.**

| **Variable** | **Univariate analysis** | |  | **Multivariate analysis** | |
| --- | --- | --- | --- | --- | --- |
|  | **HR (95%CI)** | ***P* value** |  | **HR (95%CI)** | ***P* value** |
| **Gender** |  | **0.251** |  |  |  |
| Male | **Reference** |  |  |  |  |
| Female | **1.231 (0.863-1.757)** |  |  |  |  |
| **Age, years** |  | **0.722** |  |  |  |
| ≤65 | **Reference** |  |  |  |  |
| ＞65 | **0.938 (0.659-1.334)** |  |  |  |  |
| **ECOG PS** |  | **0.070** |  |  |  |
| 2 | **Reference** |  |  |  |  |
| 0-1 | **0.736 (0.529-1.025)** |  |  |  |  |
| **Primary site** |  | **0.454** |  |  |  |
| Up 1/3 | **Reference** |  |  |  |  |
| Middle 1/3 | **1.088 (0.740-1.600)** | **0.667** |  |  |  |
| Lower 1/3 | **1.316 (0.857-2.021)** | **0.209** |  |  |  |
| **Tumor size, cm** |  | **0.057** |  |  |  |
| ＞5 | **Reference** |  |  |  |  |
| ≤5 | **0.718 (0.511-1.009)** |  |  |  |  |
| **Histology** |  | **0.858** |  |  |  |
| None/Low | **Reference** |  |  |  |  |
| High/Median | **0.969 (0.691-1.360)** |  |  |  |  |
| **SII^*^** |  | **0.149** |  |  |  |
| High | **Reference** |  |  |  |  |
| Low | **0.742 (0.495-1.112)** |  |  |  |  |
| **PNI^*^** |  | **0.462** |  |  |  |
| Low | **Reference** |  |  |  |  |
| High | **0.854 (0.560-1.301)** |  |  |  |  |
| **NLR^*^** |  | **0.169** |  |  |  |
| Low | **Reference** |  |  |  |  |
| High | **1.326 (0.887-1.982)** |  |  |  |  |
| **PLR^*^** |  | **0.928** |  |  |  |
| High | **Reference** |  |  |  |  |
| Low | **0.983 (0.683-1.415)** |  |  |  |  |
| **Recurrence** |  | **0.872** |  |  |  |
| Yes | **Reference** |  |  |  |  |
| No | **0.972 (0.684-1.380)** |  |  |  |  |
| **RSA model score** |  | **＜0.001** |  |  | **＜0.001** |
| Low | **Reference** |  |  | **Reference** |  |
| High | **1.908 (1.353-2.691)** |  |  | **1.848 (1.323-2.581)** |  |

**Note:** ECOG PS = Eastern Cooperative Oncology Group Performance Status; SII = Systemic Immune-inflammation Index; PNI = Prognostic Nutritional Index; NLR = Neutrophil to Lymphocyte Ratio; PLR = Platelet to Lymphpcyte Ratio. * Divided into high and low groups using the median as the critical value.

**Table S31. Univariate and multivariate Cox regression analysis of the factors affecting the 5-year overall survival rate of patients with LAGC in TNM stage III after surgery.**

| **Variable** | **Univariate analysis** | |  | **Multivariate analysis** | |
| --- | --- | --- | --- | --- | --- |
|  | **HR (95%CI)** | ***P* value** |  | **HR (95%CI)** | ***P* value** |
| **Gender** |  | **0.032** |  |  | **0.037** |
| Male | **Reference** |  |  | **Reference** |  |
| Female | **0.766 (0.599-0.978)** |  |  | **0.775 (0.610-0.985)** |  |
| **Age, years** |  | **0.819** |  |  |  |
| ≤65 | **Reference** |  |  |  |  |
| ＞65 | **1.028 (0.815-1.296)** |  |  |  |  |
| **ECOG PS** |  | **0.681** |  |  |  |
| 0-1 | **Reference** |  |  |  |  |
| 2 | **1.049 (0.836-1.316)** |  |  |  |  |
| **Primary site** |  | **0.259** |  |  |  |
| Up 1/3 | **Reference** |  |  |  |  |
| Middle 1/3 | **0.826 (0.636-1.073)** | **0.152** |  |  |  |
| Lower 1/3 | **0.825 (0.600-1.134)** | **0.236** |  |  |  |
| **Tumor size, cm** |  | **0.412** |  |  |  |
| ≤5 | **Reference** |  |  |  |  |
| ＞5 | **1.100 (0.876-1.381)** |  |  |  |  |
| **Histology** |  | **0.297** |  |  |  |
| None/Low | **Reference** |  |  |  |  |
| High/Median | **0.884 (0.702-1.114)** |  |  |  |  |
| **SII^*^** |  | **0.660** |  |  |  |
| Low | **Reference** |  |  |  |  |
| High | **1.059 (0.789-1.306)** |  |  |  |  |
| **PNI^*^** |  | **0.701** |  |  |  |
| High | **Reference** |  |  |  |  |
| Low | **1.055 (0.802-1.389)** |  |  |  |  |
| **NLR^*^** |  | **0.041** |  |  | **0.032** |
| High | **Reference** |  |  | **Reference** |  |
| Low | **0.765 (0.592-0.988)** |  |  | **0.779 (0.621-0.978)** |  |
| **PLR^*^** |  | **0.745** |  |  |  |
| High | **Reference** |  |  |  |  |
| Low | **0.960 (0.750-1.229)** |  |  |  |  |
| **Recurrence** |  | **0.909** |  |  |  |
| No | **Reference** |  |  |  |  |
| Yes | **1.015 (0.789-1.306)** |  |  |  |  |
| **RSA model score** |  | **＜0.001** |  |  | **＜0.001** |
| Low | **Reference** |  |  | **Reference** |  |
| High | **2.228 (1.764-2.813)** |  |  | **2.222 (1.766-2.796)** |  |

**Note:** ECOG PS = Eastern Cooperative Oncology Group Performance Status; SII = Systemic Immune-inflammation Index; PNI = Prognostic Nutritional Index; NLR = Neutrophil to Lymphocyte Ratio; PLR = Platelet to Lymphpcyte Ratio. * Divided into high and low groups using the median as the critical value

**Table S32. Different radiomics and pathomic feature combinations and their corresponding organ tendencies**

| **Metastatic sites** | **Radiomics and pathomic feature combinations** | **ROC** |
| --- | --- | --- |
| **Hematogenous** | **Feature-276+Feature-462+ Feature-480+Feature-407 + Feature-48+ Feature-13** | **0.830** |
| **Implantation** | **Feature-365+ Feature-480 + Feature-407 + Feature-192** | **0.806** |
| **Lymphatic** | **Feature-276 + Feature-462 + Feature-480 + Feature-407 +Feature-192 + Feature-502** | **0.828** |
| **Local** | **Feature-365 + Feature-192 + Feature-502** | **0.713** |

**Table S33. Clinical and pathological characteristics of patients in the TCIA dataset**

| **Variable** | **Total**  **(N=41)** | **TCIA cohort** | | **P value** |
| --- | --- | --- | --- | --- |
|  |  | **Risk-high (N=15)** | **Risk-low (N=26)** |  |
| **Gender** |  |  |  | **0.232*** |
| Male | **34(82.9%)** | **14(93.3%)** | **20(76.9%)** |  |
| Female | **7(17.1%)** | **1(6.7%)** | **6(23.1%)** |  |
| **Race** |  |  |  | **0.767** |
| White | **27(65.9%)** | **11(73.3%)** | **16(61.5%)** |  |
| Black | **5(12.2%)** | **1(6.7%)** | **4(15.4%)** |  |
| Asian | **2(4.9%)** | **1(6.7%)** | **1(3.8%)** |  |
| Unknown | **7(17.1%)** | **2(13.3%)** | **5(19.2%)** |  |
| **Primary site** |  |  |  | **0.933** |
| Antrum/Distal | **11(26.8%)** | **4(26.7%)** | **7(26.9%)** |  |
| Cardia/Proximal | **9(22.0%)** | **4(26.7%)** | **5(19.2%)** |  |
| Fundus/Body | **13(31.7%)** | **4(26.7%)** | **9(34.6%)** |  |
| Gastroesophageal Junction | **8(19.5%)** | **3(20.0%)** | **5(19.2%)** |  |
| **Family history of stomach cancer** |  |  |  | **0.651*** |
| Yes | **6(14.6%)** | **3(20.0%)** | **3(11.5%)** |  |
| No | **35(85.4%)** | **12(80.0%)** | **23(88.5%)** |  |
| **pT stage** |  |  |  | **0.218** |
| T2/T3 | **25(61.0%)** | **11(73.3%)** | **14(53.8%)** |  |
| T4 | **16(39.0%)** | **4(26.7%)** | **12(46.2%)** |  |
| **pN stage** |  |  |  | **0.063*** |
| N0 | **10(24.4%)** | **1(6.7%)** | **9(34.6%)** |  |
| N+ | **31(75.6%)** | **14(93.3%)** | **17(65.4%)** |  |

**Note: *Fish's exact test**

**Table S34. Comparison of performance indicators of different models in predicting postoperative recurrence in patients with LAGC in training set**

| **Variable** | **AUC** | **Accuracy** | **Sensitivity** | **Specificity** | **PPV** | **NPV** | **PLR (x10)** | **NLR** | **F1 score** |
| --- | --- | --- | --- | --- | --- | --- | --- | --- | --- |
| Clinical features | **0.757** | **0.732** | **0.950** | **0.524** | **0.655** | **0.917** | **0.095** | **0.776** | **0.200** |
| Radiomics | **0.743** | **0.780** | **0.750** | **0.810** | **0.789** | **0.773** | **0.309** | **0.769** | **0.394** |
| Pathomics | **0.807** | **0.756** | **0.800** | **0.714** | **0.727** | **0.789** | **0.280** | **0.762** | **0.280** |
| RSA model | **0.898** | **0.829** | **0.700** | **0.952** | **0.933** | **0.769** | **0.315** | **0.800** | **1.470** |

Note: AUC-area under the curve; PPV-Positive Predictive Value; NVP-Negative Predictive Value; PLR-Positive Likelihood Ratio; NLR-Negative Likelihood Ratio.

**Table S35. Proportion of different models in predicting postoperative recurrence in LAGC patients**

| **Sequence** | **Clinical features vs. RSA model** | **Radiomics vs. RSA Model** | **Pathomics vs. RSA models** |
| --- | --- | --- | --- |
| **Training set** |  |  |  |
| **NRI (95%CI)** | **0.357 (-0.191-0.905)** | **0.186 (-0.317-0.708)** | **0.276 (-0.335-0.900)** |
| **IDI (95%CI)** | **0.269 (0.134-0.405)** | **0.301 (0.169-0.414)** | **0.208 (0.028-0.375)** |
| **DeLong Test** | **0.045** | **0.114** | **0.045** |

**Supplementary Methods**

**Inclusion and Exclusion Criteria:**

**Patients were eligible for inclusion if they met all of the following conditions: (1) age 18 years or older; (2) had undergone contrast-enhanced CT scanning within 14 days before surgical intervention; (3) received a postoperative pathological diagnosis confirming gastric cancer; and (4) had undergone curative-intent surgical resection. Participants were excluded based on the following criteria: (1) prior administration of neoadjuvant therapy, including chemotherapy, targeted agents, or immunotherapy before surgery; (2) death attributable to surgical complications occurring within 90 days post-surgery; (3) absence of follow-up data; (4) presence of substantial CT image artifacts or unavailability of imaging data; and (5) incomplete clinical records.**

**Clinical Data Collection**
Comprehensive demographic and clinical variables recorded within 14 days before surgery were retrieved from the hospital’s electronic medical record system. These variables included: age, gender, ECOG performance status, peripheral neutrophil and lymphocyte counts, platelet levels, serum albumin, and tumor biomarkers such as carcinoembryonic antigen (CEA), carbohydrate antigen 19-9 (CA19-9), and carbohydrate antigen 72-4 (CA72-4). Information on postoperative therapeutic interventions was also documented.

Pathologic parameters were collected from formal pathology reports, encompassing tumor grade, nodal involvement, perineural and lymphovascular invasion, resection margin status, tumor dimensions and anatomical location, as well as immunohistochemical expression of HER2 and PD-L1. Tumor staging was reviewed and standardized based on the 8th edition criteria set by the American Joint Committee on Cancer (AJCC). Additionally, the following inflammatory and nutritional indices were computed: Neutrophil-to-lymphocyte ratio (NLR = neutrophils / lymphocytes); Platelet-to-lymphocyte ratio (PLR = platelets / lymphocytes); Prognostic nutritional index (PNI = albumin \[g/L] + 5 × lymphocyte count); Systemic immune-inflammation index (SII = platelets × neutrophils / lymphocytes).

**CT Acquisition Protocols by Participating Centers**

Hebei Province centers (FHHMU, SJZPH, BDCH, HSPH): CT examinations were performed using six multidetector scanners (three 256-detector and three 128-detector systems). All patients received 800–1000 mL of water orally 30 minutes before scanning. Scanning parameters included a tube voltage of 120 kV, and tube current controlled via automatic mAs modulation. The scanning range covered the area from the diaphragm dome to the pubic symphysis. Contrast-enhanced scans used 300 mg/mL nonionic contrast medium at 3.0 mL/s, with a total dose of 2 mL/kg. Venous phase images were acquired 70 seconds after injection, and arterial phase at 25 seconds. Image reconstruction was performed with 1.0 mm slice thickness.

Nanjing Jinling Hospital: Scans were performed on dual-source spiral CT scanners. Parameters included 120 kVp tube voltage, 230 mA tube current, 0.5-second rotation time, and a pitch of 1.2. Contrast medium was injected at 1.5 mL/kg with a rate of 3–4 mL/s. Venous phase images were obtained at 60 seconds, with delayed scans at 180 seconds when clinically indicated. Images were reconstructed at 1.0–1.5 mm thickness, with a 35–50 cm field of view and matrix size of 512×512.

Wuhan University Renmin Hospital: A 256-slice CT scanner was used. Parameters included 120 kV tube voltage, 200 mA tube current, and 0.5-second rotation time. Contrast medium (1.5 mL/kg, 300 mg/mL iodine) was administered at 3–4 mL/s. Venous phase images were acquired at 70 seconds. Reconstruction was performed with 0.625 mm slice thickness and a 512×512 matrix.

**Evaluation of Model Complexity and Overfitting Risk**

To evaluate the risk of overfitting associated with high-dimensional radiomic features, we conducted two complementary analyses: learning curve analysis and feature subset performance assessment. (1) Learning curve analysis: To assess model generalizability under different training sizes, we randomly sampled increasing proportions (10%, 20%, ..., 90%) of the full training cohort and retrained the ensemble model at each level. At every step, model performance was evaluated using 10-fold cross-validation. AUC values were calculated for both the training and validation folds, and results were averaged across folds to generate the learning curve. Standard deviations were plotted as error bars. The learning curve was used to examine convergence behavior and to detect potential overfitting, reflected by large discrepancies between training and validation performance. (2) Feature subset performance analysis: To investigate the relationship between feature dimensionality and model performance, we ranked the selected radiomic features based on their importance (mean absolute SHAP value in the training set). Models were then iteratively constructed using the top k features (k = 1 to 20), and each model was evaluated using 10-fold cross-validation to compute the average AUC at each feature subset size. This allowed us to identify the optimal feature count where performance plateaued and beyond which adding additional features led to marginal gains or even performance decline. This analysis directly supports the appropriateness of the final feature set selected through mRMR and LASSO.

**Evaluation of ComBat Harmonization using PCA**

**PCA was performed using standardized radiomic feature matrices before and after ComBat correction. Principal components were calculated using the prcomp function in R. Data points were colored by acquisition center or dataset (training, validation, external). PCA plots were used to visualize the degree of batch-related clustering before and after harmonization.**

****Model Fusion Strategy****

**To construct the final Recurrence Stratification Assessment (RSA) model, we employed a score-level fusion strategy integrating three independent sources of information: (1) clinical predictors identified through multivariate logistic regression, (2) the dichotomized Rad-score derived from the radiomics signature, and (3) the dichotomized Path-score derived from the pathomic signature. These three components were entered into a multivariate logistic regression model to generate the RSA-derived recurrence risk score for each patient.**

**The RSA risk scores were subsequently stratified into high- and low-risk groups according to the optimal cutoff value determined in the training cohort using the Youden index. This fusion strategy effectively integrated multimodal information while maintaining model interpretability by preserving the independent contribution of each modality.**

**RNA Sequencing**

**RNA sequencing was conducted on 114 tumor tissue specimens from patients with gastric cancer. cDNA synthesis and library preparation followed a strand-specific protocol (Genedenovo, Guangzhou, China). The constructed libraries underwent quality assessment using agarose gel electrophoresis, NanoPhotometer, Qubit 2.0 Fluorometer, and the Agilent 2100 Bioanalyzer. Only libraries passing quality control were sequenced on the Illumina Novaseq X Plus platform. Raw sequencing reads were evaluated using the Fastp tool to assess data quality[1]. Alignment of reads to the reference genome was performed using HISAT2[2]. Based on aligned data, StringTie was applied to reconstruct transcripts, and gene-level expression quantification was carried out using RSEM[3-4].**

**Differential Expression Analysis**

**To identify genes differentially expressed between RSA-defined high- and low-risk groups, the edgeR package was employed. The analysis used an overdispersed Poisson model with empirical Bayes shrinkage to account for biological variability. Gene expression was normalized as counts per million (CPM), and only genes with CPM > 2 in at least two samples were included in the final analysis. Differential expression was determined using generalized linear models and likelihood ratio tests. Genes were deemed significantly differentially expressed if the P-value was less than 0.05 and the absolute log₂ fold change exceeded 1.0.**

**Functional enrichment analyses**

Gene Set Enrichment Analysis (GSEA) was performed to investigate biological differences between gene signatures[5]. Fifty hallmark gene sets from the Molecular Signatures Database (MSigDB) were utilized[6]. Standard GSEA was conducted on normalized expression data, with 1,000 permutations. To account for multiple testing, P-values were adjusted using the Benjamini–Hochberg method to control the false discovery rate (FDR). Gene sets with FDR-adjusted P-values < 0.05 were considered significantly enriched.

**Evaluation of immune activity**

Tumor immune activity was assessed using the ESTIMATE algorithm, which calculates an immune score for each sample based on gene expression profiles to reflect the level of immune cell infiltration[7]. Differences in immune scores between high- and low-risk groups were compared using the R package limma. P-values were adjusted using the Benjamini–Hochberg method, and features with FDR-adjusted P-values < 0.05 were considered statistically significant.

**Immune infiltration**

CIBERSORTx was used to quantify the relative proportions of 22 infiltrating immune cell types in gastric cancer samples using the LM22 signature matrix [8]. Analysis was performed in "absolute" mode with 1,000 permutations. Gene expression data were normalized following CIBERSORTx guidelines, and batch correction was disabled. The absolute score enabled robust between-sample comparisons of immune cell content. P-values obtained from group comparisons were corrected using the Benjamini–Hochberg method, and immune cell types with FDR-adjusted P-values < 0.05 were considered statistically significant.

To further assess immune and stromal cell infiltration, we utilized MCPcounter [9], which estimates the abundance of eight immune cell populations, including CD4 positive T cells, CD8 positive T cells, natural killer cells, B lymphocytes, monocytes, myeloid dendritic cells, neutrophils, and cytotoxic lymphocytes, as well as two stromal cell types comprising fibroblasts and endothelial cells.

**Ecosystem Characterization using EcoTyper**

EcoTyper v1.0 [10,11] was used to infer immune-related cell states and multicellular ecosystems (CEs) from bulk RNA-seq data. The pipeline was executed using default parameters with pre-trained models available at https://ecotyper.stanford.edu/. Prior to analysis, expression matrices were log₂-transformed and quantile normalized to match the input requirements of the tool. EcoTyper identifies recurrent cell states and their co-occurrence patterns across carcinomas, which were then mapped in our gastric cancer cohort to elucidate immune ecosystem heterogeneity.

**Statistical analysis**

Categorical data were summarized using counts and percentages, with group comparisons performed via the chi-square test or Fisher’s exact test when appropriate. Continuous variables were reported as medians with interquartile ranges (IQRs), and differences between groups were assessed using the Mann–Whitney U test. Feature selection was implemented using the least absolute shrinkage and selection operator (LASSO) method through the glmnet package. To explore associations with early postoperative recurrence, univariate logistic regression was initially applied, followed by multivariate logistic regression to identify independent predictive variables.

Kaplan-Meier survival analysis was conducted to estimate survival distributions, with comparisons made using the log-rank test. Hazard ratios (HRs) were calculated from the regression outputs. Calibration curves were drawn to examine the consistency between predicted probabilities and actual survival outcomes. The rms package was used for regression modeling, nomogram development, and calibration assessment. To evaluate discrimination ability, the area under the receiver operating characteristic (ROC) curve (AUC) was calculated at predefined time intervals. Differences in AUC values across models were compared using the pROC package. All statistical analyses were two-sided, and P values less than 0.05 were deemed statistically significant.

**Reference**

[1]Langmead B, Salzberg SL. Fast gapped-read alignment with Bowtie 2. Nat Methods. 2012 Mar 4;9(4):357-9. doi: 10.1038/nmeth.1923.

[2]Kim D, Langmead B, Salzberg SL. HISAT: a fast spliced aligner with low memory requirements. Nat Methods. 2015 Apr;12(4):357-60. doi: 10.1038/nmeth.3317.

[3]Pertea M, Pertea GM, Antonescu CM, Chang TC, Mendell JT, Salzberg SL. StringTie enables improved reconstruction of a transcriptome from RNA-seq reads. Nat Biotechnol. 2015 Mar;33(3):290-5. doi: 10.1038/nbt.3122.

[4]Li B, Dewey CN. RSEM: accurate transcript quantification from RNA-Seq data with or without a reference genome. BMC Bioinformatics. 2011 Aug 4;12:323. doi: 10.1186/1471-2105-12-323.

[5]Bera K, Braman N, Gupta A, Velcheti V, Madabhushi A. Predicting cancer outcomes with radiomics and artificial intelligence in radiology. Nat Rev Clin Oncol. 2022 Feb;19(2):132-146. doi: 10.1038/s41571-021-00560-7.

[6]Wang Z, Fang M, Zhang J, Tang L, Zhong L, Li H, Cao R, Zhao X, Liu S, Zhang R, Xie X, Mai H, Qiu S, Tian J, Dong D. Radiomics and Deep Learning in Nasopharyngeal Carcinoma: A Review. IEEE Rev Biomed Eng. 2024;17:118-135. doi: 10.1109/RBME.2023.3269776.

[7]Yoshihara K, Shahmoradgoli M, Martínez E, Vegesna R, Kim H, Torres-Garcia W, Treviño V, Shen H, Laird PW, Levine DA, Carter SL, Getz G, Stemke-Hale K, Mills GB, Verhaak RG. Inferring tumour purity and stromal and immune cell admixture from expression data. Nat Commun. 2013;4:2612. doi: 10.1038/ncomms3612.

[8]Newman AM, Liu CL, Green MR, Gentles AJ, Feng W, Xu Y, Hoang CD, Diehn M, Alizadeh AA. Robust enumeration of cell subsets from tissue expression profiles. Nat Methods. 2015 May;12(5):453-7. doi: 10.1038/nmeth.3337.

[9]Becht E, Giraldo NA, Lacroix L, Buttard B, Elarouci N, Petitprez F, Selves J, Laurent-Puig P, Sautès-Fridman C, Fridman WH, de Reyniès A. Estimating the population abundance of tissue-infiltrating immune and stromal cell populations using gene expression. Genome Biol. 2016 Oct 20;17(1):218. doi: 10.1186/s13059-016-1070-5.

[10]Zhong L, Dong D, Fang X, Zhang F, Zhang N, Zhang L, Fang M, Jiang W, Liang S, Li C, Liu Y, Zhao X, Cao R, Shan H, Hu Z, Ma J, Tang L, Tian J. A deep learning-based radiomic nomogram for prognosis and treatment decision in advanced nasopharyngeal carcinoma: A multicentre study. EBioMedicine. 2021 Aug;70:103522. doi: 10.1016/j.ebiom.2021.103522.
